# High molecular weight dissolved organic matter drives soil resistome proliferation by enhancing microbial competition and viral carbon metabolism

**DOI:** 10.1093/ismejo/wrag212

**Published:** 2026-08-18

**Authors:** Zi-Teng Liu, Xin-Di Zhao, Jia-Qi Li, Shu-Xin Li, Xianjin Tang, Si-Yu Zhang

**Affiliations:** Zhejiang Zhoushan Island Observation and Research Station, State Key Laboratory of Estuarine and Coastal Research, School of Ecological and Environmental Sciences, East China Normal University, Shanghai 200241, China; Zhejiang Zhoushan Island Observation and Research Station, State Key Laboratory of Estuarine and Coastal Research, School of Ecological and Environmental Sciences, East China Normal University, Shanghai 200241, China; Zhejiang Zhoushan Island Observation and Research Station, State Key Laboratory of Estuarine and Coastal Research, School of Ecological and Environmental Sciences, East China Normal University, Shanghai 200241, China; Zhejiang Zhoushan Island Observation and Research Station, State Key Laboratory of Estuarine and Coastal Research, School of Ecological and Environmental Sciences, East China Normal University, Shanghai 200241, China; Zhejiang Provincial Key Laboratory of Agricultural Resources and Environment, College of Environmental and Resource Sciences, Zhejiang University, Hangzhou 310058, China; Zhejiang Zhoushan Island Observation and Research Station, State Key Laboratory of Estuarine and Coastal Research, School of Ecological and Environmental Sciences, East China Normal University, Shanghai 200241, China; Institute of Eco-Chongming (IEC), East China Normal University, Shanghai 202151, China

**Keywords:** dissolved organic matter, molecular weight, antibiotic resistance genes, carbon metabolism, agricultural soils

## Abstract

Soil organic carbon is a key determinant of microbial community structure and function, yet the role of dissolved organic matter (DOM) bioavailability in shaping the soil antibiotic resistome remains poorly understood. Here, we combined previous continental-scale field sampling across 18 provinces in China (*n* = 141) with additional microcosm experiments to investigate how DOM molecular weight influences soil antibiotic resistance gene (ARG) proliferation. Using Fourier transform ion cyclotron resonance mass spectrometry and metagenomic analyses, we found that soils enriched in high molecular weight (HMW) DOM harbored significantly greater ARG abundance and diversity compared to low molecular weight DOM soils. HMW DOM intensified microbial competition, as evidenced by a higher proportion of negative correlations in the co-occurrence network and lower niche breadth, favoring the enrichment of co-hosts that simultaneously carried ARGs, carbon metabolism genes, and biosynthetic gene clusters for antimicrobial compounds. Microcosm experiments confirmed that HMW DOM (lignin) addition significantly increased ARG transcript abundance (2.4-fold) and co-host relative abundance (2.3-fold), accompanied by a concurrent increase in transcribed viral auxiliary metabolic genes (2.5-fold) involved in complex carbon degradation. Structural equation modeling revealed that HMW DOM abundance and chemodiversity exerted the strongest positive effects on ARG abundance, primarily by shaping microbial community competition and metabolic potential. Collectively, our findings establish DOM bioavailability, particularly its molecular weight, as a critical yet previously overlooked driver of soil resistome development, challenging the conventional focus on total carbon content and highlighting the potential for molecular-level organic matter management to mitigate the spread of ARGs.

## Graphical Abstract

**Graphical Abstract:**
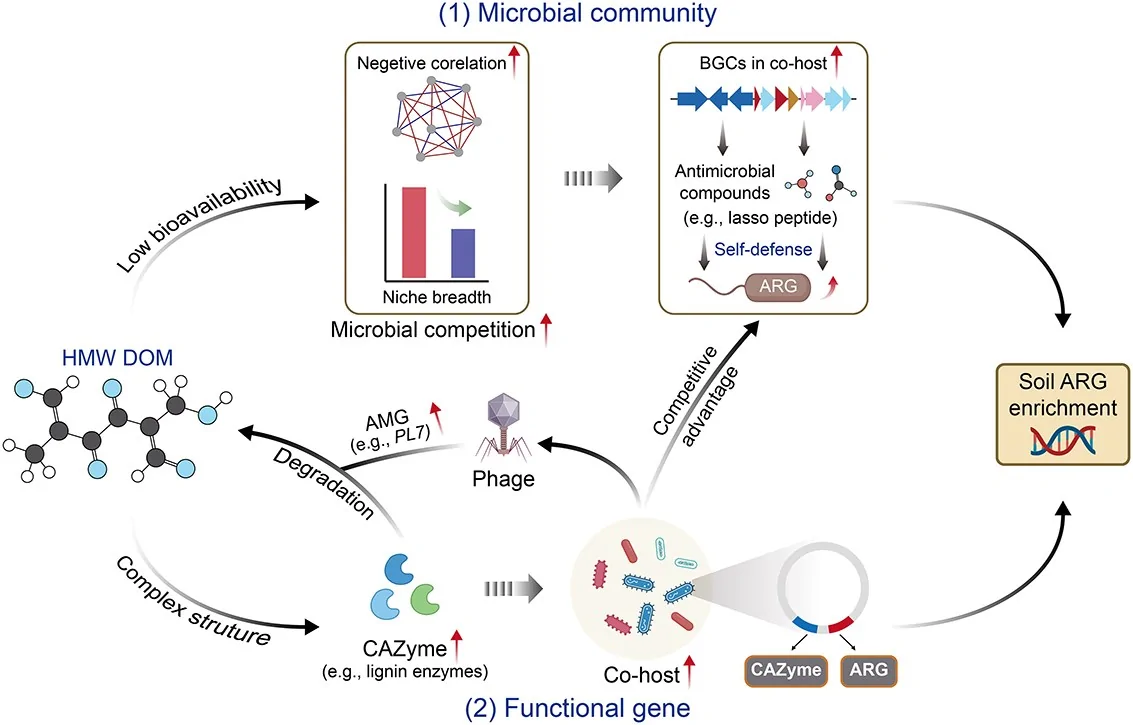

## Introduction

Antibiotic resistance genes (ARGs) have emerged through intrinsic evolutionary processes, as microorganisms developed resistance mechanisms to antimicrobial compounds synthesized by ecologically competing taxa [1, 2]. Competition for resources among microorganisms is therefore considered as a key driver in the evolution of resistance mechanisms [3, 4], with resource availability shaping microbial dynamics by driving niche partitioning and intensifying competition [5, 6].

Soil microbial communities are a key reservoir of ARGs [7], and environmental stresses from sewage [8], manure [9], and industrial wastes [10] drive global antimicrobial resistance dissemination by enabling ARG transfer to humans through the food chain, thereby exacerbating the health crisis. Soil organic carbon, a primary regulator of microbial growth, activity, and community composition [11, 12], is also a major factor influencing the abundance and diversity of ARGs in soils [13, 14]. Specifically, in carbon-limited soils, more aggressive microbial competition resulting in up-regulated expression of ARGs has been revealed [3]. Dissolved organic matter (DOM), as the most active component of soil organic carbon [15] and the main carbon source of microbes, exhibits significant correlations with soil microbial diversity [16–19], implying that DOM characteristics influence the structure and function of microbial communities [16]. For instance, *Pseudomonadota*, a predominant host of ARGs [20, 21], is significantly negatively correlated with aliphatic compounds but positively correlated with stable compounds [18, 19]. However, while the effects of DOM bioavailability on microbial interactions have been increasingly recognized [22], whether and how DOM bioavailability mediates the proliferation of ARGs remains unclear.

The bioavailability of DOM is largely determined by its molecular weight (MW) [22]. Low molecular weight (LMW) components (e.g. sugars, amino acids) are readily degraded, fueling rapid microbial growth, whereas high molecular weight (HMW) compounds (e.g. lignin, polysaccharides) require specialized enzymes and taxa for breakdown [16, 23]. This distinction in carbon utilization likely imposes differential selective pressures on microbial communities, potentially influencing the proliferation of ARGs. Furthermore, viruses can enhance host adaptation to carbon-limited environments by encoding auxiliary metabolic genes (AMGs) that optimize carbon processing [3, 24, 25]. Soils with low carbon availability show enriched AMGs associated with carbon degradation [26]. Viral lysis also facilitates microbial carbon competition by releasing labile organic matter [27] and can promote ARG dissemination via transduction, particularly chloramphenicol and macrolide-lincosamide-streptogramin (MLS) resistance genes [28]. Yet, the role of viruses in mediating microbial competition and ARG dynamics under different DOM bioavailability scenarios remains poorly understood.

To address these knowledge gaps, we hypothesize that DOM of different molecular weights differentially influences the proliferation of soil ARGs, with viruses potentially playing a key role in modulating host competitiveness under varying carbon availability. To test this hypothesis, we analyzed the correlation between relative abundance of ARGs and DOM characteristics using previously measured metagenomic and DOM molecular composition data (Fourier transform ion cyclotron resonance mass spectrometry, FT-ICR MS) from 141 soil samples collected across 18 provinces in China. We further profiled carbon metabolism genes (CMGs), carbohydrate-active enzymes (CAZymes) genes, and biosynthetic gene clusters (BGCs) within soil microbial communities. Additionally, we conducted microcosm experiments with HMW and LMW DOM amendments in soils and applied both metagenomic and metatranscriptomic analyses to validate the causal effects of DOM bioavailability on ARGs accumulation. Our study aimed to: (i) investigate the association between different DOM MW groups and ARG enrichment, (ii) elucidate the mechanisms by which DOM MW influences ARG proliferation, and (iii) clarify the role of viruses in enhancing host competitiveness and adaptation to carbon sources of varying molecular weight.

## Materials and methods

### Sample collection, metagenomic sequencing, and dissolved organic matter molecular traits calculation

Soil samples were collected from paddy soils across four major rice-growing regions of China: North China (Heilongjiang, Jilin, Liaoning, Tianjin), East China (Shandong, Jiangsu, Anhui, Shanghai, Zhejiang, Fujian), Central China (Henan, Hubei, Hunan, Jiangxi), and South China (Guangdong, Guangxi, Guizhou, Yunnan) from August 2022 to July 2023 [29] (Fig. S1). Based on field investigations and farmer interviews conducted during sampling, these fields received only chemical fertilizers or organic fertilizers that comply with the national standard NY/T 525-2021 in China. No sludge or manure was directly applied to any of the fields. A total of 141 surface soil samples (0–20 cm) were obtained following rice harvest, covering representative rice-growing provinces in each region. The collected soil samples were divided into three portions, which were used for DNA extraction, soil chemical properties determination, and DOM analysis, respectively.

Soil DNA was extracted from field samples using the ALFA-SEQ Advanced Soil DNA Kit (Findrop, China). The extracted DNA was fragmented to 400 bp using Covaris M220 (Gene, China) and used for paired-end library preparation with the NEXTFLEX Rapid DNA-Seq kit (Bioo Scientific, USA). All libraries were sequenced (2 × 150 bp) on a NovaSeq 6000 System (Illumina, USA). Basic information of 141 metagenomic data was provided in Table S1.

Soil chemical properties, including total organic carbon (TOC), total nitrogen (TN), total phosphorus (TP), total sulfur (TS), and pH, were determined. Details on the determination of soil chemical properties and DOM extraction were provided in the Supplementary methods. Six molecular traits of DOM, including double bond equivalents (DBE), nominal oxidation state of carbon (NOSC), modified aromaticity index (AI_mod_), oxygen–carbon ratio (O/C), hydrogen-carbon ratio (H/C), and MW, were calculated based on the elemental composition of DOM molecules, and intensity-weighted means were taken as the molecular traits of the samples (DBE_W_, NOSC_W_, (AI_mod_)_W_, O/C_W_, H/C_W_, and MW_W_). Additionally, the molecular lability boundary (MLB) was a widely used index based on the H/C values of DOM molecular formulae that characterizes DOM molecular stability and bioavailability [30]. The threshold of “H/C = 1.5” was widely accepted and serves as a relatively unbiased method for distinguishing between more bioavailable (H/C > 1.5) and less labile, more recalcitrant (H/C < 1.5) DOM molecules [31]. In this study, we used the MLB_R_ index to characterize the proportion of DOM molecules (H/C < 1.5) that are relatively more stable and recalcitrant. The chemodiversity of DOM was calculated using the Chao1 index through the R package *vegan* v2.6–10, with individual DOM molecules as species and the relative peak intensity as the abundance of each species. The details of the calculations were provided in Table S2.

### Sample grouping based on dissolved organic matter MW_W_

Soil samples were categorized into LMW (*n* = 87) and HMW (*n* = 54) groups based on whether the MW_W_ of DOM in the soils was greater than 400 Da (Table S1 and Fig. S1). The 400 Da molecular weight threshold was chosen based on recently published research [32–34], in which this criterion was used to distinguish between high- and low-molecular-weight DOM (details on the grouping were provided in the Supplementary methods). To further ensure the robustness of our conclusions, we conducted a sensitivity analysis using alternative molecular weight thresholds (390 Da, 395 Da, 405 Da, 410 Da, and 415 Da) and re-ran the primary statistical analyses (relative abundance of ARGs) based on linear mixed-effects models (LMMs). The results consistently confirmed that relative abundance of ARGs in HMW soils remained significantly higher than that in LMW soils across all tested thresholds (*β* = 0.41–0.72, *P* < .05; Table S3). Based on these consistent findings, we selected 400 Da as the optimal cutoff for DOM molecular weight grouping in all subsequent analyses.

### Data preprocessing, taxonomic classification, and functional gene annotation

The low-quality reads of metagenomic raw reads were removed by multitrim (https://github.com/KGerhardt/multitrim) to obtain clean reads. Nonpareil v3.303 [35] was employed to calculate metagenome coverage based on read redundancy. The genome equivalents (GEQ) for each sample were calculated by MicrobeCensus v1.1.0 [36].

Taxonomic classification of clean reads was conducted using Kraken2 v2.07 [37] with Bracken v2.7 [38] for relative abundance estimation. Microbial co-occurrence networks were constructed via the R package *WGCNA*, and niche breadth was calculated using the R package *vegan* v2.6-10 (detailed in Supplementary methods).

ARGs and high-risk ARGs (Rank I and Rank II) were annotated with default arguments by ARGs-OAP v3.2 [39] and arg_ranker v3.0 [40], respectively. CMGs and CAZyme genes were annotated against the Kyoto Encyclopedia of Genes and Genomes (KEGG) database and CAZy database by BLASTx of DIAMOND v2.0.14.152 [41], with thresholds of amino acid identity ≥80%, alignment length ≥ 25 aa, and e-value ≤1e-5. To reliably compare the diversity or abundance of features (e.g. genes) between different metagenomic samples, subsampling was performed based on the lowest metagenome coverage in all samples using Nonpareil v3.303. Details on subsampling of metagenomic data were described in the Supplementary methods. Relative abundance of ARGs was normalized to copies per cell [42] by ARGs-OAP v3.2. Relative abundances of CMGs and CAZyme genes were calculated from subsampled metagenomic datasets and normalized as RPKG = [(reads mapped to gene)/(gene length in kb)/(GEQ)], which indicates the fraction of total cells encoding the gene of interest (i.e. copies per cell) [9].

### Co-assembly, open reading frames, and antibiotic resistance gene host prediction

The clean reads from each sampling site (three samples) were co-assembled into contigs using MEGAHIT v1.2.9 [43]. Only contigs with a length >1000 bp were retained. Open reading frames (ORFs) from contigs were predicted using Prodigal v2.6.3 [44] (-p meta). CMGs were determined using KofamScan v1.1.0 [45] based on profile Hidden Markov Model (HMM) with adaptive score thresholds. CAZyme genes were annotated using dbCAN3 v4.1.3 [46] with default parameters, and substrates were sourced from the CAZy database. CAZyme genes involved in degrading complex organic matter, including polysaccharides [glycoside hydrolase (GH) enzymes] [47], chitin, lignin, and cellulose, were selected for further analysis.

Potential ARGs were identified using BLASTp of DIAMOND against the Structured Antibiotic Resistance Genes (SARG) database v3.2 [39] with thresholds of identity ≥60%, query coverage ≥70%, and e-value ≤1e-5. The contigs containing ARG-like ORFs were classified using Kraken2 to predict the taxonomy of ARG hosts. To identify potential pathogenic hosts, we compared the predicted host taxonomy to the human pathogen list (Table S4). This list (containing 821 human pathogens) was a compilation of human pathogens identified in previous studies [48–50] and Pathogen–Host Interactions Database (PHI-base; host = human) [51]. Putative pathogens were identified based on a species-level match to the human pathogen list. After that, the relative abundance of pathogen-associated ARGs was normalized as RPKG.

### Binning based on a co-assembling approach and biosynthetic gene clusters prediction

To acquire higher quality contigs and metagenome-assembled genomes (MAGs), co-assembling approach was applied. MAGs were recovered by MetaWRAP [52] and BASALT [53], respectively. The results of the merged MAGs were dereplicated using dRep v3.3.0 [54] (-sa 0.95 -nc 0.30) [55]. Only high-quality MAGs with completeness ≥90% and contamination ≤5% were included in the succeeding analysis. Taxonomy affiliation of MAGs was determined by GTDB-Tk v2.1.0 [56] with Genome Taxonomy Database (r214). Phylogenetic analysis of MAGs was conducted with the infer model of GTDB-Tk, and the phylogenetic tree was visualized in tvBOT [57]. The relative abundance of MAGs was calculated as TAD80 (Truncated Average Depth at 80%) divided by GEQ [58] (detailed in Supplementary methods). BGCs were identified from MAGs using antiSMASH v7.1.0 [59] (--fullhmmer --cb-general --cb-subclusters --cb-knownclusters --genefinding-tool prodigal-m --clusterhmmer --asf --smcog-trees --pfam2go) [60] and further clustered into families with BiG-SCAPE v1.1.8 [61] (--mode auto --mibig), including ribosomally synthesized and posttranslationally modified peptides (RiPPs), Terpene, nonribosomal synthetic peptides (NRPS), polyketide synthases I (PKS I), polyketide-nonribosomal peptide combinations (PKS-NRPS hybrids), other PKS, and Others. The niche overlap scores based on the carbon degradation function (CAZyme gene) for MAGs were performed following a recent study [62], and a detailed calculation process was provided in the Supplementary methods.

### Microcosm experiments with dissolved organic matter addition

Based on DOM characteristics from the continental-scale soil survey, soils from Huaihua-Hunan (HN) and Hongtang-Fujian (FJ) were selected as the representatives of the LMW and HMW DOM soils, respectively. HN and FJ soils were chosen because their DOM MW_W_ fall within the dominant frequency interval of LMW (374–389 Da) and HMW (412–439 Da) groups, respectively (Fig. S2). Lignin and glucose were selected as the representative HMW and LMW DOM. Three treatments, including control, lignin (CAS: 8068-05-1, MW: 505.01), and glucose (CAS: 50-99-7, MW: 180.16) addition, were established for each soil type with three replicates (Fig. S3). Each microcosm contained 40 g soil (<2 mm) mixed with 75 ml deionized water in 150 ml black PE containers, pre-incubated at 25°C in darkness for 7 days. DOM was added at 0.5% of air-dried soil weight (0.2 g), dissolved in 5 ml deionized water, and mixed thoroughly. Soil samples were collected before addition (Day 0) and on Days 1, 7, and 15 post-addition (Table S5). A total of 44 soil samples were collected. Detailed information on soil DNA/RNA extraction, library construction, and metagenomic/metatranscriptomic sequencing was provided in the Supplementary methods. Functional gene (ARGs, CMGs, and CAZyme genes) and BGC annotations followed the same procedures described above. For metatranscriptomic data, five housekeeping genes (*gyrA, gyrB, rpoA, rpoB*, and *recA*) [63] were annotated against Swiss-Prot [64] using BLASTx of DIAMOND (identity ≥80%, alignment length ≥ 25 aa, e-value ≤1e-5). Transcript abundance was normalized as RPKM = [(reads mapped to gene)/(gene length in kb)/(total millions of mRNA reads)].

### Identification and characterization of viral contigs from metagenomic and metatranscriptomic datasets

Viral contigs were identified from co-assembled contigs (>5000 bp) using geNomad v1.8.1 [65], VirSorter2 v2.2.3 [66], and CheckV v1.0.3 [67]. Contigs classified as pure virus or provirus by both geNomad and VirSorter2 were retained, followed by quality control with CheckV to trim potential host regions. Viral contigs were finalized based on virus/host gene counts, virus scores, hallmark gene counts, and length (>5000 bp after trimming), applying empirical screening criteria for soil viruses [68] to minimize false positives (detailed in the Supplementary methods). The identified viral contigs were then compiled and clustered using cd-hit-est [69] (-c 0.95 -aS 0.85 [70]) to generate viral operational taxonomic units (vOTUs). Taxonomic classification of vOTUs was assigned using geNomad based on the International Committee on Taxonomy of Viruses (ICTV) [71]. Viral AMGs were identified using VirSorter2 and DRAM-v [72], retaining only annotations where *potential_amg* was TRUE and lacking a *T* flag. Host prediction was performed with iPHoP [73], retaining virus-host pairs with confidence scores >0.9. Clean reads from metagenomic and metatranscriptomic data were mapped to vOTUs using CoverM (-p bwa-mem --min-read-percent-identity 95 --min-read-aligned-percent 90 --min-covered-fraction 70), and relative abundance and transcript abundance were normalized to RPKM [74]. Relative abundance and transcript abundance of AMGs were calculated similarly using CoverM. Total vOTU/AMG abundance per sample was calculated as the sum of the RPKM of vOTU/AMG in that sample [75].

### Statistical analysis

Given that the continental-scale sampling involved 47 sites, the 3 replicate samples taken at each site were not entirely independent. Therefore, LMMs were used to assess the effects of grouping or experimental treatments on the response variable using the R package *lme4* v1.1.36. In the group comparisons between LMW and HMW soils, the groups (LMW or HMW) were treated as fixed effects, and the 47 sites were modeled as random intercepts in the LMMs, with the detailed test model: y ≈ group + (1 | site). Similarly, in microcosm experiments, treatment and time were treated as fixed effects, while sample grouping was considered as random intercepts in the LMMs, with the detailed test model: y ≈ treatment + time + (1 | group). The standardized regression coefficients (*β*) from the LMMs reflected the effect sizes of the group or treatment. The *P* values were calculated using the R package *car* v3.1.3 and *lmerTest* v3.2.1.

The map of sampling points was made by ArcMap v10.7. The alpha diversity of microbial communities was calculated by the KrakenTools v1.2 [76]. Beta diversity of microbial communities and functional genes was calculated by R packages *vegan* v2.6-10 and *dplyr* v1.1.4. The R package *linkET* v0.0.7.4 was used for the Mantel test. Spearman’s rank correlation analyses were performed with the R packages *ggcorrplot* v0.1.4.1 and *corrplot* v0.95. Linear regression analyses and plotting were performed by the R packages *ggpubr* v0.6.0 and *ggpmisc* v0.6.1. Partial least square-structural equation modeling (PLS-SEM) was constructed by the R package *plspm* v0.5.1. In PLS-SEM, climate [mean annual temperature (MAT), annual precipitation (AP); WorldClim v2.1], soil (pH, TOC, TN), DOM traits (HMW, Chao1, MLB_R_), microbial diversity (Shannon, PC1), and carbon metabolism (CMGs, CAZyme) variables were included in the analyses of the effects of environmental variables on ARG relative abundance. Details were provided in the Supplementary methods.

## Results

### Dissolved organic matter composition and traits in high molecular weight and low molecular weight soils

**Figure 1:**
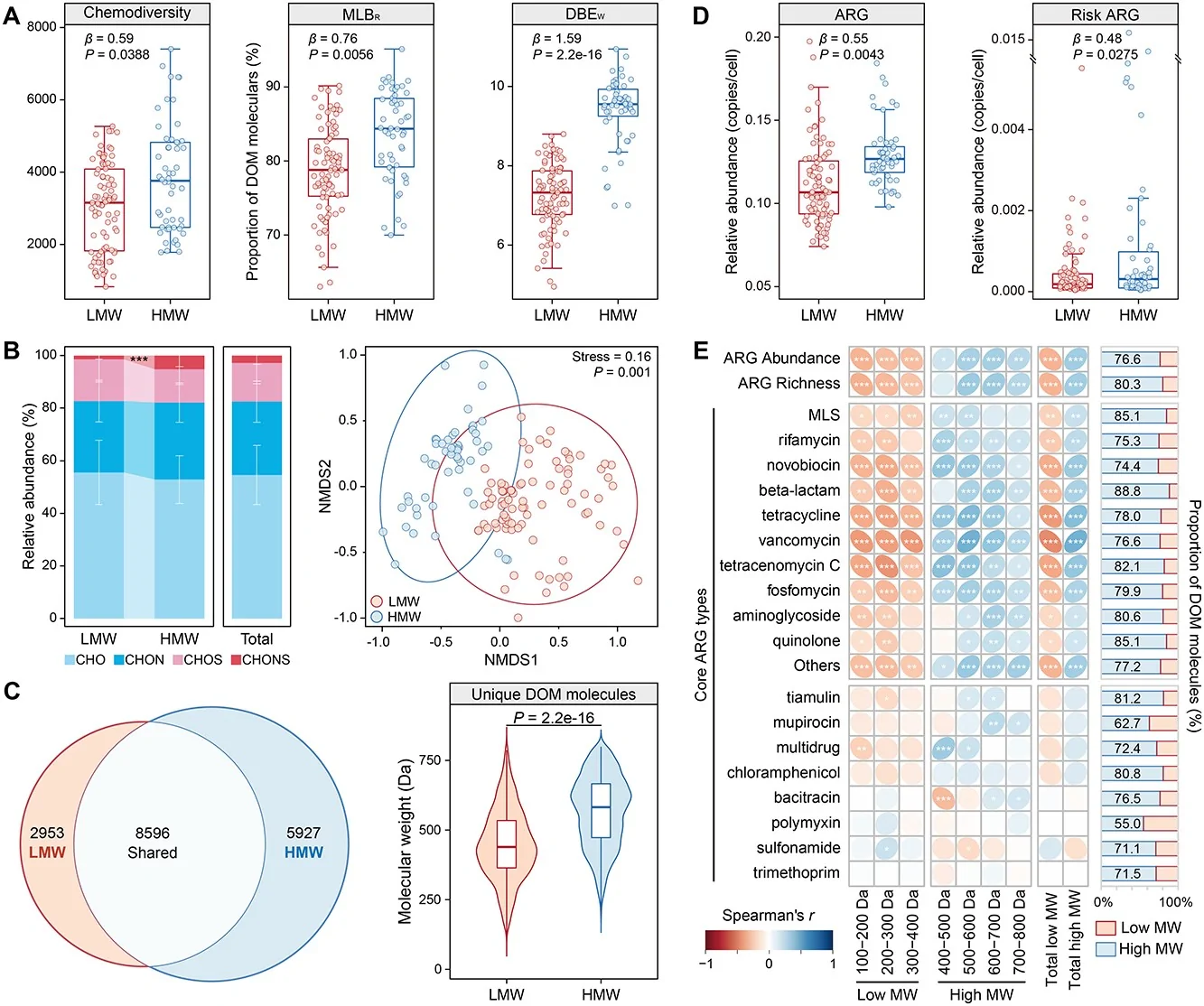
DOM molecular traits and their correlations with ARG profiles in field soils. (A) DOM molecular traits in relatively LMW (*n* = 87) and relatively HMW (*n* = 54) soils. (B) Relative abundance of DOM molecular classified by the elemental composition and nonmetric multidimensional scaling (NMDS) analysis of a Bray–Curtis dissimilarity matrix calculated from relative abundance of DOM molecules (PERMANOVA test, *F* = 19.4, *P* = .001). Each error bar corresponds to the standard deviation (SD), and data were showed as mean ± SD. (C) Number of unique and shared DOM molecules and molecular weight of unique DOM molecules in LMW and HMW soils. Two-sided Wilcoxon rank sum test was used for the significance test. (D) Relative abundance of ARGs and high-risk ARGs in LMW and HMW soils. (E) Spearman’s rank correlation of the relative abundance of DOM molecules of different molecular weights with ARGs and proportion of DOM molecules significantly positively correlated with the relative abundance of ARGs (*P* < .05). The core ARG types (except Others) were present in at least two-thirds (94 out of 141) of the samples. The remaining 10 ARG types were included in Others (other peptide antibiotics, florfenicol, puromycin, streptothricin, antibacterial fatty acid, factumycin, bleomycin, defensin, fusidic acid, and bicyclomycin). In (A), (B), and (D), LMMs were used to compare differences in HMW versus LMW soils and perform significance tests. The standardized regression coefficient (*β*) was adopted as the effect size estimated from LMMs. Significance levels: ^*^P < .05, ^**^P < .01, ^***^P < .001. MLB: molecular lability boundary, DBE: double bond equivalents, MLS: macrolide-lincosamide-streptogramin, MW: molecular weight.

DOM molecular traits, including the chemodiversity (Chao1 index), MLB_R_, and DBE_W_ were significantly higher in HMW (400 to 800 Da) soils than those in LMW (100 to 400 Da) soils (*β* = 0.59–1.59, *P* < .05; Fig. 1A). In terms of the elemental composition of DOM molecules, the dominant DOM fraction in paddy soil was CHO molecules, accounting for 54.5%, followed by CHON (27.9%), CHOS (14.7%), and CHONS (2.9%) molecules. The relative abundance of CHONS molecules was significantly higher in HMW than in LMW soils (5.2 vs*.* 1.5%; *β* = 1.12, *P* < .001; Fig. 1B). In addition, the composition of DOM molecules was significantly different between HMW and LMW soils (PERMANOVA test, *F* = 19.4, *P* = .001; Fig. 1B). About 49.2% of the DOM molecules were shared by the HMW and LMW soils, and 33.9% and 16.9% of them were uniquely identified in HMW and LMW soils, respectively (Fig. 1C). The DOM molecules specific to the HMW soils have greater MW and stability (more difficult to utilize, H/C < 1.5) than those in the LMW soils (*P* < .001; Figs 1C and S4A).

### Enrichment of antibiotic resistance genes and carbon metabolism genes in high molecular weight soils

HMW soils exhibited significantly greater ARG relative abundance (0.13 vs. 0.11 copies/cell) and diversity (Richness index: 135 vs. 116) than LMW soils (*β* = 0.48–0.79, *P* < .05; Figs 1D and S5A), with distinct ARG compositions between the two groups (PERMANOVA test, *F* = 6.9, *P* = .001; Fig. S4B). Among 18 core ARG types, 8 were significantly enriched in HMW soils, including novobiocin, MLS, tetracycline, beta-lactam, aminoglycoside, quinolone, tetracenomycin C, and fosfomycin (*β* = 0.39–0.75, *P* < .05; Table S6). At the subtype level, 58 ARGs were enriched in HMW soils (*β* = 0.34–0.73, *P* < .05), while only 7 ARGs were enriched in LMW soils (Table S7). In addition, the relative abundance of high-risk ARGs was significantly higher in HMW soils than in LMW soils (*β* = 0.48, *P* < .05), with tetracycline resistance genes as the predominant type, representing 20.5% of total high-risk ARGs (*β* = 0.61, *P* < .05), followed by MLS and multidrug resistance genes (Figs 1D and S5B). Moreover, ~2.4% of the ARG hosts were identified as potential pathogens, mostly belonging to *Pseudomonadota* and *Actinomycetota* (Fig. S6A). Among these, *Actinomycetota* was significantly enriched as ARG-carrying pathogens in the HMW soils (*β* = 0.59, *P* < .05), with the genera *Actinomyces, Actinomadura, Nocardia*, and *Tsukamurella* showing consistent enrichment. At the species level, specific human pathogens were further identified, including clinically important taxa such as *Acinetobacter baumannii, Nocardia asteroides*, and *Staphylococcus epidermidis* (Fig. S6B). Overall, relative abundance and diversity of ARGs positively correlated with HMW DOM abundance, but negatively correlated with LMW DOM abundance (Fig. 1E). Specifically, 76.5% of DOM molecules positively correlated with ARGs belonged to the HMW fraction (Fig. 1E; detailed in the Supplementary results).

**Figure 2:**
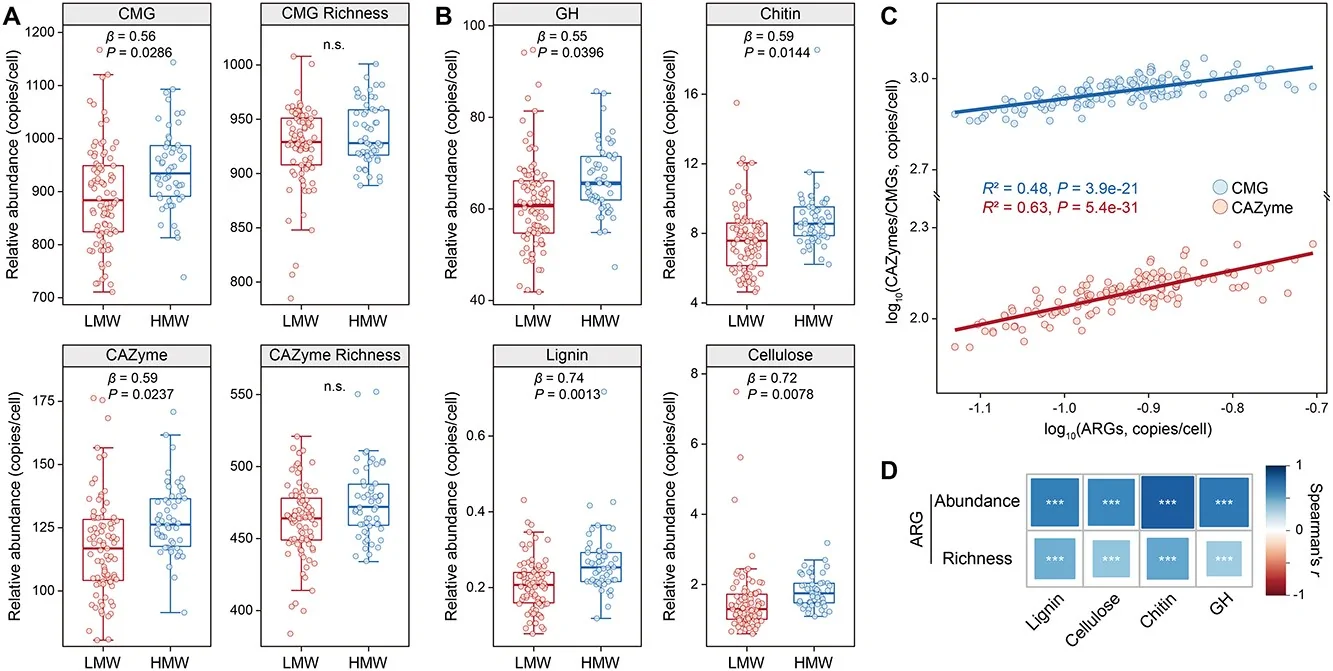
Abundance and diversity of CMGs and CAZymes genes and their associations with ARGs in field soils. (A) Relative abundances and diversity (Richness index) of CMGs and CAZyme genes in LMW and HMW soils. (B) Relative abundance of CAZyme genes that degrade complex organic matter in LMW and HMW soils. In (A) and (B), LMMs were used to compare differences in HMW versus LMW soils and perform significance tests. The standardized regression coefficient (*β*) was adopted as the effect size estimated from LMMs. n.s.: no significance. (C) Linear regression analysis of relative abundances of ARGs and CMGs/CAZyme genes. (D) Spearman’s rank correlation between the relative abundance and diversity of ARGs and the relative abundance of CAZyme genes that degrade complex organic matter. The significant differences were labeled with ^*^ (*P* < .05), ^**^ (*P* < .01), and ^***^ (*P* < .001). GH: glycoside hydrolases.

CMGs and CAZyme genes were significantly more abundant in HMW than LMW soils (*β* = 0.56–0.59, *P* < .05). But the richness of CMGs and CAZyme genes showed no differences between the two groups (Fig. 2A). Specifically, CAZyme genes involved in degrading complex organic matter, including polysaccharides (GH), chitin, lignin, and cellulose, were significantly enriched in HMW soils (*β* = 0.55–0.74, *P* < .05; Fig. 2B). The overall relative abundances of ARGs and CMGs/CAZyme genes were significantly positively correlated (*R*^2^ = 0.48–0.63, *P* < .001; Fig. 2C). Moreover, the relative abundance of CAZyme genes responsible for degrading complex organic matter was significantly positively correlated with both relative abundance and diversity of ARGs (richness) (*P* < .001; Fig. 2D).

### Contrasting drivers of antibiotic resistance genes between high molecular weight and low molecular weight soils

**Figure 3:**
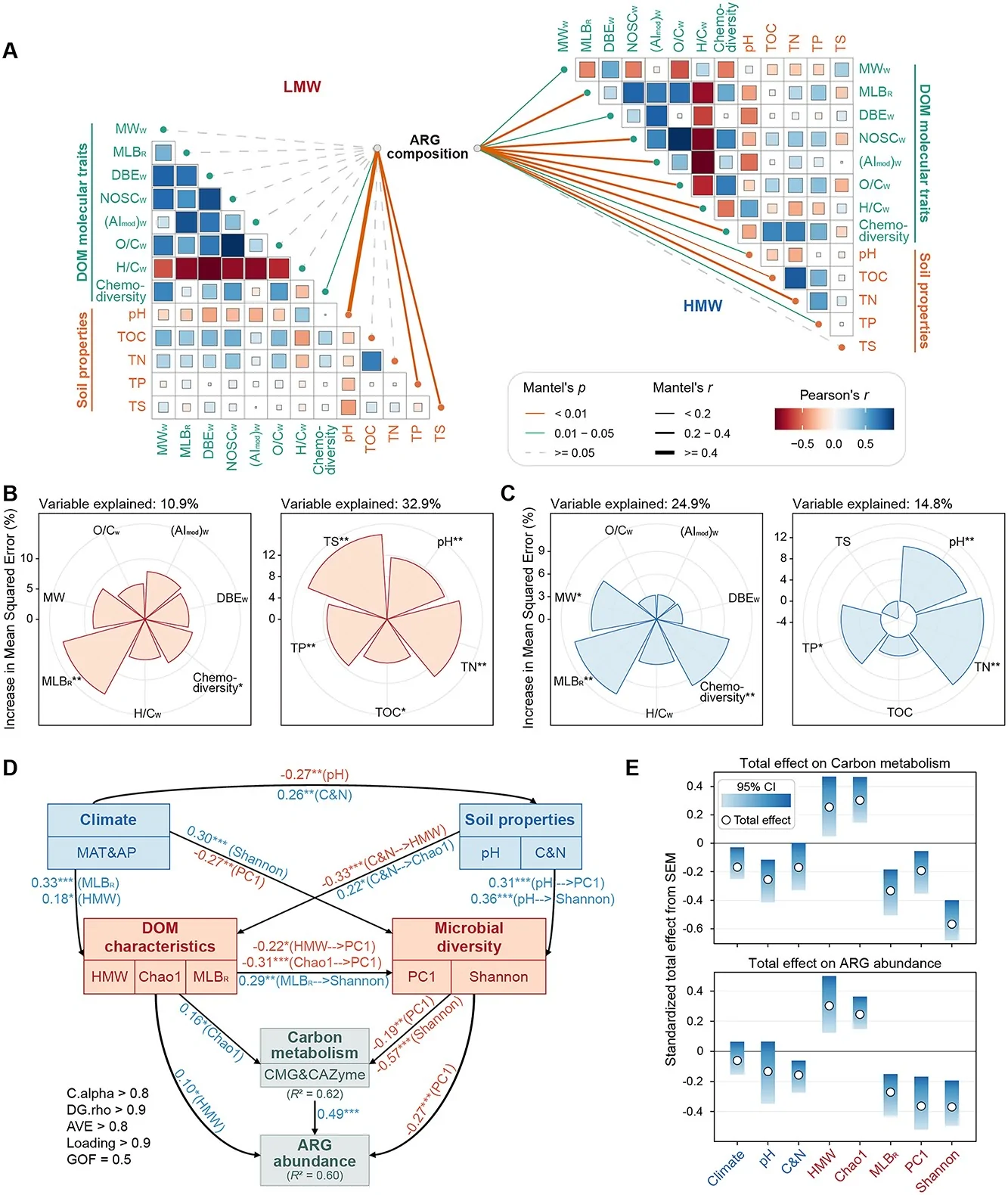
Differential drivers of soil resistomes in LMW and HMW soils. (A) The effect of soil properties and DOM molecular traits on the composition of ARGs was determined by the Mantel test in LWM and HMW soils. Mean predictor importance of soil properties and DOM molecular traits on the relative abundance of ARGs based on random forest modeling (Permutation test, *n* = 1000, ^*^P < .05; ^**^P < .01) in LMW (B) and HMW (C) soils. (D) PLS-SEM illustrated the direct and indirect effects on the relative abundance of ARGs. Path coefficients and coefficients of determination (*R*^2^) were calculated after bootstrapping (*n* = 100), and all path coefficients shown were statistically significant (^*^P < .05; ^**^P < .01, ^***^P < .001). (E) Standardized total effects from the PLS-SEM on relative abundances of carbon metabolism genes and ARGs. NOSC: nominal oxidation state of carbon, AI_mod_: modified aromaticity index, O/C: oxygen-carbon ratio, H/C: hydrogen-carbon ratio, TOC: total organic carbon, TN: total nitrogen, TP: total phosphorus, TS: total sulfur, MAT: mean annual temperature, AP: annual precipitation, C.alpha: Cronbach’s alpha, DG. Rho: Dillon-Goldstein’s rho, AVE: average variance extracted, GOF: goodness of fit, CI: confidence interval.

In HMW soils, DOM molecular traits, including MW_W_, MLB_R_, DBE_W_, NOSC_W_, (AI_mod_)_W_, O/C_W_, H/C_W_, and chemodiversity, significantly contributed to ARG composition variance (Mantel’s *r* = 0.14–0.37, *P* < .05), whereas soil properties were the primary factors correlating with ARG composition in LMW soils (Fig. 3A). Partial RDA further indicates that, after controlling for the effects of soil (pH, TOC, TN, TP, and TS) and climatic (MAT and AP) variables, the independent effect of DOM MW_W_ on ARG composition remains highly significant (Permutation test; *F* = 3.2, *P* = .002; Table S8). Random forest (RF) analysis confirmed that DOM molecular traits explained a greater proportion of relative abundance variation of ARGs in HMW than LMW soils (24.9% vs. 10.9%), while soil properties (pH, TOC, TN, TP, and TS) explained more variation in LMW soils (32.9% vs. 14.8%; Fig. 3B and C). To account for potential co-selection by metals, DOM molecular traits, soil properties, and metal concentrations (including As, Cd, Cr, Pb, and Cu) were jointly evaluated. In HMW soil, metal concentrations had no significant effect on the ARG composition (Fig. S7). The all-predictor RF model explained 54% of the variation in ARG relative abundance (Fig. S8A). DOM MW_W_ was ranked as the most important predictor, whereas metal concentrations (As, Cu, Cr) were also significant but lower-ranked. In covariate-adjusted models, relative abundance of HMW DOM remained positively associated with relative abundance of ARG (*β* = 0.246, 95% CI = 0.009–0.483, *P* < .05) and richness (*β* = 0.493, 95% CI = 0.219–0.768, *P* < .001), whereas the corresponding conditional linear effects of As concentration were not significant (Fig. S8B and C). PLS-SEM had a goodness of fit of 0.5 and explained 60% of the variance in relative abundance of ARGs. It revealed that relative abundance of HMW DOM and CMGs/CAZyme gene had direct positive effects on relative abundance of ARGs (path coefficient of 0.10 and 0.49, *P* < .05), whereas microbial beta-diversity had a direct negative effect (path coefficient = −0.27, *P* < .001; Fig. 3D). Moreover, DOM chemodiversity (Chao1) indirectly promoted relative abundance of ARGs by positively influencing CMGs/CAZyme gene abundance (path coefficient = 0.16, *P* < .05), which in turn was associated with ARG enrichment. In addition, the suppressive effects of HMW DOM abundance and DOM chemodiversity on microbial diversity (path coefficient of −0.31 and −0.22, *P* < .05), together with the suppressive effect of microbial diversity on carbon metabolism, constitute a double-negative pathway that indirectly enhances relative abundance enrichment of ARGs in soils. Furthermore, climate factors and soil properties (pH, TOC, and TN) influenced relative abundance of ARGs indirectly through DOM characteristics and microbial diversity (Fig. 3D). Overall, HMW DOM abundance exerted the strongest positive total effect on ARGs (0.30), followed by DOM chemodiversity (0.24), whereas microbial diversity was the primary negative factor (Fig. 3E).

### High molecular weight-enriched metagenome-assembled genomes co-carry antibiotic resistance genes, carbohydrate-active enzymes, and antimicrobial biosynthetic gene clusters in soils

**Figure 4:**
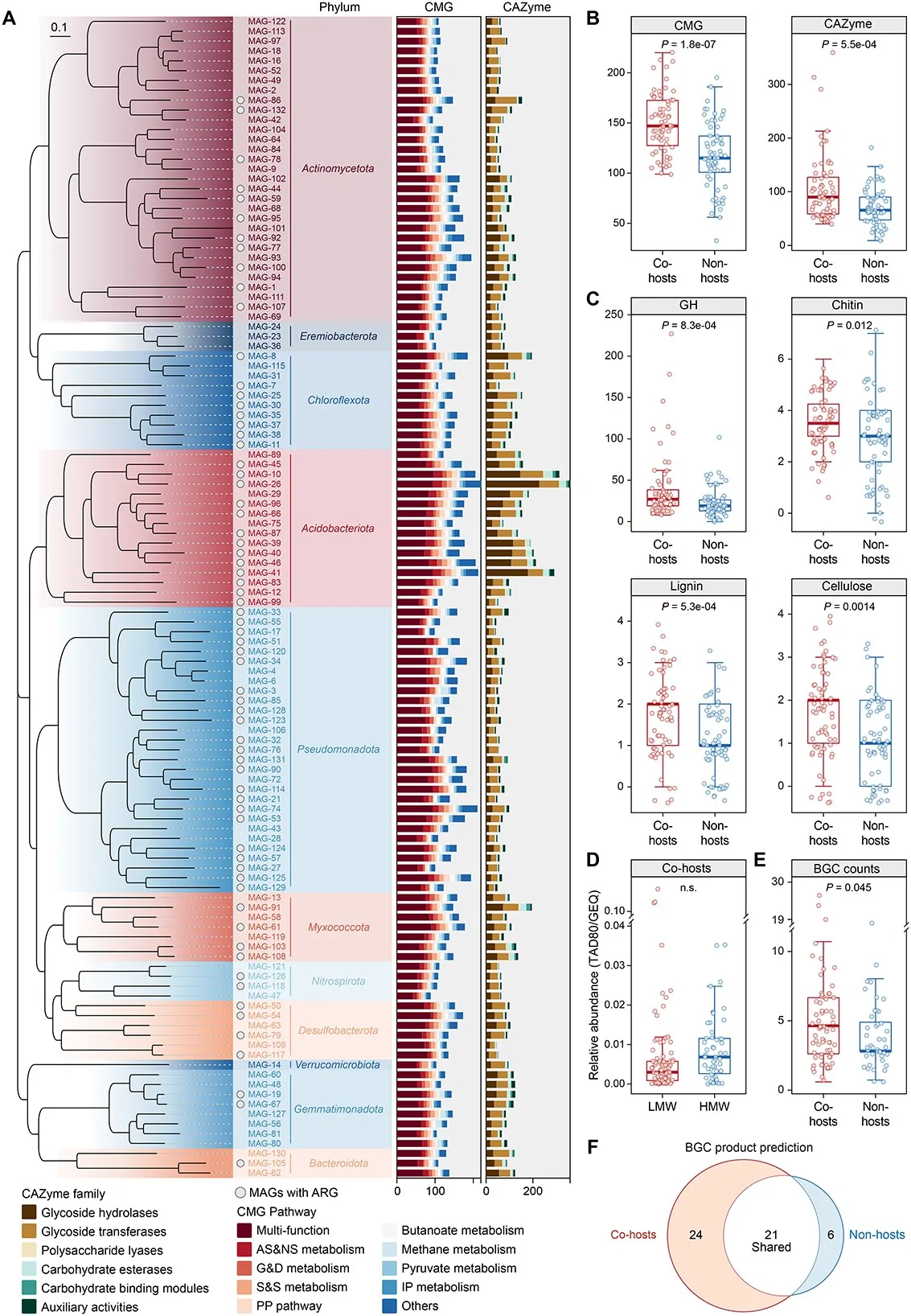
Phylogenomic analysis of MAGs and genomic characterization of co-hosts versus non-hosts in field soils. (A) Bacterial phylogenetic tree of high-quality (completeness ≥90% and contamination ≤5%) MAGs. The colors of MAGs represented the different Phylum levels to which these MAGs were assigned. The number of identified CMGs and CAZyme genes among the MAGs was shown in the bar plots. Multifunction indicated genes present in more than one carbon metabolic pathway. (B) The number of CMGs and CAZyme genes in co-hosts (*n* = 68) and non-hosts (*n* = 64). (C) The number of GHs enzymes, chitin enzymes, lignin enzymes, and cellulose enzymes in co-hosts and non-hosts, respectively. (D) A comparison of the relative abundances of co-hosts in HMW and LMW soils based on the LMM. (E) BGC counts in co-hosts and non-hosts. (F) Number of shared and unique predicted natural products synthesized by BGCs in co-hosts and non-hosts. In (B), (C), and (E), two-sided Wilcoxon rank sum test was used for all significance tests. AS&NS: amino sugar and nucleotide sugar, G&D: glyoxylate and dicarboxylate, S&S: starch and sucrose, PP: pentose phosphate, IP: inositol phosphate.

A total of 132 high-quality nonredundant MAGs (completeness ≥90% and contamination ≤5%) were recovered from the soils, of which 51.5% (68/132) carried at least one ARG (Figs 4A and S9A). These 68 MAGs, designated as co-hosts, also harbored CMGs and CAZyme genes, and were primarily affiliated with *Pseudomonadota* (23), *Acidobacteriota* (13), *Actinomycetota* (11), and *Chloroflexota* (8; Fig. 4A). The total relative abundance of co-hosts was higher in HMW than in LMW soils (0.0068 vs. 0.0033 TAD80/GEQ; Fig. 4D). Co-hosts contained significantly more CMGs and CAZyme genes than non-hosts (147 vs. 115 and 91 vs. 66, respectively; *P* < .001; Fig. 4B), with no difference in genome size (*P* > .05; Fig. S9B). Specifically, co-hosts possessed a greater number of enzymes (GH, chitin, lignin, and cellulose) involved in degrading complex organic matter than non-hosts (*P* < .05; Fig. 4C).

Analysis of BGCs revealed 562 BGCs encoding 7 natural products in MAGs (Table S9). Co-hosts harbored a greater number of BGCs for RiPPs, NRPS, and other PKS than non-hosts (*P* < .05; Figs 4E and S10A). Furthermore, co-hosts were capable of producing a greater diversity of unique natural products (24 vs. 6), including antimicrobial compounds, such as lasso peptide, Class V lanthipeptide, NRP-metallophore, and indole (Fig. 4F and Table S10). Relative abundance of BGCs also showed an increasing trend in HMW compared to LMW soils, and 38% of co-host MAGs (26/68) showed coordinated increases in the relative abundance of MAGs, ARGs, and BGCs from LMW to HMW soils (Figs S10B and S11). These MAGs were predominantly *Pseudomonadota*, followed by *Acidobacteriota, Nitrospirota, Myxococcota, Gemmatimonadota, Chloroflexota, Actinomycetota, Desulfobacterota*, and *Bacteroidota*. These MAGs primarily carried multidrug, followed by polymyxin, bacitracin, novobiocin, and rifamycin resistance genes, with BGCs primarily associated with RiPPs, Terpene, NRPS, and Others (Fig. S11).

### Effects of dissolved organic matter addition on antibiotic resistance genes, carbon metabolism genes, and co-host metagenome-assembled genomes in high molecular weight versus low molecular weight soils

**Figure 5:**
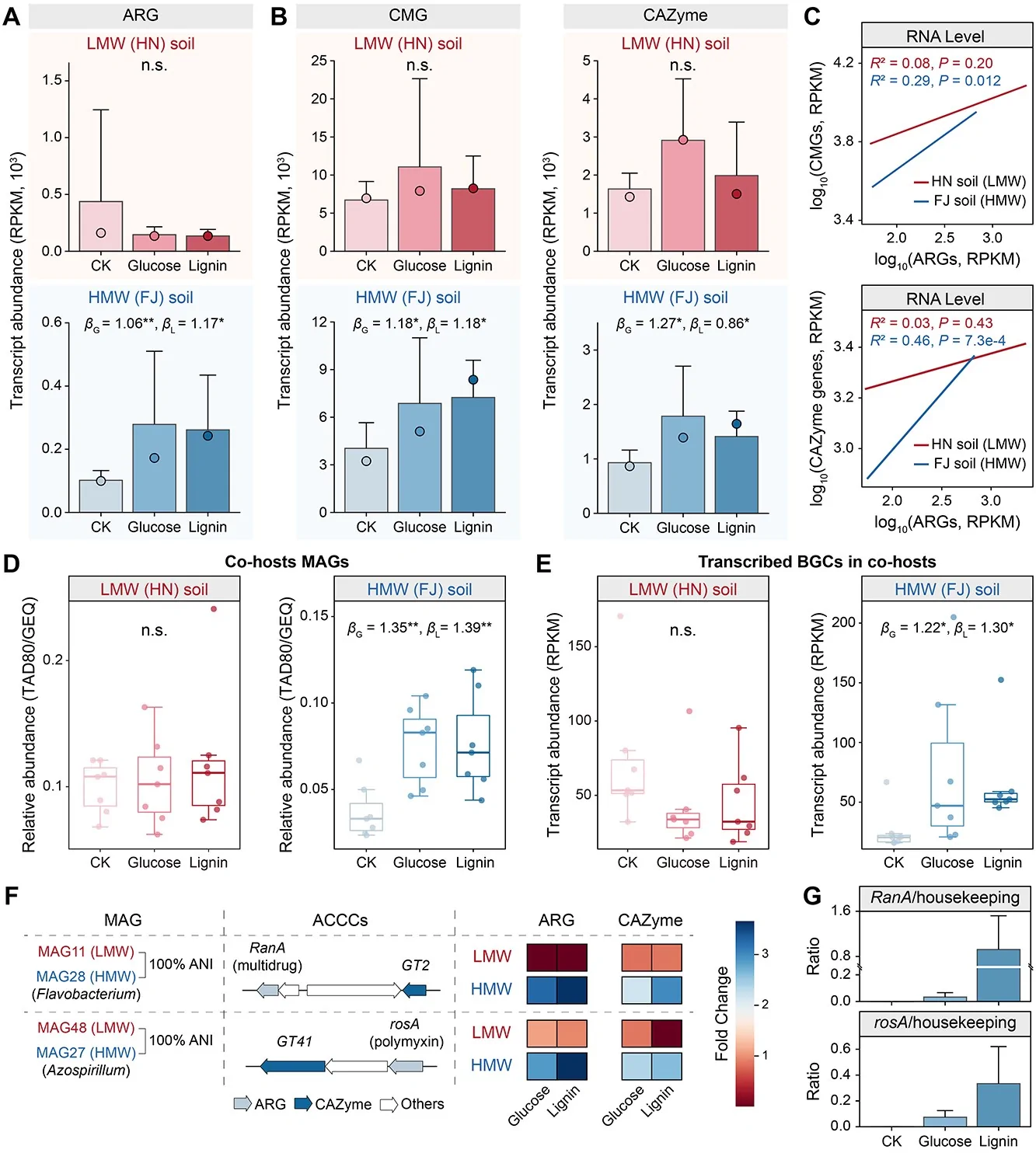
Transcriptional responses of functional genes to DOM additions in microcosm experiments. Relative abundance of transcribed ARGs (A), CMGs, and CAZyme genes (B) in different treatments (*n* = 7). Each error bar corresponds to the SD, and data were showed as mean + SD. The circles represented median values. (C) Linear regression analysis of transcript abundances of ARGs and CMGs/CAZyme genes in LMW (HN) and HMW (FJ) soils. (D) Relative abundance of co-hosts in different treatments in LMW and HMW soils. (E) Relative abundance of transcribed BGCs in different treatments in LMW and HMW soils. In (A), (B), (D), and (E), LMMs were used to compare differences in treatments (glucose and lignin) versus control and perform significance tests. The standardized regression coefficient (*β*) was adopted as the effect size estimated from LMMs. *β_G_*: Effect size of glucose treatment versus control, *β_L_*: effect size of lignin treatment versus control. (F) Representative arrangements of ACCCs in MAGs. The heatmap illustrates the fold change in abundance of transcribed ARG and CAZyme in MAGs compared to the control treatment. (G) The transcriptional ratio of ARGs to housekeeping genes in MAGs from Fig. 5F under different treatments in HMW soil.

In microcosm experiments, addition of HMW (lignin) and LMW (glucose) DOM to HMW soil significantly increased the relative abundance of ARGs at both DNA and RNA levels compared to the control (*β*_G_ = 1.06–1.60, *β*_L_ = 1.12–1.17, *P* < .05; Figs 5A and S12A). A concurrent increase in the ARG-to-housekeeping gene transcript ratio was also observed in HMW versus LMW soil after DOM addition (*β*_G_ = 1.05, *β*_L_ = 0.71, *P* < .05; Fig. S13). Similarly, the relative abundances of CMGs, CAZyme genes, transcribed CMGs, and transcribed CAZyme genes were significantly elevated in HMW soil following DOM addition (*β*_G_ = 0.95–1.43, *β*_L_ = 0.54–1.18, *P* < .05; Figs 5B and S12B). In contrast, for LMW soil, DOM addition only increased relative abundance of ARGs at the DNA level (*β*_G_ = 0.92, *β*_L_ = 1.10, *P* < .05), with no significant changes in ARGs, CMGs, or CAZyme genes at either DNA or RNA level (Figs 5A, B, and  S12). Furthermore, a significant positive correlation between relative abundances of transcribed ARGs and CMGs/CAZyme genes was observed only in HMW (*R*^2^ = 0.29–0.46, *P* < .05; Fig. 5C).

Among high-quality MAGs (completeness ≥90%, contamination ≤5%) recovered from HMW and LMW soils, 33 and 110 co-hosts were identified, respectively (Tables S11 and S12). In HMW soil, DOM addition significantly increased the relative abundance of these co-hosts (*β*_G_ = 1.35, *β*_L_ = 1.39, *P* < .05; Fig. 5D) and their BGCs at both DNA and RNA levels (*β*_G_ = 1.22–1.27, *β*_L_ = 1.30–1.40, *P* < .05). Yet in LMW soil, no such effects were observed (Figs 5E and S14). In addition, a greater number of CMGs and CAZyme genes (including GH enzyme genes and lignin enzyme genes) were detected in co-hosts compared to non-hosts in HMW soil (*P* < .01; Table S13). Specifically, MAG 11 and MAG 48 from LMW soil showed 100% ANI with MAG 28 and MAG 27 from HMW soil, respectively (Fig. S15). On their ARG-CAZyme-carrying contigs (ACCCs), transcriptional levels of co-located *RanA* (multidrug resistance) with GT2 (glycosyl transferase family), and *rosA* (polymyxin resistance) with GT41 (glycosyl transferase family) were higher in HMW than in LMW soil after DOM addition, with lignin amendment showing the greatest effect (Fig. 5F). Genomic analysis revealed that these ARG-carrying MAGs also co-harbored CAZymes for complex DOM degradation and BGCs for secondary metabolite biosynthesis (Fig. S16). Consistently, transcript ratios of *RanA* and *rosA* (normalized to housekeeping genes) in HMW soil were highest in the lignin treatment, followed by the glucose and control treatments (Fig. 5G).

### Effects of dissolved organic matter additions on viral and auxiliary metabolic gene abundance in high molecular weight versus low molecular weight soils

**Figure 6:**
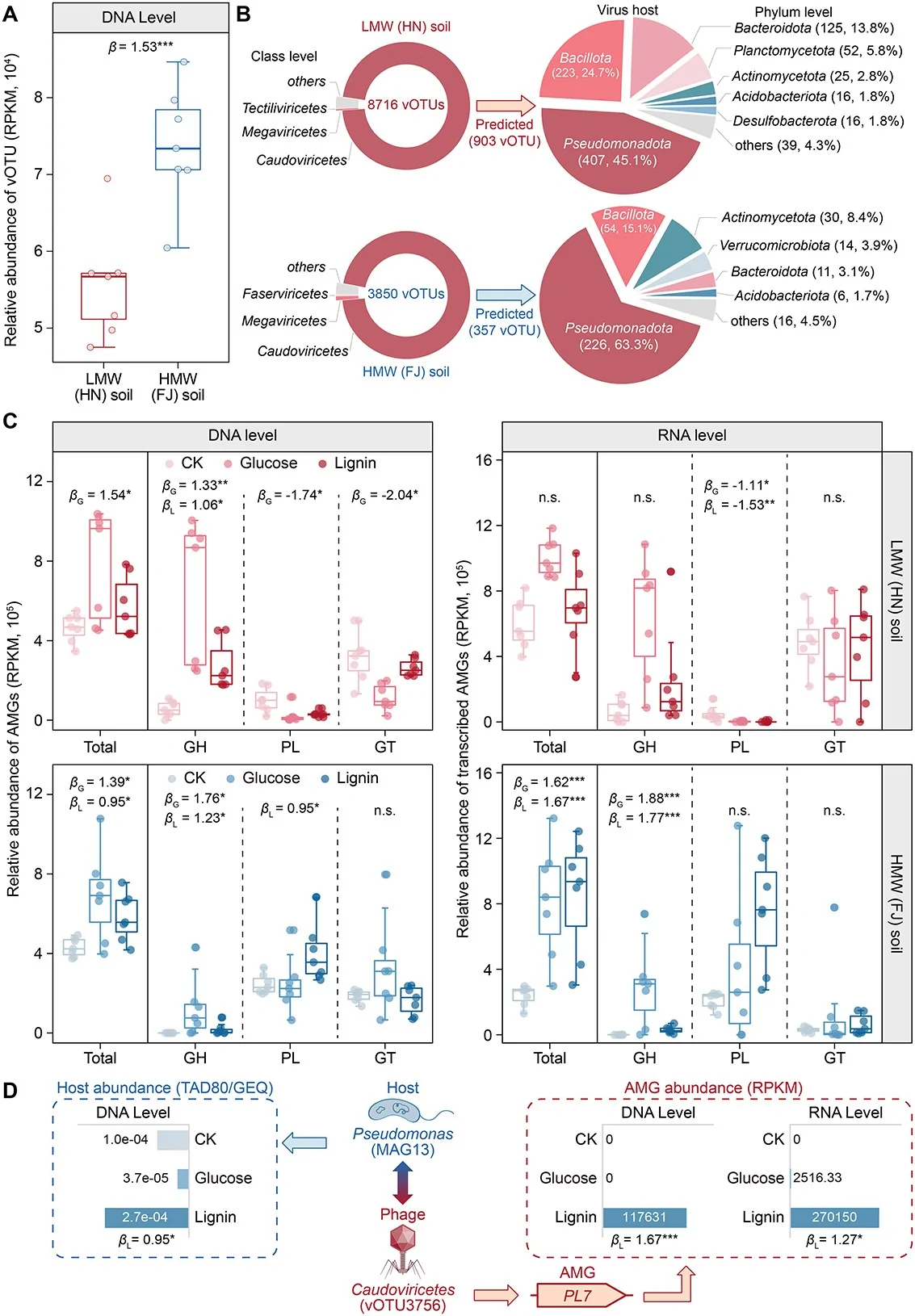
Viral composition, host prediction, and their carbon-degrading AMG responses to DOM additions in microcosm experiments. (A) Relative abundance of viruses in LMW and HMW soils before DOM addition (CK, *n* = 8). (B) Taxonomic classification and host prediction of viruses in LMW and HMW soils. (C) Relative abundance and transcript abundance of AMGs annotated as CAZyme genes at the family level. (D) A typical example of the phage assisting host metabolism in HMW soil. The bar plots represent the abundance by median values of host and AMG in different treatments. In (A), (C), and (D), LMMs were used to compare differences in treatments (glucose and lignin) versus control and perform significance tests. The standardized regression coefficient (*β*) was adopted as the effect size estimated from LMMs.

The total relative abundance of viruses in HMW (FJ) soil was 1.3 times higher than in LMW (HN) soil before the DOM additions (*β* = 1.53, *P* < .001; Fig. 6A). The annotated viruses were mainly assigned to *Caudoviricetes* and *Megaviricetes* at the class level in HMW and LMW soils (Tables S14 and S15). *Pseudomonadota* were the most frequently predicted viral hosts, followed by *Bacillota*, accounting for 63.3% and 45.1%, 15.1% and 24.7% in HMW and LMW soils of the total identified viral hosts, respectively (Fig. 6B). In HMW soil, the total relative abundance of AMGs significantly increased with either lignin or glucose addition at DNA and RNA level than the control treatment (*β*_G_ = 1.39–1.62, *β*_L_ = 0.95–1.67, *P* < .05). However, in LMW soil, the significant increase of AMGs was observed after glucose addition compared to the control treatment at the DNA level (*β*_G_ = 1.54, *P* < .05; Fig. 6C). Among the AMGs, the GH family genes, which are responsible for the degradation of complex carbohydrates, showed a significant increase in their relative abundance at both the DNA and RNA levels in HMW soils with DOM addition compared to the control treatment (*β*_G_ = 1.76–1.88, *β*_L_ = 1.23–1.77, *P* < .05). Whereas this trend was not observed for the LMW soils at RNA level (detailed in the Supplementary results). Moreover, we observed that only lignin addition increased the relative abundance of AMGs annotated as Polysaccharide Lyase (PL) families at both the DNA and RNA levels in HMW soils, reflecting a striking lignin-specific response (Fig. 6C).

A paradigmatic example of this lignin-specific mutualism was identified in HMW soil (Fig. 6D). The vOTU3756 (*Caudoviricetes*), which encodes a *PL7* gene for complex polysaccharide degradation, was exclusively enriched after lignin addition at both DNA (*β*_L_ = 1.67, *P* < .001) and RNA levels (*β*_L_ = 1.27, *P* < .05). Consistently, its predicted host MAG 13 (*Pseudomonas*) was also significantly enriched only in the lignin treatment (*β*_L_ = 0.95, *P* < .05; Fig. 6D). However, no such enrichment was observed in the glucose treatment. In addition, no ARGs were observed in these identified vOTUs either in HMW or LMW soils with different MW DOM addition. Due to the relatively low sequencing depth of the metagenomic datasets from field soils, we were unable to obtain sufficient viral sequences for viral analysis. Although a total of 4060 vOTUs were identified from the field metagenomic datasets using a co-assembly approach, only 0–327 vOTUs exhibited detectable abundance (>0) across different samples. Thus, the AMGs within the vOTUs were further analyzed and revealed a relatively higher abundance of GH family genes (*GH16* and *GH43*) in HMW soils compared to LMW soils, although this difference did not reach statistical significance (*β* = 0.34, *P* = .17; Fig. S17).

## Discussion

The emergence of ARGs as environmental contaminants in soils was confirmed to be driven by ecological drivers during intrinsic evolutionary processes [2]. While previous studies have revealed the significantly negative correlation of TOC with relative abundance of ARGs and diversity [13, 14], our results further demonstrated that soil DOM molecular weight was the determinant of ARGs proliferation by altering microbial community dynamics (Figs 1 and 3). Specifically, the LMW DOM, which typically represents labile carbon sources that support rapid microbial growth [13, 22], showed a significantly negative correlation with relative abundance of ARGs (Fig. 1). This could be attributed to the “metabolic trade-off” hypothesis as the utilization of easily degradable substrates prioritizes microbial growth over maintaining resistance mechanisms [77]. Conversely, the relative abundance of HMW DOM molecules in soils positively correlated with relative abundance of ARGs. In addition, relative abundance and diversity (Richness) of ARGs were significantly higher in HMW than LMW soils, as confirmed by the comparison of 141 paddy soils at the continental scale (Figs 1 and S5). HMW DOM does not merely increase overall relative abundance of ARGs but selectively enriches high-risk ARGs, distinguishing them from common soil bacterial ARGs (e.g. efflux [78] or signaling functions [79]). Moreover, potentially pathogenic ARG hosts (e.g. *A. baumannii* [80], *N. asteroides* [81], and *S. epidermidis* [82]) were enriched in HMW DOM soils (Fig. S6). This selective enrichment further highlights the potential pathogenic risks associated with the ARG enrichment. The Mantel test and random forest modeling further indicated that DOM molecular traits (especially MW_W_, MLB_R_, and chemodiversity) significantly influenced relative abundance and composition of ARGs in HMW soils (Fig. 3). Whereas this effect was not observed in LMW soils. Specifically, the relative abundance of HMW DOM, together with the DOM chemodiversity, emerged as the most critical factors, which acquired a total effect of 0.3 and 0.24 on relative abundance of ARGs, respectively (Fig. 3). While metal co-selection may have a contributing role, the dominant and consistent driver of resistome enrichment in HMW soil is the molecular weight and associated traits of DOM, with microbial community composition serving as an important mediating pathway (Figs S7 and S8). Collectively, these results demonstrate that soil HMW DOM is a key driver of ARG enrichment, expanding our understanding of the factors that determine ARGs.

The low bioavailability of HMW DOM, indicated by higher DBE_W_ and H/C ratio (Figs 1, S4, and  S18) in HMW soils, likely imposes nutritional constraints on microbial communities, and thereby enhances microbial competition [3, 13]. According to the niche breadth and overlap analyses, the overall microbial niche breadth was significantly lower in HMW soils compared to LMW soils (*β* = −0.56, *P* < .041; Fig. S19), indicating that microbial communities in HMW soils occupy narrower ecological niches and thus experience more intense resource competition. This pattern aligns with the chemical nature of HMW DOM, which consists primarily of complex, recalcitrant compounds accessible only to a limited subset of microorganisms, leading to stronger niche partitioning and interspecific competition (Fig. 1). Moreover, co-hosts exhibited higher niche overlap scores than non-host MAGs (Fig. S19), suggesting that these co-hosts share more similar carbon resource requirements and thus engage in more direct competitive interactions with each other. Previous studies also demonstrate that the bioavailability of carbon sources is a critical factor limiting microbial growth and metabolism [11, 22] and a key driver shaping competitive interactions within soil microbial communities [3, 83]. Corroboratively, the higher proportion of negative correlations was observed in HMW than in LMW soils in the microbial co-occurrence network (*P* < .05; Fig. S20 and Table S16), suggesting stronger potential competition between microbial communities. In addition, the core genetic basis of microbial competition, especially BGCs, which produce chemical weapons that directly interfere with resource competition [60, 84, 85], was actively expressed in the microcosm experiments after DOM addition in HMW (FJ) soil (Fig. 5). The co-hosts carrying both ARGs, CMGs, and CAZyme genes were also identified with more abundant and diverse categories of BGCs (Fig. 4 and Table S9), especially those encoding unique antimicrobial compounds (such as indole [86, 87] and lasso peptides [88]) in their genomes (Table S10). Furthermore, both the relative abundance of these co-hosts as well as the transcribed BGCs in their genomes significantly increased in the HMW soils after DOM addition (Fig. 5), corroborating the potential microbial competition in HMW soils. *Actinomycetes* (*Actinomycetota*), which has been reported as a typical antibiotic producer [79, 89], were enriched in HMW soil (Figs S6 and S21), further confirming that the co-hosts could acquire the competitive advantage by expressing BGCs that produce antibiotics in HMW soils.

In response, ARGs, as an evolutionary consequence of chemical warfare, were significantly expressed after DOM addition in HMW soils (Figs 5 and S12), probably exist as a self-defense mechanism for microorganisms [90, 91]. In addition, the enrichment of ARGs and CMGs/CAZyme genes is closely associated, especially in HMW soil, as shown by the significant positive correlation between their abundances (Figs 1, 2, and  5). Moreover, the proliferation of co-hosts carrying ARGs and CMGs/CAZyme genes in HMW soils was confirmed in the HMW soils both at the continental scale and the incubation experiments (Figs 4 and 5). The ARGs and diverse CMGs/CAZyme genes carried by co-hosts can enhance their competitiveness by improving their ability to utilize HMW DOM with low bioavailability, while simultaneously strengthening their resistance to antimicrobial substances. Consistently, the co-expression of ARGs and CAZyme genes on ACCCs in co-hosts in HMW (FJ) soil was detected, especially after HMW DOM (lignin) addition (Fig. 5). Similar results were found in soils with high nitrogen levels, where changes in ARGs depended on copiotrophs with nitrogen metabolic capacities [92]. Herein, we further observed that co-hosts harbored a greater number of CAZyme genes than the non-hosts, particularly those encoding enzymes for the decomposition of complex carbon substrates such as polysaccharides and lignin (Fig. 4 and Table S13) in HMW soils. The HMW DOM degradation capabilities of these co-hosts to degrade HMW DOM via secreted extracellular enzymes [93] likely enhanced their competitive fitness for resources, ultimately promoting their enrichment under HMW DOM supplementation. Whereas recent evidence confirms that carbon metabolism plays a pivotal role in the evolution of antibiotic resistance [94–96], we further clarify that the HMW DOM, as a carbon source, amplifies soil resistome development through substrate-driven microbial selection, which modulates microbial community dynamics and promotes the expansion of ARGs. This could represent a potential yet overlooked ecological driver of ARG proliferation, alongside well-established factors such as residual antibiotics or metals from organic fertilization [9].

Previous studies reported that viruses in activated sludge [97], composting systems [98], and aquaculture environments [99] could facilitate the spread of ARGs between hosts. However, rather than contributing to the ARG accumulation, phages in the HMW DOM soils mostly enhanced host competitiveness and adaptation by AMGs that assisted carbon degradation (Fig. 6) [3]. Specifically, the relative abundance of viruses was significantly greater in HMW than LMW soils (Fig. 6). Moreover, the relative abundance of total AMGs and transcribed AMGs annotated as CAZyme genes significantly increased in HMW (FJ) soil after the DOM addition (Fig. 6). In addition, a typical example of a mutually beneficial symbiosis between a phage and its host was discovered in HMW soil. In particular, the enrichment of the AMG annotated to the *PL7* gene that responsible for lignin degradation was observed in *Caudoviricetes*, possibly enhancing its host (MAG13: *Pseudomonadota*) competitive fitness for resources after lignin (HMW DOM) addition (Fig. 6). The direct contribution of viruses to the horizontal transfer of ARGs was not identified in our experimental system. However, due to sequencing depth limitations, our viral analysis may have underestimated soil viral diversity, potentially missing low-abundance ARG-carrying viruses. Future viral-enriched metagenomics with deeper sequencing are needed to fully assess their role in ARG dissemination in paddy soils.

It should be acknowledged that multiple environmental factors, including heavy metal concentrations and microbial community composition, collectively contribute to resistome development, and the relative importance of DOM molecular weight may vary under different environmental contexts. Our findings are based on field soils with distinct HMW and LMW DOM characteristics, and the microcosm experiments demonstrated that the effect of DOM addition is context-dependent and mediated by the preexisting microbial community. Therefore, while DOM molecular weight emerged as a robust predictor in our dataset, the interactive effects among DOM properties, microbial community composition, and other co-occurring selective pressures warrant further investigation across broader spatial and temporal scales to establish the generalizability of our conclusions.

In summary, our findings underscore that HMW DOM acts as a key driver of soil resistome proliferation. A microbial ecological framework is proposed encompassing three synergistic dimensions. First, at the microbial community level, the low bioavailability of HMW DOM intensifies interspecific resource competition. This is evidenced by a higher proportion of negative correlations in the co-occurrence network and a significantly lower niche breadth in HMW soils, which together select for microorganisms carrying BGCs and co-evolved ARGs. Second, at the functional gene level, HMW DOM enriches CAZyme-harboring microorganisms for complex carbon degradation. Third, phages carrying AMGs further enhance host competitiveness in HMW soils. Together, these processes drive the co-enrichment of multifunctional hosts carrying ARGs, CMGs, BGCs, and AMGs. Microcosm experiments confirmed increased ARG transcription (2.4-fold), co-host abundance (2.3-fold), and AMG transcription (2.5-fold), and PLS-SEM identified HMW DOM abundance and chemodiversity as the strongest positive drivers of relative abundance of ARGs. These findings redefine organic matter management in agricultural soils, challenging conventional practices that focus solely on carbon quantity, and highlighting the potential of molecular-level interventions to mitigate ARG spread. Nevertheless, several limitations of this study should be acknowledged. The 15-day microcosm experiment captures short-term transcriptional responses, but longer-term community succession may alter ARG dynamics over extended periods. Additionally, lignin was used as a representative HMW DOM compound even though soil HMW DOM comprises diverse complex molecules such as cellulose and humic substances, so testing additional HMW compounds will help confirm the generalizability of these findings. Furthermore, the microcosm experiment was conducted using only two representative paddy soils. The results may require further validation using a wider range of paddy soils. Future long-term incubations and field monitoring are needed to address these aspects.

## Supplementary Material

Supplementary_Material_clean_wrag212(1)

## Data Availability

The raw metagenomic (44) and metatranscriptomic (44) data generated from the microcosm experiment in this study have been deposited in the NCBI Sequence Read Archive (SRA) database under the BioProject PRJNA1293731 and PRJNA1293776, respectively. The 141 metagenomic datasets from the field study were obtained from the NCBI SRA under the BioProject accession number PRJNA1068274.
