# Supplementary material for: High molecular weight dissolved organic matter drives soil resistome proliferation by enhancing microbial competition and viral carbon metabolism
Source: ISME J. 2026 Aug 18;20(1):wrag212. doi: 10.1093/ismejo/wrag212 (PMC13615529; doi:10.1093/ismejo/wrag212)
Supplement: Supplementary file 1 [file supplementary_material_clean_wrag212(1).docx]

**Supplementary material**

**High molecular weight dissolved organic matter drives soil resistome proliferation by enhancing microbial competition and viral carbon metabolism**

Zi-Teng Liu^1^, Xin-Di Zhao^1^, Jia-Qi Li^1^, Shu-Xin Li^1^, Xianjin Tang^2^, Si-Yu Zhang^1, 3*^

1. Zhejiang Zhoushan Island Observation and Research Station, State Key Laboratory of Estuarine and Coastal Research, School of Ecological and Environmental Sciences, East China Normal University, Shanghai 200241, China

2. Zhejiang Provincial Key Laboratory of Agricultural Resources and Environment, College of Environmental and Resource Sciences, Zhejiang University, Hangzhou 310058, China

3. Institute of Eco-Chongming (IEC), East China Normal University, Shanghai 202151, China

*Corresponding author: Si-Yu Zhang, School of Ecological and Environmental Sciences, East China Normal University, Shanghai 200241, China. E-mail: [syzhang@des.ecnu.edu.cn](mailto:syzhang@des.ecnu.edu.cn)

**This PDF file includes:**

Supplementary methods

Supplementary results

Supplementary figures

Supplementary tables

**Supplementary methods**

**Analyses of soil chemical properties**

Soil pH was determined in a soil suspension (1:2.5 w/v ratio) prepared with deionized water, using a digital FE28-Standard pH meter (Mettler Toledo, Switzerland). Soil properties were assessed according to the national standards of China. Organic matter (OM) content was measured using titration methods as specified by the HJ 615-2011 guidelines. The total organic carbon (TOC) content was derived from the OM content, following the HJ 615-2011 guidelines. Total nitrogen (TN) content was determined by titration, according to the HJ 717-2014 guidelines. Total phosphorus (TP) was quantified using the molybdenum-antimony colorimetric method and a T6 UV-VIS spectrophotometer (PERSEE, China), based on the LY/T 1232-2015 standards. Total sulfur (TS) content was analyzed with a CS230 carbon sulfur analyzer (LECO, USA).

**Extraction of dissolved organic matter (DOM)**

A specific mass of soil was weighed into a centrifuge tube, with the amount determined based on the TOC or dissolved organic carbon (DOC) content of the soil. The soil was then leached with ultrapure water at a soil-to-water mass ratio of 1:10. The centrifuge tube was incubated at a constant temperature of 25 ± 1°C and agitated for 8 hours. Following this, the sample was centrifuged at 4200 rpm for 20 minutes using a high-speed centrifuge, and the supernatant was filtered through a 0.22 µm cellulose acetate membrane filter. The pH of the filtrate was adjusted to 2 with 1 mol/L hydrochloric acid. Subsequently, the filtered and acidified DOM was concentrated using an Agilent Bond Elut PPL (500 mg, 6 ml) column. Solid-phase extraction (SPE) to concentrate the DOM involved several steps: (1) the PPL column was activated by eluting with 12 ml of methanol (chromatographic grade) followed by 12 ml of 0.01 mol/L hydrochloric acid; (2) the sample was gradually loaded onto the PPL column at a flow rate of 2 ml/min until the organic carbon content reached approximately 100 mg C/L; (3) the PPL column was rinsed with 18 ml of 0.01 mol/L hydrochloric acid to remove any salts; (4) the column was dried under a stream of nitrogen gas, and the DOM was then eluted with 12 ml of methanol, with the eluate collected; and (5) the eluate was subjected to nitrogen blowdown to remove all methanol by evaporation. Prior to analysis by Fourier transform ion cyclotron resonance mass spectrometry (FT-ICR MS), the residue was re-dissolved in 1 ml of a 1:1 methanol-water solution.

**DOM preprocessing based on FT-ICR MS**

The molecular composition of DOM was analyzed using a SolariX-type FT-ICR MS (Bruker, USA) instrument equipped with an electrospray ionization (ESI) source. Detection was carried out in negative ion mode with the following key parameters: (1) samples were continuously introduced at a flow rate of 120 μL/h; (2) the capillary entrance voltage was set to -4.0 kV; (3) ion accumulation time was 0.2 s; (4) the mass range was recorded from 100 to 1600 Da; (5) data acquisition was conducted with a 4 M 32-bit data point size; and (6) time-domain signals were summed 300 times to improve the signal-to-noise ratio. Each soil DOM sample analyzed by FT-ICR MS was internally calibrated using known CHO-based compounds. The molecular formula was determined based on the following criteria: (1) a signal-to-noise ratio (S/N) greater than 6; (2) a mass deviation of less than 1 ppm; (3) a composition of CnHnOnN₀₋₅S₀₋₁; (4) a hydrogen-to-carbon ratio (H/C) between 0.2 and 2.3, and an oxygen-to-carbon ratio (O/C) between 0 and 1.2; (5) a mass-to-charge (m/z) value ranging from 100 to 800 Da; (6) data from mass spectral annotation software that validated the assignment of unique molecular formulas; and (7) occurrence in at least two of the three replicate samples.

**Sample grouping based on DOM MW_W_**

For FT-ICR MS analysis, 400 Da often serves as a critical transition point for DOM reactivity and microbial utilization. Previous incubation and field studies have demonstrated that k- and r-strategists showed different correlations with low (< 400 Da) and high (> 400 Da) molecular weight DOM[1]. In addition, in abiotic degradation processes such as photodegradation, the highly negatively correlated (degraded) components generally represent higher molecular weight compounds (> 400 Da), whereas newly formed photo-products are mainly < 400 Da[2]. Similarly, during advanced oxidation processes, the preferentially removed target molecules are predominantly high molecular weight (> 400 Da) rather than low molecular weight (< 400 Da)[3]. Thus, 400 Da reflects a meaningful biological and physicochemical boundary that separates relatively low molecular weight (LMW) DOM and relatively high molecular weight (HMW) DOM. In summary, we classified soil samples with a DOM MW_W_ of less than 400 Da as the LMW group, and those with a DOM MW_W_ greater than 400 Da as the HMW group. In addition, among three samples from a sampling site, if one sample has a DOM MW_W_ greater than 400 Da while the other two have DOM MW_W_ less than 400 Da, we classify the three samples from that site as LMW (MW_W_ < 400 Da).

**Co-occurrence patterns of microbes**

Microbial co-occurrence networks at the family level were constructed using the R package *WGCNA* v1.73. Due to the imbalance in sample size between the LMW and HMW groups, we used the R package *dplyr* v1.1.4 to randomly select 24 samples in each of the LMW and HMW groups, for a total of 5 randomizations (Table S16). To avoid potential spurious associations of rare microbes affecting reliability, the data were filtered prior to correlation calculations. The microbes present in 50% (12 samples) of the LMW or HMW samples, respectively, were selected for network construction based on Spearman’s rank correlation (|Spearman's *r*| > 0.8 and *P* < 0.01) of their relative abundances.

**Subsampling and metagenome-assembled genomes (MAGs) abundance calculation**

First, metagenomic coverage was calculated for each sample. Second, the lowest metagenomic coverage was determined across all metagenomic samples. In this study, the lowest coverage was 0.42. Finally, subsampling for each metagenomic sample was performed based on the lowest coverage by Npc_normalizer_manual.R (<https://github.com/baldeguer-riquelme/Npc_normalizer>)[4]. All abundance and diversity calculations were based on subsampled metagenomic data.

The relative abundance of MAGs was calculated as TAD80 (Truncated Average Depth at 80%) divided by genome equivalents (GEQ)[5]. TAD80 was calculated via CoverM v0.7.0[6] (-p bwa-mem --min-read-percent-identity 95 --min-read-aligned-percent 70 --min-covered-fraction 10 --exclude-supplementary -m trimmed_mean --trim-min 10 --trim-max 90). In detail, the calculation of TAD80 involved ranking the base pair positions of the MAG by their sequencing depth and then averaging the depths of the positions that remained after discarding the highest 10% and lowest 10% of positions. For a MAG detected in a metagenomic sample, at least 10% of the target sequence needed to be covered by clean reads (i.e., breadth of coverage) before its relative abundance is further calculated to provide reliable detection[7].

**Calculation of niche breadth and niche overlap score**

The niche breadth at the family level was calculated according to the Levins algorithm[8] using the R package *vegan* v2.6-10. The sum of the niche breadths in a sample was divided by the total number of species in the sample to obtain niche breadth at the community level in the sample[9]. The niche overlap scores for MAGs were performed following a recent study[10]. In short, microbial species (MAGs) with similar resource utilization capabilities or limitations have similar genome structures[10]. Thus, the Jaccard distance was calculated for each pair of the 132 MAGs (*D_ij_*, where *i* and *j* represent MAGs) based on the whole matrix representing the presence/absence of the 356 carbohydrate-active enzymes (CAZyme) genes annotated with dbCAN3. The calculation of the niche overlap score in MAGs was then performed according to the following equations[10]:

$$Niche overlap score (overall mean)=1-\frac{\sum_{i\neq j} D_{ij}}{N(N-1)}$$

where *D_ij_* is the Jaccard distance in CAZyme gene compositions of MAGs, and *N* is the number of MAGs detected. This niche overlap score based on Jaccard dissimilarity varies between 0 (CAZyme genes in all paired MAGs are completely different) and 1 (CAZyme genes in all paired MAGs are completely the same), thus allowing us to assess the level of niche overlap against carbon degradation functions of the target communities within a standardized range.

**Details of partial least square-structural equation modeling (PLS-SEM) construction**

To explore the effects of multiple variables on ARG abundance, PLS-SEM was employed to explore the direct, indirect, and interactive effects. Climate variables, including mean annual temperature (MAT) and annual precipitation (AP), were obtained from WorldClim v2.1 (https://worldclim.org/data/worldclim21.html) at the 30-second (~1 km^2^) resolution. Soil property variables, including pH and C&N (TOC and TN), were determined according to the methods described above. The variables of DOM molecular traits, including HMW (Relative abundance of HMW DOM molecules), Chao1 (Chemodiversity for DOM molecules), and MLB_R_ (Percentage of recalcitrant substance), were calculated according to ***Materials and methods*** and Table S2. The Shannon index (alpha diversity) and PC1 index (beta diversity, represented by the PC1 axis using PCoA analysis based on Bray-Curtis distance) were used as microbial community variables. The relative abundances of carbon metabolism genes (CMGs) and CAZyme genes were used as carbon metabolism variables. Indirect effects were defined as multiple path coefficients between the predictor and response variables including all possible paths excluding the direct effect. The final model selection was based on Cronbach’s alpha (C.alpha), Dillon-Goldstein’s rho (DG. rho), average variance extracted (AVE), Loading, and goodness of fit (GOF), which evaluate the model’s reliability and GOF.

**DNA extraction, library construction, and metagenomic/metatranscriptomic sequencing**

For the 141 soil samples collected across China, soil DNA was extracted using the ALFA-SEQ Advanced Soil DNA Kit (Findrop, China) according to the manufacturer’s instructions. For the 44 soil samples from microcosm experiments, the soil DNA and RNA were extracted using the RNeasy PowerSoil Total RNA Kit (Qiagen, Germany) and RNeasy PowerSoil DNA Elution Kit (Qiagen, Germany), respectively. The concentrations of DNA and RNA were then measured by Quantus Fluorometer (Promega, France) and NanoDrop 2000 spectrophotometer (Thermo Scientific, USA), respectively. The integrity of DNA and RNA was detected by 1% agarose gel electrophoresis. The extracted DNA was then fragmented to an average size of 400 bp using Covaris M220 (Gene, China) for creating a paired-end sequencing library with NEXTFLEX Rapid DNA-Seq (Bioo Scientific, USA). The RNA was subjected to standard Illumina library preparation with an Illumina Stranded mRNA Prep, Ligation (Illumina, USA), and rRNA was depleted using a RiboCop rRNA Depletion Kit for Mixed Bacterial Samples (Lexogen, USA). Finally, paired-end sequencing (2 × 150 bp) for metagenome and metatranscriptome was performed on a NovaSeq 6000 System (Illumina, USA) at Majorbio Bio-Pharm Technology Co., Ltd. (Shanghai, China).

**Preprocessing of metatranscriptomic data in microcosm experiments**

Metatranscriptomic raw data were trimmed using Trimmomatic v0.39[11] to remove adaptors, and low-quality reads (LEADING:3 TRAILING:3 SLIDINGWINDOW:5:20 MINLEN:50). SortMeRNA v4.3.4[12] was used to eliminate rRNA reads (--paired_in) to obtain mRNA reads.

**Identification of viral contigs from metagenomic datasets**

First, pre-screening of virus contigs was performed with the default parameters of geNomad and VirSorter2, respectively. And only contigs that were considered pure virus/provirus by geNomad and VirSorter2 were used for the next step of analysis. The results of the pre-screening were then quality controlled with CheckV, and potential host regions were trimmed from the ends of the provirus. Finally, viral contigs were determined based on the viral and host gene counts (virus_genes and host_genes in CheckV), virus scores (virus_score in geNomad and max_score in VirSorter2), hallmark gene counts (n_hallmarks in geNomad and hallmarks in VirSorter2), and viral contigs’ length (after removal of potential host regions of length > 5000 bp). Empirical screening criteria for soil viruses[13]: keep1) viral_gene > 0; keep2) viral_gene = 0 and host_gene = 0; keep3) viral_gene = 0 and virus_score & max_score >= 0.95; keep4) viral_gene = 0 and n_hallmarks & hallmark > 2. Most false positives can be removed by strict parameter screening.

**Supplementary results**

**The correlation of ARGs and DOM in relative abundance in 141 paddy soils**

ARGs assigned to macrolide-lincosamide-streptogramin (MLS), rifamycin, novobiocin, beta-lactam, tetracycline, vancomycin, tetracenomycin C, fosfomycin, aminoglycoside, and quinolone resistance significantly positively correlated with the relative abundance of HMW DOM molecules, and negatively correlated with the relative abundance of LMW DOM molecules. Among the DOM molecules that significantly positively correlated with the relative abundance of ARGs, an average of 76.5% of them belonged to High MW molecules, which was higher than that belonging to Low MW (23.5%; Fig. 1E).

**MAGs information from 141 paddy soils**

The genome sizes of these MAGs range from 65.45 to 99.03 Mb, and no significant difference in genome size was observed between co-hosts and non-hosts (*P* > 0.05; Fig. S7B). The non-hosts (not carrying ARGs; *n* = 64) were mainly assigned to *Actinomycetota* (20 MAGs), *Thermoproteota* (12 MAGs), *Pseudomonadota* (6 MAGs), and *Gemmatimonadota* (6 MAGs; Fig. 4A). The gene counts of the identified CAZyme genes and CMGs in these MAGs ranged from 9 to 359 and 22 to 220, respectively. There were 145, 136, 87, 55, 19, 9, and 111 biosynthetic gene clusters (BGCs) identified for the ribosomally synthesized and posttranslationally modified peptides (RiPPs), Terpene, nonribosomal synthetic peptides (NRPS), other polyketide synthases (PKS), PKS I, polyketide-nonribosomal peptide combinations (PKS-NRPS hybrids), and Others, respectively (Table S9). The 26 MAGs with increased abundance in the HMW than LMW DOM soils were assigned to *Pseudomonadota* (10 MAGs), *Acidobacteriota* (3 MAGs), *Nitrospirota* (2 MAGs), *Myxococcota* (2 MAGs), *Gemmatimonadota* (2 MAGs), *Chloroflexota* (2 MAGs), *Actinomycetota* (2 MAGs), *Desulfobacterota* (2 MAGs), and *Bacteroidota* (1 MAGs) at the phylum level (Fig. S9). These MAGs primarily carried multidrug (16 MAGs), polymyxin (7 MAGs), bacitracin (4 MAGs), novobiocin (4 MAGs), and rifamycin resistance genes (3 MAGs). Based on BiG-SCAPE analysis, the BGCs in 26 MAGs were classified into six classes, including RiPPs (21 MAGs), Terpene (21 MAGs), NRPS (14 MAGs), other PKS (7 MAGs), PKS-NRP Hybrids (1 MAG), and Others (16 MAGs; Fig. S9).

**Information of MAGs in microcosm experiments**

A total of 158 and 62 non-redundant high-quality MAGs (completeness ≥ 90% and contamination ≤ 5%) were recovered from LMW (HN) and HMW (FJ) soils, respectively (Tables S11 and S12). Of these, 110 and 33 co-hosts (MAGs) carrying both ARG and CMGs/CAZyme genes were identified in LMW and HMW soils, respectively. The co-hosts were mainly assigned to *Pseudomonadota* (60 MAGs in LMW soil and 18 MAGs in HMW soil) and *Bacteroidota* (27 MAGs in LMW soil and 5 MAGs in HMW soil).

**Information on viral host and AMGs in microcosm experiments**

About 10.36% (903) and 9.27% (357) of vOTUs were predicted to have potential hosts in LMW (HN) and HMW (FJ) soils, respectively (Fig. 6B). The predicted viral hosts originated from 19 and 17 phyla of bacteria and archaea in LMW and HMW soils, respectively (Tables S15 and S16). *Pseudomonadota* were the most frequently predicted viral hosts (407 vOTUs in LMW and 226 vOTUs in HMW soils), followed by *Bacillota* (223 vOTUs in LMW and 54 vOTUs in HMW soils). A small proportion of vOTUs (18 in LMW and 6 in HMW soil) were also identified as associated with archaea (Tables S15 and S16).

In HMW soil, the total relative abundance of vAMGs annotated as CAZyme genes was 5.6×10^5^ *vs.* 6.9×10^5^ *vs.* 4.2×10^5^ RPKM at DNA level and 9.4×10^5^ *vs.* 8.4×10^5^ *vs.* 2.7×10^5^ RPKM at RNA level for lignin, glucose and control treatment, respectively (Fig. 6C). For the LMW soil, the total relative abundance of vAMGs annotated as CAZyme genes was 5.2×10^5^ *vs*. 9.6×10^5^ *vs*. 4.7×10^5^ RPKM at DNA level and 7.0×10^5^ *vs.* 9.7×10^5^ *vs.* 5.5×10^5^ RPKM at RNA level for lignin, glucose and control treatment, respectively (Fig. 6C).

Specifically, the relative abundance of Glycoside Hydrolase (GH) family at DNA and RNA levels significantly (*P* < 0.05) increased after both the lignin (2.6×10^3^ and 2.4×10^4^ RPKM) and glucose addition (7.6×10^4^ and 3.1×10^5^ RPKM) than the control treatment (0 and 0 RPKM) in HMW soil (Fig. 6C). However, for the LMW soil, the relative abundance of the GH family significantly (*P* < 0.05) increased after DOM addition (8.7×10^5^ and 8.2×10^5^ RPKM) than the control treatment (5.1×10^4^ and 4.0×10^4^ RPKM) only at DNA level. For the Polysaccharide Lyase (PL) family, their relative abundance in HMW soil significantly (*P* < 0.05) increased after lignin addition (3.6×10^5^ RPKM) than the control treatment (2.3×10^5^ RPKM) at DNA level (Fig. 6C). However, for the LMW soil, the relative abundance of PL family at DNA and RNA levels significantly (*P* < 0.05) decreased after the glucose addition (6.4×10^3^ and 0 RPKM) compared to the control treatment (1.0×10^5^ and 3.6×10^4^ RPKM; Fig. 6C). The Glycosyl Transferase (GT) family, which was responsible for the biosynthesis of disaccharides, oligosaccharides, and polysaccharides was also analyzed. However, no significant variance in their relative abundance was observed for the HMW soil between DOM addition and the control treatment either at DNA or RNA level (Fig. 6C). Whereas the relative abundance of the GT family at the DNA level significantly (*P* < 0.05) decreased after glucose addition (9.4×10^4^ RPKM) compared to the control (3.2×10^5^ RPKM) in LMW soil.

**Supplementary figures**

**Figure S1** **Distribution of 47 sampling sites (3 replicates per site) in China.** Red and blue represent relatively low molecular weight (LMW) and relatively high molecular weight (HMW) soils, respectively. The bottom map shows the distribution of single-season rice in China (green)[14].

**Figure S2** **Frequency distribution of dissolved organic matter (DOM) MW_W_ in LMW** (A) **and HMW** (B) **soils.** Numbers above bars indicate the number of samples in each MW_W_ interval. Both selected soils (HN and FJ) fall within the modal (most frequent) MW_W_ interval of their respective groups.

**Figure S3** **Design of microcosm experiments with DOM addition.**

**Figure S4** **Community composition of DOM molecules and antibiotic resistance genes (ARGs).** (A) Distribution of unique DOM molecules in LMW and HMW soils. (B) Nonmetric multidimensional scaling (NMDS) analysis of a Bray-Curtis dissimilarity matrix calculated from the relative abundance of ARG subtypes (PERMANOVA test, *F* = 6.9, *P* = 0.001). MLB: molecular lability boundary, O/C: oxygen-carbon ratio, H/C: hydrogen-carbon ratio.

**Figure S5** **The profiles of ARG diversity (Richness index) and effect size of high-risk ARG types in LMW and HMW soils.** (A) Differences in ARG diversity between LMW and HMW soils. (B) Dot plots with error bars (standard error, SE) show the effect sizes of different high-risk ARG types. Linear mixed-effects models (LMMs) were used to compare differences in HMW versus LMW soils and perform significance tests. The standardized regression coefficient (*β*) was adopted as the effect size estimated from LMMs.

**Figure S6 The composition of potential human pathogens carrying ARGs.** (A) Pathogen host composition of ARGs at the phylum level. Each error bar corresponds to the standard deviation (SD), and data were showed as mean ± SD. (B) Dot plots with error bars (SE) show the effect sizes of different pathogen hosts of ARGs (*P* < 0.05). LMMs were used to compare differences in HMW versus LMW soils and perform significance tests.

**Figure S7 Factors influencing the ARG composition in LMW and HMW soils based on Mantel test.**

**Figure S8 Factors influencing soil resistomes in paddy soils.** (A) Mean predictor importance of soil properties, DOM molecular traits, and metal concentration on the relative abundance of ARGs based on random forest modeling (Permutation test, *n* = 1000, **P* < 0.05; ***P* < 0.01). (B) Covariate-adjusted relationships between relative abundance of HMW DOM and ARG at site-level (detailed in Fig. S1, *n* = 47). Points were 47 site means and lines show linear fits and shaded bands show 95% confidence interval (CI). Models adjusted for As concentration, microbial alpha diversity (Shannon), beta diversity (PC1), pH, TOC, TN, TP and TS. (C) Standardized coefficients and 95% CI for HMW DOM relative abundance and As concentration in the covariate-adjusted models.

**Figure S9 Characteristics of archaeal metagenome-assembled genomes (MAGs) and comparison of genome size of all MAGs.** (A) Phylogenetic tree of archaeal high-quality (completeness ≥ 90% and contamination ≤ 5%) MAGs. The colors of MAGs represented the different phylum levels to which these MAGs were assigned. The number of identified carbon metabolism genes (CMGs) and carbohydrate-active enzyme (CAZyme) genes among the MAGs were shown in the bar plots. AS&NS: amino sugar and nucleotide sugar, G&D: glyoxylate and dicarboxylate, S&S: starch and sucrose, PP: pentose phosphate, IP: inositol phosphate. (B) Genome size of co-hosts (*n* = 68) and non-hosts (*n* = 64). Two-sided Wilcoxon rank sum test was used for the significance test. n.s.: no significance.

**Figure S10** **Biosynthetic gene cluster (BGC) counts in MAGs and their abundances in LMW and HMW soils.** (A) Average BGC counts at the class level based on BiG-SCAPE in co-hosts and non-hosts. RiPPs: ribosomally synthesized and posttranslationally modified peptides, NRPS: nonribosomal synthetic peptides, PKS: polyketide synthases, PKS-NRPS hybrids: polyketide-nonribosomal peptide combinations. (B) Relative abundance of BGCs in LMW and HMW soils. LMMs were used to compare differences in HMW versus LMW soils and perform significance tests. The standardized regression coefficient (*β*) was adopted as the effect size estimated from LMMs.

**Figure S11** **Abundance variation of MAGs, ARGs in MAGs, and BGCs in MAGs from LMW to HMW soils.** Blank cells represent an abundance of 0. The colors of MAGs represented the different phylum levels to which these MAGs were assigned. MLS: macrolide-lincosamide-streptogramin.

**Figure S12** **Abundance of functional genes in microcosm experiments.** Relative abundances of ARGs (A), CMGs, and CAZyme genes (B) under different treatments (*n* = 7) at the DNA level in LMW (HN) and HMW (FJ) soils. Each error bar corresponds to the SD, and data were showed as mean + SD. The circles represented median values. LMMs were used to compare differences in treatments (glucose and lignin) versus control and perform significance tests. The standardized regression coefficient (*β*) was adopted as the effect size estimated from LMMs. *β_G_*: effect size of glucose treatment versus control, *β_L_*: effect size of lignin treatment versus control.

**Figure S13 The transcript ratio of ARGs to housekeeping genes under different treatments (*n* =7) in LMW (HN) and HMW (FJ) soils.** LMMs were used to compare differences in treatments (glucose and lignin) versus control and perform significance tests. The standardized regression coefficient (*β*) was adopted as the effect size estimated from LMMs.

**Figure S14 Relative abundance of BGCs at the DNA level in different treatments (*n* =7) in LMW (HN) and HMW (FJ) soils.** LMMs were used to compare differences in treatments (glucose and lignin) versus control and perform significance tests. The standardized regression coefficient (*β*) was adopted as the effect size estimated from LMMs.

**Figure S15 The average nucleotide identity (ANI) between MAGs based on FastANI**[15]**.**

**Figure S16** **Genomic co-localization of ARGs, CAZyme genes, and BGCs on representative MAGs.**

**Figure S17 Relative abundance of auxiliary metabolism genes (AMGs) annotated as CAZyme genes.** Each error bar corresponds to the SE, and data were showed as mean + SE. LMMs were used to compare differences in HMW versus LMW soils and perform significance tests. The standardized regression coefficient (*β*) was adopted as the effect size estimated from LMMs.

**Figure S18** **Total organic carbon and total nitrogen in LMW and HMW soils.** LMMs were used to compare differences in HMW versus LMW soils and perform significance tests. The standardized regression coefficient (*β*) was adopted as the effect size estimated from LMMs.

**Figure S19** **Comparison of niche breadth and niche overlap score.** (A) Niche breadth at the family level in LMW and HMW soils. LMMs were used to compare differences in HMW versus LMW soils and perform significance tests. The standardized regression coefficient (*β*) was adopted as the effect size estimated from LMMs. (B) Niche overlap score in co-hosts and non-hosts. Niche overlap score calculated based on the carbon degradation function (CAZyme gene) of MAGs (detailed in the Supplementary methods).

**Figure S20** **Microbial co-occurrence networks based on Spearman’s rank correlation.** (A) Proportion of negative correlations in microbial co-occurrence networks (5 randomizations, detailed in the Supplementary methods). (B) Comparison of the proportion of negative correlations in LMW and HMW soils (two-sided Wilcoxon rank sum test).

**Figure S21** **Relative abundance of *Actinomycetes* in LMW and HMW soils.** LMMs were used to compare differences in HMW versus LMW soils and perform significance tests. The standardized regression coefficient (*β*) was adopted as the effect size estimated from LMMs.

**Supplementary Tables**

**Table S1** Basic information of 141 paddy soil samples in China.

| Sample ID | Raw Reads | Raw Size (Gbp) | Clean Reads | Clean Size (Gbp) | Trim (%) | Nonpareil Coverage (%) | Group |
| --- | --- | --- | --- | --- | --- | --- | --- |
| AH_CZ_1 | 79877604 | 11.98 | 69846958 | 10.48 | 87.44 | 42.22 | LMW |
| AH_CZ_2 | 97442648 | 14.62 | 86337352 | 12.95 | 88.6 | 48.21 | LMW |
| AH_CZ_3 | 80108178 | 12.02 | 68077984 | 10.21 | 84.98 | 42.04 | LMW |
| AH_HN_1 | 94804480 | 14.22 | 86614378 | 12.99 | 91.36 | 58.59 | LMW |
| AH_HN_2 | 85090868 | 12.76 | 78304976 | 11.75 | 92.03 | 54.7 | LMW |
| AH_HN_3 | 89185698 | 13.38 | 81611152 | 12.24 | 91.51 | 55.5 | LMW |
| AH_SZ_1 | 92112016 | 13.82 | 81896304 | 12.28 | 88.91 | 49.77 | HMW |
| AH_SZ_2 | 76843942 | 11.53 | 67518274 | 10.13 | 87.86 | 41.67 | HMW |
| AH_SZ_3 | 77492252 | 11.62 | 69278230 | 10.39 | 89.4 | 45.12 | HMW |
| FJ_HT_1 | 86256840 | 12.94 | 76116492 | 11.42 | 88.24 | 47.06 | HMW |
| FJ_HT_2 | 90785236 | 13.62 | 79488324 | 11.92 | 87.56 | 48.44 | HMW |
| FJ_HT_3 | 82184620 | 12.33 | 72334824 | 10.85 | 88.02 | 44.4 | HMW |
| GD_LM_1 | 112227898 | 16.83 | 103490096 | 15.52 | 92.21 | 60.87 | LMW |
| GD_LM_2 | 92107366 | 13.82 | 84022232 | 12.6 | 91.22 | 55.21 | LMW |
| GD_LM_3 | 84521884 | 12.68 | 77224950 | 11.58 | 91.37 | 52.55 | LMW |
| GD_SG_1 | 87856108 | 13.27 | 80471512 | 11.87 | 91.59 | 51.26 | LMW |
| GD_SG_2 | 151921832 | 22.94 | 136080384 | 20.04 | 89.57 | 31.84 | LMW |
| GD_SG_3 | 81755796 | 12.35 | 74679706 | 11 | 91.34 | 53.22 | LMW |
| GD_XH_1 | 89160414 | 13.37 | 82245362 | 12.34 | 92.24 | 53.28 | HMW |
| GD_XH_2 | 94126580 | 14.12 | 87529202 | 13.13 | 92.99 | 55.6 | HMW |
| GD_XH_3 | 96222938 | 14.43 | 86530746 | 12.98 | 89.93 | 54.6 | HMW |
| GX_GL_1 | 79254566 | 11.89 | 72633732 | 10.9 | 91.65 | 51.68 | LMW |
| GX_GL_2 | 95254324 | 14.29 | 87824972 | 13.17 | 92.2 | 55.95 | LMW |
| GX_GL_3 | 102097808 | 15.31 | 93624922 | 14.04 | 91.7 | 59.27 | LMW |
| GX_TX_1 | 101261008 | 15.19 | 94164598 | 14.12 | 92.99 | 61.71 | HMW |
| GX_TX_2 | 68291840 | 10.24 | 61304442 | 9.2 | 89.77 | 46.74 | HMW |
| GX_TX_3 | 89609534 | 13.44 | 80902606 | 12.14 | 90.28 | 54.48 | HMW |
| GZ_KY_1 | 75819172 | 11.37 | 66624946 | 9.99 | 87.87 | 42.61 | LMW |
| GZ_KY_2 | 73082610 | 10.96 | 65275338 | 9.79 | 89.32 | 42.53 | LMW |
| GZ_KY_3 | 81765414 | 12.26 | 72190426 | 10.83 | 88.29 | 44.11 | LMW |
| GZ_TR_1 | 93831102 | 14.07 | 86773176 | 13.02 | 92.48 | 55.98 | LMW |
| GZ_TR_2 | 90602520 | 13.59 | 83314226 | 12.5 | 91.96 | 54.56 | LMW |
| GZ_TR_3 | 103522562 | 15.53 | 96398916 | 14.46 | 93.12 | 58.89 | LMW |
| GZ_XR_1 | 75080058 | 11.34 | 67471368 | 9.93 | 89.87 | 69.71 | LMW |
| GZ_XR_2 | 74055070 | 11.18 | 68107780 | 10.08 | 91.97 | 67.27 | LMW |
| GZ_XR_3 | 83467052 | 12.6 | 75595250 | 11.15 | 90.57 | 69.01 | LMW |
| HB_JZ_1 | 82806216 | 12.42 | 73234576 | 10.99 | 88.44 | 46.86 | LMW |
| HB_JZ_2 | 73358626 | 11 | 63568708 | 9.54 | 86.65 | 42.19 | LMW |
| HB_JZ_3 | 79651790 | 11.95 | 69475332 | 10.42 | 87.22 | 46.22 | LMW |
| HB_WH_1 | 89944916 | 13.49 | 81038530 | 12.16 | 90.1 | 49.85 | LMW |
| HB_WH_2 | 89102682 | 13.37 | 80169928 | 12.03 | 89.97 | 49.63 | LMW |
| HB_WH_3 | 90969680 | 13.65 | 81560346 | 12.23 | 89.66 | 49.11 | LMW |
| HLJ_DQ_1 | 103090250 | 15.46 | 94400512 | 14.16 | 91.57 | 59.24 | HMW |
| HLJ_DQ_2 | 114999878 | 17.25 | 105398718 | 15.81 | 91.65 | 63.34 | HMW |
| HLJ_DQ_3 | 101336298 | 15.2 | 92393326 | 13.86 | 91.17 | 59.4 | HMW |
| HLJ_YL_1 | 101818818 | 15.27 | 93673380 | 14.05 | 92 | 55.64 | LMW |
| HLJ_YL_2 | 87751898 | 13.16 | 81391390 | 12.21 | 92.75 | 58.73 | LMW |
| HLJ_YL_3 | 96335240 | 14.45 | 89075948 | 13.36 | 92.46 | 57.76 | LMW |
| HN_CZ_1 | 74321708 | 11.22 | 68772594 | 10.14 | 92.53 | 50.81 | LMW |
| HN_CZ_2 | 88517476 | 13.37 | 82261494 | 12.13 | 92.93 | 54.32 | LMW |
| HN_CZ_3 | 73566902 | 11.11 | 68336654 | 10.08 | 92.89 | 47.65 | LMW |
| HN_HH_1 | 91818242 | 13.86 | 84227828 | 12.44 | 91.73 | 57.4 | LMW |
| HN_HH_2 | 91639020 | 13.84 | 83076366 | 12.22 | 90.66 | 55.75 | LMW |
| HN_HH_3 | 82890598 | 12.52 | 74359538 | 10.95 | 89.71 | 52.93 | LMW |
| HN_HY_1 | 71618348 | 10.81 | 66582966 | 9.83 | 92.97 | 48.39 | LMW |
| HN_HY_2 | 81325924 | 12.28 | 75745960 | 11.18 | 93.14 | 49.34 | LMW |
| HN_HY_3 | 72509602 | 10.95 | 67357144 | 9.94 | 92.89 | 48.99 | LMW |
| HN_JY_1 | 81367686 | 12.21 | 72227914 | 10.83 | 88.77 | 46.94 | HMW |
| HN_JY_2 | 78926958 | 11.84 | 69338116 | 10.4 | 87.85 | 45.23 | HMW |
| HN_JY_3 | 80622898 | 12.09 | 69521580 | 10.43 | 86.23 | 45.95 | HMW |
| HN_LH_1 | 79636384 | 11.95 | 69638394 | 10.45 | 87.45 | 44.38 | HMW |
| HN_LH_2 | 90423378 | 13.56 | 79002032 | 11.85 | 87.37 | 47.66 | HMW |
| HN_LH_3 | 84428128 | 12.66 | 74531186 | 11.18 | 88.28 | 44.71 | HMW |
| HN_LY_1 | 88910278 | 13.43 | 82041404 | 12.13 | 92.27 | 60.18 | LMW |
| HN_LY_2 | 93373344 | 14.1 | 85673670 | 12.66 | 91.75 | 51.69 | LMW |
| HN_LY_3 | 81817302 | 12.35 | 75448288 | 11.16 | 92.22 | 47.17 | LMW |
| HN_SM_1 | 81782518 | 12.27 | 70370962 | 10.56 | 86.05 | 46.18 | HMW |
| HN_SM_2 | 82187292 | 12.33 | 71198300 | 10.68 | 86.63 | 45.67 | HMW |
| HN_SM_3 | 84816144 | 12.72 | 74825566 | 11.22 | 88.22 | 48.13 | HMW |
| HN_XX_1 | 85391972 | 12.81 | 78698388 | 11.8 | 92.16 | 60.01 | LMW |
| HN_XX_2 | 94733000 | 14.21 | 87025530 | 13.05 | 91.86 | 58.44 | LMW |
| HN_XX_3 | 88902674 | 13.34 | 82289442 | 12.34 | 92.56 | 55.08 | LMW |
| HN_YY_1 | 71793732 | 10.77 | 65887812 | 9.88 | 91.77 | 60.45 | LMW |
| HN_YY_2 | 86541888 | 12.98 | 80861634 | 12.13 | 93.44 | 56.31 | LMW |
| HN_YY_3 | 92268782 | 13.84 | 85239204 | 12.79 | 92.38 | 58.51 | LMW |
| HN_ZZ_1 | 89837576 | 13.48 | 83247410 | 12.49 | 92.66 | 55.57 | HMW |
| HN_ZZ_2 | 94745884 | 14.21 | 87153132 | 13.07 | 91.99 | 57.58 | HMW |
| HN_ZZ_3 | 85084036 | 12.76 | 78087536 | 11.71 | 91.78 | 54.66 | HMW |
| JL_CC_1 | 87928658 | 13.19 | 81174988 | 12.18 | 92.32 | 58.63 | HMW |
| JL_CC_2 | 92068606 | 13.81 | 85227468 | 12.78 | 92.57 | 56.58 | HMW |
| JL_CC_3 | 97373326 | 14.61 | 90043500 | 13.51 | 92.47 | 58.54 | HMW |
| JL_JH_1 | 92231468 | 13.83 | 84447514 | 12.67 | 91.56 | 54.04 | LMW |
| JL_JH_2 | 81246170 | 12.19 | 74844318 | 11.23 | 92.12 | 56.15 | LMW |
| JL_JH_3 | 97295280 | 14.59 | 89806286 | 13.47 | 92.3 | 54.97 | LMW |
| JL_SL_1 | 110992442 | 16.65 | 102810418 | 15.42 | 92.63 | 59.72 | LMW |
| JL_SL_2 | 114273888 | 17.14 | 105390684 | 15.81 | 92.23 | 58.75 | LMW |
| JL_SL_3 | 96722178 | 14.51 | 89209452 | 13.38 | 92.23 | 54.01 | LMW |
| JS_SZ_1 | 99523490 | 14.93 | 89221038 | 13.38 | 89.65 | 50.76 | HMW |
| JS_SZ_2 | 84148030 | 12.62 | 75534070 | 11.33 | 89.76 | 48.29 | HMW |
| JS_SZ_3 | 88164764 | 13.22 | 78684172 | 11.8 | 89.25 | 48.39 | HMW |
| JS_YX_1 | 99776112 | 14.97 | 91176522 | 13.68 | 91.38 | 56.03 | HMW |
| JS_YX_2 | 94631012 | 14.19 | 86869762 | 13.03 | 91.8 | 55.38 | HMW |
| JS_YX_3 | 107328808 | 16.1 | 98642018 | 14.8 | 91.91 | 59.62 | HMW |
| JX_GZ_1 | 259271390 | 39.15 | 233104864 | 34.41 | 89.91 | 56.38 | HMW |
| JX_GZ_2 | 246337422 | 37.2 | 223585790 | 33.01 | 90.76 | 60.87 | HMW |
| JX_GZ_3 | 214686028 | 32.42 | 183254830 | 26.92 | 85.36 | 59.03 | HMW |
| JX_NC_1 | 86522826 | 12.98 | 79779056 | 11.97 | 92.21 | 54.45 | LMW |
| JX_NC_2 | 105697860 | 15.85 | 98479846 | 14.77 | 93.17 | 58.4 | LMW |
| JX_NC_3 | 91797788 | 13.77 | 85621744 | 12.84 | 93.27 | 54.97 | LMW |
| LN_LY_1 | 135965364 | 20.39 | 125632822 | 18.84 | 92.4 | 63.73 | LMW |
| LN_LY_2 | 118148364 | 17.72 | 107848396 | 16.18 | 91.28 | 54.46 | LMW |
| LN_LY_3 | 94225736 | 14.13 | 85925120 | 12.89 | 91.19 | 53.19 | LMW |
| SD_TC_1 | 113725538 | 17.06 | 103620458 | 15.54 | 91.11 | 65.26 | LMW |
| SD_TC_2 | 101243814 | 15.19 | 92416598 | 13.86 | 91.28 | 62.23 | LMW |
| SD_TC_3 | 112982096 | 16.95 | 103313082 | 15.5 | 91.44 | 62.73 | LMW |
| SH_MH_1 | 93296028 | 13.99 | 86280108 | 12.94 | 92.48 | 55.85 | HMW |
| SH_MH_2 | 86049968 | 12.91 | 79645342 | 11.95 | 92.56 | 53.14 | HMW |
| SH_MH_3 | 106716408 | 16.01 | 98793988 | 14.82 | 92.58 | 56.75 | HMW |
| TJ_JN_1 | 105360872 | 15.8 | 96636736 | 14.5 | 91.72 | 63.66 | HMW |
| TJ_JN_2 | 89724880 | 13.46 | 82674692 | 12.4 | 92.14 | 59.78 | HMW |
| TJ_JN_3 | 78489888 | 11.77 | 70142696 | 10.52 | 89.37 | 58.44 | HMW |
| YN_CJ_1 | 78748358 | 11.81 | 69241910 | 10.39 | 87.93 | 42.13 | LMW |
| YN_CJ_2 | 84395226 | 12.66 | 74217586 | 11.13 | 87.94 | 43.93 | LMW |
| YN_CJ_3 | 79305884 | 11.9 | 69799802 | 10.47 | 88.01 | 46.48 | LMW |
| YN_DC_1 | 89725694 | 13.46 | 82768008 | 12.42 | 92.25 | 55.62 | LMW |
| YN_DC_2 | 100170440 | 15.03 | 92454732 | 13.87 | 92.3 | 57.18 | LMW |
| YN_DC_3 | 91573698 | 13.74 | 84310446 | 12.65 | 92.07 | 56.92 | LMW |
| YN_MG_1 | 83534614 | 12.61 | 75050802 | 11.01 | 89.84 | 48.22 | LMW |
| YN_MG_2 | 74647494 | 11.27 | 68810660 | 10.15 | 92.18 | 43.73 | LMW |
| YN_MG_3 | 75276872 | 11.37 | 69634930 | 10.28 | 92.51 | 46.9 | LMW |
| YN_YL_1 | 99030398 | 14.85 | 90656158 | 13.6 | 91.54 | 55.93 | HMW |
| YN_YL_2 | 103321334 | 15.5 | 95461002 | 14.32 | 92.39 | 59.22 | HMW |
| YN_YL_3 | 105192174 | 15.78 | 96651122 | 14.5 | 91.88 | 61.99 | HMW |
| ZJ_HZ_1 | 101927876 | 15.29 | 93937750 | 14.09 | 92.16 | 59.25 | LMW |
| ZJ_HZ_2 | 103616938 | 15.54 | 95648246 | 14.35 | 92.31 | 61.25 | LMW |
| ZJ_HZ_3 | 84965796 | 12.74 | 77649558 | 11.65 | 91.39 | 55.89 | LMW |
| ZJ_JH_1 | 89770102 | 13.47 | 79799994 | 11.97 | 88.89 | 47.13 | HMW |
| ZJ_JH_2 | 80684492 | 12.1 | 71071000 | 10.66 | 88.09 | 43.42 | HMW |
| ZJ_JH_3 | 77434072 | 11.62 | 68907480 | 10.34 | 88.99 | 46.93 | HMW |
| ZJ_QJ_1 | 81462680 | 12.3 | 74644042 | 10.99 | 91.63 | 45.63 | LMW |
| ZJ_QJ_2 | 80936090 | 12.22 | 74445334 | 10.98 | 91.98 | 46.52 | LMW |
| ZJ_QJ_3 | 82242124 | 12.42 | 75040436 | 11.06 | 91.24 | 47.9 | LMW |
| ZJ_QZ_1 | 100146850 | 15.02 | 93229926 | 13.98 | 93.09 | 57.04 | HMW |
| ZJ_QZ_2 | 110481266 | 16.57 | 101996164 | 15.3 | 92.32 | 59.66 | HMW |
| ZJ_QZ_3 | 89458748 | 13.42 | 82455802 | 12.37 | 92.17 | 54.42 | HMW |
| ZJ_SY_1 | 81128754 | 12.25 | 73834420 | 10.87 | 91.01 | 47.68 | LMW |
| ZJ_SY_2 | 82588072 | 12.47 | 76513052 | 11.31 | 92.64 | 47.93 | LMW |
| ZJ_SY_3 | 81569842 | 12.32 | 73963748 | 10.87 | 90.68 | 49.85 | LMW |
| ZJ_TZ_1 | 78065562 | 11.71 | 67936172 | 10.19 | 87.02 | 43.29 | LMW |
| ZJ_TZ_2 | 85908990 | 12.89 | 75065700 | 11.26 | 87.38 | 43.48 | LMW |
| ZJ_TZ_3 | 84537016 | 12.68 | 75636314 | 11.35 | 89.47 | 46.05 | LMW |

Note: HN_HH (HN) and FJ_HT (FJ) soils were used for subsequent microcosm experiments with DOM addition.

**Table S2** Calculation of DOM molecular traits.

| Traits | Description | Calculation method |
| --- | --- | --- |
| DBE | Double bond equivalents | $DBE=1+\frac{2C-H+N+P}{2}$ |
| NOSC | Nominal oxidation state of carbon | $NOSC=4-\frac{4C+H-2O-3N+5P-2S}{2}$ |
| AI_mod_ | Modified aromaticity index | ${AI}_{mod}=\frac{1+C-0.5O-S-0.5\left( N+P+H \right)}{C-0.5O-S-N-P}$ |
| O/C | Oxygen-carbon ratio | $O/C=\frac{O}{C}$ |
| H/C | Hydrogen-carbon ratio | $H/C=\frac{H}{C}$ |
| MLB_R_ | Percentage of recalcitrant substance | ${MLB}_{R}=\frac{{Num}_{\frac{H}{C}<1.5}}{Num}\times100$ |
| DBE_W_ | Intensity-weighted double bond equivalents | ${DBE}_{W}=\frac{\sum_{i=1}^{n} {DBE}_{i}\times I_{i}}{\sum_{i=1}^{n} I_{i}}$ |
| NOSC_W_ | Intensity-weighted nominal oxidation state of carbon | ${NOSC}_{W}=\frac{\sum_{i=1}^{n} {NOSC}_{i}\times I_{i}}{\sum_{i=1}^{n} I_{i}}$ |
| (AI_mod_)_W_ | Intensity-weighted modified aromaticity index | ${({AI}_{mod})}_{W}=\frac{\sum_{i=1}^{n} {{(AI}_{mod})}_{i}\times I_{i}}{\sum_{i=1}^{n} I_{i}}$ |
| O/C_W_ | Intensity-weighted oxygen-carbon ratio | ${O/C}_{W}=\frac{\sum_{i=1}^{n} {O/C}_{i}\times I_{i}}{\sum_{i=1}^{n} I_{i}}$ |
| H/C_W_ | Intensity-weighted hydrogen-carbon ratio | ${H/C}_{W}=\frac{\sum_{i=1}^{n} {H/C}_{i}\times I_{i}}{\sum_{i=1}^{n} I_{i}}$ |
| MW_W_ | Intensity-weighted molecular weight | ${MW}_{W}=\frac{\sum_{i=1}^{n} {MW}_{i}\times I_{i}}{\sum_{i=1}^{n} I_{i}}$ |

Note: *DBE_i_*, *NOSC_i_*, *(AI_mod_)_i_*, *O/C_i_*, *H/C_i_*, and *MW_i_* is the double bond equivalents, nominal oxidation state of carbon, modified aromaticity index, oxygen-carbon ratio, hydrogen-carbon ratio, and molecular weight of the i-th DOM molecule, respectively. *I_i_* is the peak intensity of the i-th DOM molecule.

**Table S3** Sensitivity analysis of different DOM molecular weight thresholds based on LMMs.

| Molecular weight threshold | LMW (*n*) | HMW (*n*) | Effect size (*β*) on ARG abundance | *P* value |
| --- | --- | --- | --- | --- |
| 390 Da | 75 | 66 | 0.41 | 0.037 |
| 395 Da | 84 | 57 | 0.47 | 0.016 |
| 400 Da (this study) | 87 | 54 | 0.55 | 0.0043 |
| 405 Da | 87 | 54 | 0.55 | 0.0043 |
| 410 Da | 102 | 39 | 0.66 | 0.0013 |
| 415 Da | 105 | 36 | 0.72 | 0.00056 |

**Table S4** A consolidated list of human pathogens.

| Human pathogens at species level | | | |
| --- | --- | --- | --- |
| *Abiotrophia defectiva* | *Chlamydia pneumoniae* | *Klebsiella pneumoniae* | *Porphyromonas circumdentaria* |
| *Achromobacter piechaudii* | *Chlamydia psittaci* | *Klebsiella rhinoscleromatis* | *Porphyromonas endodontalis* |
| *Achromobacter xylosoxidans* | *Chlamydia trachomatis* | *Klebsiella variicola* | *Porphyromonas gingivalis* |
| *Acidaminococcus fermentans* | *Chlamydophila caviae* | *Kluyvera ascorbata* | *Porphyromonas levii* |
| *Acinetobacter baumannii* | *Chlamydophila pneumoniae* | *Kluyvera cryocrescens* | *Porphyromonas macacae* |
| *Acinetobacter calcoaceticus* | *Chlamydophila psittaci* | *Kluyvera georgiana* | *Porphyromonas somerae* |
| *Acinetobacter haemolyticus* | *Chromobacterium violaceum* | *Kluyvera intermedia* | *Porphyromonas* spp. |
| *Acinetobacter johnsonii* | *Chryseobacterium balustinum* | *Kocuria rhizophila* | *Prevotella baroniae* |
| *Acinetobacter junii* | *Chryseobacterium meningosepticum* | *Kroppenstedtia eburnea* | *Prevotella bivia* |
| *Acinetobacter lwoffii* | *Citrobacter amalonaticus* | *Lactobacillus* sp. | *Prevotella buccae* |
| *Acinetobacter nosocomialis* | *Citrobacter braakii* | *Lactococcus garvieae* | *Prevotella buccalis* |
| *Acinetobacter parvus* | *Citrobacter farmeri* | *Lactococcus lactis* | *Prevotella corporis* |
| *Acinetobacter pittii* | *Citrobacter freundii* | *Leclercia adecarboxylata* | *Prevotella dentalis* |
| *Acinetobacter radioresistens* | *Citrobacter koseri* | *Legionella anisa* | *Prevotella denticola* |
| *Acinetobacter soli* | *Citrobacter rodentium* | *Legionella birminghamensis* | *Prevotella disiens* |
| *Acinetobacter ursingii* | *Citrobacter sedlakii* | *Legionella bozemanae* | *Prevotella enoeca* |
| *Actinobacillus actinomycetemcomitans* | *Citrobacter werkmanii* | *Legionella cherrii* | *Prevotella heparinolytica* |
| *Actinobacillus equuli* | *Citrobacter youngae* | *Legionella cincinnatiensis* | *Prevotella intermedia* |
| *Actinobacillus hominis* | *Clostridioides difficile* | *Legionella dumoffii* | *Prevotella loescheii* |
| *Actinobacillus lignieresii* | *Clostridium argentinense* | *Legionella feeleii* | *Prevotella melaninogenica* |
| *Actinobacillus pleuropneumoniae* | *Clostridium baratii* | *Legionella hackeliae* | *Prevotella nigrescens* |
| *Actinobacillus* spp. | *Clostridium bifermentans* | *Legionella jordanis* | *Prevotella oralis* |
| *Actinobacillus suis* | *Clostridium bolteae* | *Legionella lansingensis* | *Prevotella oris* |
| *Actinobacillus ureae* | *Clostridium botulinum* | *Legionella longbeachae* | *Prevotella oulora* |
| *Actinomadura madurae* | *Clostridium butyricum* | *Legionella micdadei* | *Prevotella ruminicola* |
| *Actinomadura pelletieri* | *Clostridium celerecrescens* | *Legionella oakridgensis* | *Prevotella* spp. |
| *Actinomyces bovis* | *Clostridium chauvoei* | *Legionella pneumophila* | *Prevotella tannerae* |
| *Actinomyces georgiae* | *Clostridium clostridioforme* | *Legionella rubrilucens* | *Prevotella veroralis* |
| *Actinomyces gerencseriae* | *Clostridium difficile* | *Legionella sainthelensi* | *Prevotella zoogleoformans* |
| *Actinomyces israelii* | *Clostridium fallax* | *Legionella tucsonensis* | *Propionibacterium acnes* |
| *Actinomyces meyeri* | *Clostridium haemolyticum* | *Legionella wadsworthii* | *Propionibacterium avidum* |
| *Actinomyces naeslundii* | *Clostridium hathewayi* | *Leifsonia aquatica* | *Propionibacterium granulosum* |
| *Actinomyces neuii* | *Clostridium histolyticum* | *Leishmania mexicana* | *Propionibacterium propionicus* |
| *Actinomyces odontolyticus* | *Clostridium innocuum* | *Leminorella grimontii* | *Propionibacterium* spp*.* |
| *Actinomyces radingae* | *Clostridium intestinale* | *Leminorella richardii* | *Proteus mirabilis* |
| *Actinomyces turicensis* | *Clostridium novyi* | *Leptospira alexanderi* | *Proteus penneri* |
| *Aerococcus sanguinicola* | *Clostridium perfringens* | *Leptospira alstonii* | *Proteus vulgaris* |
| *Aerococcus urinae* | *Clostridium ramosum* | *Leptospira borgpetersenii* | *Providencia alcalifaciens* |
| *Aerococcus viridans* | *Clostridium septicum* | *Leptospira inadai* | *Providencia heimbachae* |
| *Aeromonas caviae* | *Clostridium sordellii* | *Leptospira interrogans* | *Providencia rettgeri* |
| *Aeromonas dhakensis* | *Clostridium sporogenes* | *Leptospira kirschneri* | *Providencia rustigianii* |
| *Aeromonas diversa* | *Clostridium subterminale* | *Leptospira kmetyi* | *Providencia stuartii* |
| *Aeromonas hydrophila* | *Clostridium symbiosum* | *Leptospira meyeri* | *Pseudomonas aeruginosa* |
| *Aeromonas jandaei* | *Clostridium tertium* | *Leptospira noguchii* | *Pseudomonas alcaligenes* |
| *Aeromonas media* | *Clostridium tetani* | *Leptospira santarosai* | *Pseudomonas fluorescens* |
| Human pathogens at species level | | | |
| *Aeromonas popoffii* | *Cohnella hongkongensis* | *Leptospira weilii* | *Pseudomonas luteola* |
| *Aeromonas salmonicida* | *Collinsella aerofaciens* | *Leptotrichia buccalis* | *Pseudomonas oryzihabitans* |
| *Aeromonas sanarellii* | *Comamonas testosteroni* | *Leptotrichia goodfellowii* | *Pseudomonas pseudoalcaligenes* |
| *Aeromonas schubertii* | *Corynebacterium afermentans* | *Leptotrichia trevisanii* | *Pseudomonas putida* |
| *Aeromonas sobria* | *Corynebacterium amycolatum* | *Leptotrichia wadei* | *Pseudomonas stutzeri* |
| *Aeromonas taiwanensis* | *Corynebacterium argentoratense* | *Leuconostoc pseudomesenteroides* | *Pseudonocardia autotrophica* |
| *Aeromonas trota* | *Corynebacterium aurimucosum* | *Listeria ivanovii* | *Pseudoramibacter alactolyticus* |
| *Aeromonas veronii* | *Corynebacterium bovis* | *Listeria monocytogenes* | *Psychrobacter phenylpyruvicus* |
| *Afipia* spp. | *Corynebacterium diphtheriae* | *Listeria seeligeri* | *Rahnella aquatilis* |
| *Aggregatibacter actinomycetemcomitans* | *Corynebacterium glucuronolyticum* | *Listeria welshimeri* | *Ralstonia insidiosa* |
| *Aggregatibacter aphrophilus* | *Corynebacterium jeikeium* | *Lysinibacillus sphaericus* | *Ralstonia mannitolilytica* |
| *Aggregatibacter segnis* | *Corynebacterium kroppenstedtii* | *Mannheimia haemolytica* | *Ralstonia picketti* |
| *Alcaligenes faecalis* | *Corynebacterium kutscheri* | *Megamonas hypermegale* | *Ralstonia pickettii* |
| *Alcaligenes odorans* | *Corynebacterium macginleyi* | *Megasphaera* sp. | *Raoultella ornithinolytica* |
| *Alloiococcus otitis* | *Corynebacterium minutissimum* | *Metamycoplasma hominis* | *Rhodococcus equi* |
| *Alloprevotella tannerae* | *Corynebacterium propinquum* | *Methylobacterium mesophilicum* | *Rhodococcus erythropolis* |
| *Amycolatopsis orientalis* | *Corynebacterium pseudodiphthericum* | *Micrococcus luteus* | *Rhodococcus fascians* |
| *Anaerobiospirillum succiniciproducens* | *Corynebacterium pseudodiphtheriticum* | *Micromonas micros* | *Rhodococcus rhodnii* |
| *Anaerococcus lactolyticus* | *Corynebacterium pseudotuberculosis* | *Moellerella wisconsensis* | *Rhodococcus rhodochrous* |
| *Anaerococcus prevotii* | *Corynebacterium renale* | *Mogibacterium timidum* | *Rickettsia africae* |
| *Anaerococcus vaginalis* | *Corynebacterium resistens* | *Moraxella (Branhamella) catarrhalis* | *Rickettsia akari* |
| *Anaplasma phagocytophila* | *Corynebacterium riegelii* | *Moraxella (Branhamella) caviae* | *Rickettsia australis* |
| *Anaplasma phagocytophilum* | *Corynebacterium striatum* | *Moraxella (Branhamella) cuniculi* | *Rickettsia canadensis* |
| *Arachnia propionica* | *Corynebacterium tuberculostearicum* | *Moraxella (Branhamella) ovis* | *Rickettsia conorii* |
| *Arcanobacterium bernardiae* | *Corynebacterium ulcerans* | *Moraxella (Moxarella) atlantae* | *Rickettsia felis* |
| *Arcanobacterium haemolyticum* | *Corynebacterium urealyticum* | *Moraxella (Moxarella) bovis* | *Rickettsia honei* |
| *Arcanobacterium heamolyticum* | *Corynebacterium xerosis* | *Moraxella (Moxarella) lacunata* | *Rickettsia japonica* |
| *Arcanobacterium pyogenes* | *Coxiella burnetii* | *Moraxella (Moxarella) liquefaciens* | *Rickettsia massiliae* |
| *Arcobacter butzleri* | *Cronobacter malonaticus* | *Moraxella (Moxarella) nonliquefaciens* | *Rickettsia montanensis* |
| *Arcobacter cryaerophilus* | *Cronobacter muytjensii* | *Moraxella (Moxarella) osloensis* | *Rickettsia parkeri* |
| *Aspergillus fumigatus* | *Cronobacter sakazakii* | *Moraxella catarrhalis* | *Rickettsia prowazekii* |
| *Averyella dalhousiensis* | *Cronobacter* spp. | *Moraxella lacunata* | *Rickettsia rickettsii* |
| *Bacillus anthracis* | *Cronobacter turicensis* | *Moraxella lincolnii* | *Rickettsia sibirica* |
| *Bacillus cereus* | *Cronobacter universalis* | *Moraxella nonliquefaciens* | *Rickettsia typhi* |
| *Bacillus circulans* | *Cryptococcus neoformans* | *Moraxella osloensis* | *Rodentibacter pneumotropicus* |
| *Bacillus coagulans* | *Cupriavidus gilardii* | *Moraxella* spp. | *Roseomonas gilardii* |
| *Bacillus laterosporus* | *Cupriavidus pauculus* | *Morganella morganii* | *Rothia dentocariosa* |
| *Bacillus licheniformis* | *Cupriavidus respiraculi* | *Mycobacterium abscessus* | *Ruminococcus productus* |
| *Bacillus megaterium* | *Cutibacterium acnes* | *Mycobacterium africanum* | *Saccharomonospora viridis* |
| *Bacillus mycoides* | *Delftia acidovorans* | *Mycobacterium asiaticum* | *Saccharopolyspora rectivirgula* |
| *Bacillus pumilus* | *Dermabacter hominis* | *Mycobacterium avium* | *Salmonella bongori* |
| *Bacillus sphaericus* | *Dermatophilus congolensis* | *Mycobacterium basiliense* | *Salmonella choleraesuis* |
| *Bacillus subtilis* | *Desulfovibrio desulfuricans* | *Mycobacterium boenickei* | *Salmonella enterica* |
| *Bacillus thuringiensis* | *Dialister invisus* | *Mycobacterium bovis* | *Salmonella enteritidis* |
| *Bacteroides caccae* | *Dialister pneumosintes* | *Mycobacterium canariasense* | *Salmonella typhi* |
| Human pathogens at species level | | | |
| *Bacteroides distasonis* | *Dichelobacter nodosus* | *Mycobacterium canettii* | *Salmonella typhimurium* |
| *Bacteroides eggerthii* | *Dietzia maris* | *Mycobacterium caprae* | *Sebaldella termitidis* |
| *Bacteroides forsythus* | *Dolosigranulum pigrum* | *Mycobacterium celatum* | *Selenomonas artemidis* |
| *Bacteroides fragilis* | *Edwardsiella hoshinae* | *Mycobacterium chelonae* | *Selenomonas dianae* |
| *Bacteroides galacturonicus* | *Edwardsiella ictaluri* | *Mycobacterium chimaera* | *Selenomonas flueggei* |
| *Bacteroides merdae* | *Edwardsiella piscicida* | *Mycobacterium conspicuum* | *Selenomonas infelix* |
| *Bacteroides ovatus* | *Edwardsiella tarda* | *Mycobacterium cosmeticum* | *Selenomonas noxia* |
| *Bacteroides pectinophilus* | *Eggerthella lenta* | *Mycobacterium fortuitum* | *Serratia ficaria* |
| *Bacteroides splanchnicus* | *Ehrlichia chaffeensis* | *Mycobacterium franklinii* | *Serratia fonticola* |
| *Bacteroides stercoris* | *Ehrlichia ewingii* | *Mycobacterium genavense* | *Serratia liquefaciens* |
| *Bacteroides thetaiotaomicron* | *Ehrlichia muris* | *Mycobacterium goodii* | *Serratia marcescens* |
| *Bacteroides uniformis* | *Ehrlichia sennetsu* | *Mycobacterium gordonae* | *Serratia odorifera* |
| *Bacteroides ureolyticus* | *Eikenella corrodens* | *Mycobacterium haemophilum* | *Serratia plymuthica* |
| *Bacteroides vulgatus* | *Elizabethkingia anophelis* | *Mycobacterium houstonense* | *Serratia proteamaculans* |
| *Bartonella alsatica* | *Elizabethkingia meningoseptica* | *Mycobacterium immunogenum* | *Serratia rubidaea* |
| *Bartonella ancashi* | *Enterobacter aerogenes* | *Mycobacterium intracellulare* | *Shewanella algae* |
| *Bartonella bacilliformis* | *Enterobacter amnigenus* | *Mycobacterium kansasii* | *Shigella boydii* |
| *Bartonella clarridgeiae* | *Enterobacter asburiae* | *Mycobacterium lentiflavum* | *Shigella dysenteriae* |
| *Bartonella doshiae* | *Enterobacter cancerogenus* | *Mycobacterium leprae* | *Shigella flexneri* |
| *Bartonella elizabethae* | *Enterobacter cloacae* | *Mycobacterium mageritense* | *Shigella sonnei* |
| *Bartonella grahamii* | *Enterobacter gergoviae* | *Mycobacterium malmoense* | *Sphingomonas paucimobilis* |
| *Bartonella henselae* | *Enterobacter hormaechei* | *Mycobacterium marinum* | *Sphingomonas* spp. |
| *Bartonella koehlerae* | *Enterobacter kobei* | *Mycobacterium microti* | *Spirillum minus* |
| *Bartonella quintana* | *Enterobacter ludwigii* | *Mycobacterium mucogenicum* | *Sporothrix schenckii* |
| *Bartonella tribocorum* | *Enterobacter sakazakii* | *Mycobacterium neoaurum* | *Staphylococcus aureus* |
| *Bartonella vinsonii* | *Enterococcus avium* | *Mycobacterium peregrinum* | *Staphylococcus capitis* |
| *Bartonella washoensis* | *Enterococcus casseliflavus* | *Mycobacterium pinnipedii* | *Staphylococcus chromogenes* |
| *Bergeyella zoohelcum* | *Enterococcus durans* | *Mycobacterium porcinum* | *Staphylococcus cohnii* |
| *Bifidobacterium dentium* | *Enterococcus faecalis* | *Mycobacterium scrofulaceum* | *Staphylococcus epidermidis* |
| *Bilophila wadsworthia* | *Enterococcus faecium* | *Mycobacterium senegalense* | *Staphylococcus haemolyticus* |
| *Bordetella avium* | *Enterococcus flavescens* | *Mycobacterium shimoidei* | *Staphylococcus hominis* |
| *Bordetella bronchiseptica* | *Enterococcus gallinarum* | *Mycobacterium simiae* | *Staphylococcus hyicus* |
| *Bordetella holmesii* | *Enterococcus hirae* | *Mycobacterium smegmatis* | *Staphylococcus intermedius* |
| *Bordetella parapertussis* | *Enterococcus mundtii* | *Mycobacterium szulgai* | *Staphylococcus lugdunensis* |
| *Bordetella pertussis* | *Enterococcus raffinosus* | *Mycobacterium tuberculosis* | *Staphylococcus pasteuri* |
| *Borrelia afzelii* | *Erysipelothrix rhusiopathiae* | *Mycobacterium ulcerans* | *Staphylococcus pettenkoferi* |
| *Borrelia bavariensis* | *Escherichia albertii* | *Mycobacterium xenopi* | *Staphylococcus pseudintermedius* |
| *Borrelia bissettii* | *Escherichia coli* | *Mycobacteroides abscessus* | *Staphylococcus saprophyticus* |
| *Borrelia brasiliensis* | *Escherichia fergusonii* | *Mycolicibacterium smegmatis* | *Staphylococcus warneri* |
| *Borrelia burgdorferi* | *Escherichia hermannii* | *Mycoplasma fermentans* | *Staphylococcus xylosus* |
| *Borrelia caucasica* | *Escherichia vulneris* | *Mycoplasma genitalium* | *Stenotrophomonas maltophilia* |
| *Borrelia crocidurae* | *Eubacterium brachy* | *Mycoplasma hominis* | *Streptobacillus moniliformis* |
| *Borrelia dipodilli* | *Eubacterium combesii* | *Mycoplasma pneumoniae* | *Streptococcus acidominimus* |
| *Borrelia duttonii* | *Eubacterium contortum* | *Mycoplasma salivarium* | *Streptococcus agalactiae* |
| *Borrelia garinii* | *Eubacterium cylindroides* | *Mycoplasma* spp. | *Streptococcus anginosus* |
| *Borrelia hermsii* | *Eubacterium limosum* | *Mycoplasmoides gallisepticum* | *Streptococcus bovis* |
| *Borrelia hispanica* | *Eubacterium moniliforme* | *Mycoplasmoides genitalium* | *Streptococcus canis* |
| Human pathogens at species level | | | |
| *Borrelia latyschewii* | *Eubacterium multiforme* | *Mycoplasmoides pneumoniae* | *Streptococcus constellatus* |
| *Borrelia lusitaniae* | *Eubacterium nodatum* | *Mycoplasmopsis agalactiae* | *Streptococcus criceti* |
| *Borrelia mayonii* | *Eubacterium rectale* | *Mycoplasmopsis caviae* | *Streptococcus dysgalactiae* |
| *Borrelia mazzottii* | *Eubacterium saburreum* | *Myroides odoratus* | *Streptococcus equi* |
| *Borrelia merionesi* | *Eubacterium saphenum* | *Neisseria cinerea* | *Streptococcus gallolyticus* |
| *Borrelia microti* | *Eubacterium sulci* | *Neisseria elongata* | *Streptococcus gordonii* |
| *Borrelia miyamotoi* | *Eubacterium tenue* | *Neisseria flava* | *Streptococcus iniae* |
| *Borrelia parkeri* | *Ewingella americana* | *Neisseria flavescens* | *Streptococcus intermedius* |
| *Borrelia persica* | *Exophiala dermatitidis* | *Neisseria gonorrhoeae* | *Streptococcus milleri* |
| *Borrelia recurrentis* | *Fibrobacter intestinalis* | *Neisseria lactamica* | *Streptococcus mitis* |
| *Borrelia spielmanii* | *Filifactor alocis* | *Neisseria meningitidis* | *Streptococcus mutans* |
| *Borrelia turicatae* | *Finegoldia magna* | *Neisseria mucosa* | *Streptococcus pneumoniae* |
| *Borrelia valaisiana* | *Flavobacterium psychrophilum* | *Neisseria perflava* | *Streptococcus pyogenes* |
| *Borrelia venezuelensis* | *Fluoribacter bozemanae* | *Neisseria sicca* | *Streptococcus salivarius* |
| *Borreliella burgdorferi* | *Fluoribacter dumoffii* | *Neisseria subflava* | *Streptococcus sanguis* |
| *Brachyspira aalborgi* | *Fluoribacter gormanii* | *Neisseria weaveri* | *Streptococcus sobrinus* |
| *Brachyspira pilosicoli* | *Francisella hispaniensis* | *Neorickettsia sennetsu* | *Streptococcus suis* |
| *Brachyspira* spp. | *Francisella philomiragia* | *Niallia circulans* | *Streptococcus uberis* |
| *Branhamella catarrhalis* | *Francisella tularensis* | *Nocardia abscessus* | *Sutterella wadsworthensis* |
| *Brevibacillus brevis* | *Fusarium oxysporum* | *Nocardia asteroides* | *Suttonella indologenes* |
| *Brevibacterium* spp. | *Fusobacterium mortiferum* | *Nocardia brasiliensis* | *Tannerella forsythia* |
| *Brevundimonas diminuta* | *Fusobacterium necrophorum* | *Nocardia brevicatena* | *Tatlockia maceachernii* |
| *Brevundimonas vesicularis* | *Fusobacterium nucleatum* | *Nocardia carnea* | *Tatlockia micdadei* |
| *Brucella abortus* | *Fusobacterium periodonticum* | *Nocardia caviae* | *Tatumella ptyseos* |
| *Brucella canis* | *Fusobacterium ulcerans* | *Nocardia cyriacigeorgica* | *Toxoplasma gondii* |
| *Brucella inopinata* | *Fusobacterium varium* | *Nocardia farcinica* | *Treponema carateum* |
| *Brucella melitensis* | *Gardnerella vaginalis* | *Nocardia nova* | *Treponema denticola* |
| *Brucella suis* | *Gemella haemolysans* | *Nocardia otitidiscaviarum* | *Treponema pallidum* |
| *Burkholderia cenocepacia* | *Gemella morbillorum* | *Nocardia paucivorans* | *Treponema pertenue* |
| *Burkholderia cepacia* | *Globicatella sanguinis* | *Nocardia pseudobrasiliensis* | *Treponema vincentii* |
| *Burkholderia contaminans* | *Gordonia amarae* | *Nocardia transvalensis* | *Trichophyton rubrum* |
| *Burkholderia dolosa* | *Gordonia bronchialis* | *Nocardia veterana* | *Tropheryma whippelii* |
| *Burkholderia gladioli* | *Gordonia otitidis* | *Nocardia wallacei* | *Tropheryma whipplei* |
| *Burkholderia mallei* | *Gordonia rubropertincta* | *Nocardiopsis dassonvillei* | *Trueperella bernardiae* |
| *Burkholderia multivorans* | *Gordonia sputi* | *Ochrobactrum anthropi* | *Trueperella pyogenes* |
| *Burkholderia pseudomallei* | *Gordonia terrae* | *Oligella ureolytica* | *Tsukamurella inchonensis* |
| *Burkholderia vietnamiensis* | *Granulicatella adjacens* | *Oligella urethralis* | *Tsukamurella paurometabola* |
| *Buttiauxella gaviniae* | *Haemophilus aegyptius* | *Orientia tsutsugamushi* | *Tsukamurella pulmonis* |
| *Campylobacter coli* | *Haemophilus aphrophilus* | *Paenibacillus alvei* | *Tsukamurella tyrosinosolvens* |
| *Campylobacter concisus* | *Haemophilus ducreyi* | *Paenibacillus glucanolyticus* | *Ureaplasma parvum* |
| *Campylobacter curvus* | *Haemophilus haemolyticus* | *Paenibacillus larvae* | *Ureaplasma urealyticum* |
| *Campylobacter fetus* | *Haemophilus influenzae* | *Paenibacillus macerans* | *Veillonella atypica* |
| *Campylobacter gracilis* | *Haemophilus parahaemolyticus* | *Paenibacillus polymyxa* | *Veillonella dispar* |
| *Campylobacter hyointestinalis* | *Haemophilus parainfluenzae* | *Paenibacillus popilliae* | *Veillonella parvula* |
| *Campylobacter jejuni* | *Haemophilus paraphrophilus* | *Paeniclostridium sordellii* | *Vibrio alginolyticus* |
| *Campylobacter lari* | *Haemophilus segnis* | *Pantoea agglomerans* | *Vibrio cholerae* |
| *Campylobacter rectus* | *Hafnia alvei* | *Parabacteroides distasonis* | *Vibrio cincinnatiensis* |
| Human pathogens at species level | | | |
| *Campylobacter sputorum* | *Hathewaya histolytica* | *Paracoccidioides brasiliensis* | *Vibrio fluvialis* |
| *Campylobacter upsaliensis* | *Helicobacter bilis* | *Paracoccus yeei* | *Vibrio furnissii* |
| *Candida albicans* | *Helicobacter bizzozeronii* | *Parvimonas micra* | *Vibrio harveyi* |
| *Candida glabrata* | *Helicobacter bovis* | *Pasteurella aerogenes* | *Vibrio hollisae* |
| *Candida parapsilosis* | *Helicobacter canadensis* | *Pasteurella caballi* | *Vibrio mimicus* |
| *Capnocytophaga canimorsus* | *Helicobacter cinaedi* | *Pasteurella canis* | *Vibrio parahaemolyticus* |
| *Capnocytophaga cynodegmi* | *Helicobacter felis* | *Pasteurella dagmatis* | *Vibrio vulnificus* |
| *Capnocytophaga gingivalis* | *Helicobacter fennelliae* | *Pasteurella multocida* | *Virgibacillus pantothenticus* |
| *Capnocytophaga granulosa* | *Helicobacter heilmannii* | *Pasteurella pneumotropica* | *Weissella confusa* |
| *Capnocytophaga haemolytica* | *Helicobacter pullorum* | *Pasteurella stomatis* | *Wolinella succinogenes* |
| *Capnocytophaga ochracea* | *Helicobacter pylori* | *Pediococcus acidilactici* | *Yersinia bercovieri* |
| *Capnocytophaga sputigena* | *Helicobacter salomonis* | *Peptinophilus asaccharolyticus* | *Yersinia enterocolitica* |
| *Cardiobacterium hominis* | *Helicobacter suis* | *Peptococcus niger* | *Yersinia frederiksenii* |
| *Cardiobacterium valvarum* | *Helicobacter winghamensis* | *Peptostreptococcus anaerobius* | *Yersinia intermedia* |
| *Cedecea davisae* | *Kingella denitrificans* | *Photobacterium damselae* | *Yersinia kristensenii* |
| *Cedecea lapagei* | *Kingella kingae* | *Photorhabdus asymbiotica* | *Yersinia mollaretii* |
| *Cedecea neteri* | *Klebsiella aerogenes* | *Plasmodium falciparum* | *Yersinia pestis* |
| *Cellulomonas cellulans* | *Klebsiella granulomatis* | *Plesiomonas shigelloides* | *Yersinia pseudotuberculosis* |
| *Cellulomonas turbata* | *Klebsiella ornithinolytica* | *Pluralibacter gergoviae* | *Yersinia rohdei* |
| *Centipeda periodontii* | *Klebsiella oxytoca* | *Porphyromonas asaccharolytica* | *Yersinia ruckeri* |
| *Chlamydia abortus* | *Klebsiella ozaenae* | *Porphyromonas catoniae* | *Yokenella regensburgei* |
| *Chlamydia felis* |  |  |  |

**Table S5** Sampling information for microcosm experiments.

| Soil | Before  (*n* = 2) | Treatment | Day1  (*n* = 6) | Day7  (*n* = 18) | Day15  (*n* = 18) |
| --- | --- | --- | --- | --- | --- |
| HN soil  (LMW) | H-D0 | CK | HCK-D1 | HCK-D7-1 | HCK-D15-1 |
|  |  |  |  | HCK-D7-2 | HCK-D15-2 |
|  |  |  |  | HCK-D7-3 | HCK-D15-3 |
|  |  | +Glucose | HG-D1 | HG-D7-1 | HG-D15-1 |
|  |  |  |  | HG-D7-2 | HG-D15-2 |
|  |  |  |  | HG-D7-3 | HG-D15-3 |
|  |  | +Lignin | HL-D1 | HL-D7-1 | HL-D15-1 |
|  |  |  |  | HL-D7-2 | HL-D15-2 |
|  |  |  |  | HL-D7-3 | HL-D15-3 |
| FJ soil  (HMW) | F-D0 | CK | FCK-D1 | FCK-D7-1 | FCK-D15-1 |
|  |  |  |  | FCK-D7-2 | FCK-D15-2 |
|  |  |  |  | FCK-D7-3 | FCK-D15-3 |
|  |  | +Glucose | FG-D1 | FG-D7-1 | FG-D15-1 |
|  |  |  |  | FG-D7-2 | FG-D15-2 |
|  |  |  |  | FG-D7-3 | FG-D15-3 |
|  |  | +Lignin | FL-D1 | FL-D7-1 | FL-D15-1 |
|  |  |  |  | FL-D7-2 | FL-D15-2 |
|  |  |  |  | FL-D7-3 | FL-D15-3 |

**Table S6** Relative abundance (copies/cell) of core ARG type in LMW and HMW soils.

| ARG type | Relative abundance (Median value) | | *β*  (linear mixed-effects model) | *P* value  (Wald type Ⅱ χ^2^ test) |
| --- | --- | --- | --- | --- |
|  | LMW | HMW |  |  |
| multidrug | 0.031947461 | 0.032135193 | 0.259979 | 0.181928 |
| rifamycin | 0.017524155 | 0.019952279 | 0.371083 | 0.196164 |
| bacitracin | 0.016724241 | 0.017354944 | 0.281801 | 0.293082 |
| novobiocin | 0.007804628 | 0.011400737 | 0.673169 | 0.012301 |
| polymyxin | 0.009131465 | 0.00897131 | 0.167598 | 0.389429 |
| MLS | 0.005585036 | 0.006653769 | 0.650799 | 0.00686 |
| tetracycline | 0.003489962 | 0.005662534 | 0.749172 | 0.000605 |
| beta-lactam | 0.002548112 | 0.003640601 | 0.425997 | 0.01648 |
| aminoglycoside | 0.002091183 | 0.003335533 | 0.524426 | 0.002504 |
| vancomycin | 0.001558741 | 0.003176537 | 0.036123 | 0.902684 |
| sulfonamide | 0.002027447 | 0.002124469 | 0.238036 | 0.350164 |
| mupirocin | 0.001859434 | 0.002411062 | 0.265895 | 0.33745 |
| quinolone | 0.000866563 | 0.001172144 | 0.390611 | 0.037527 |
| tetracenomycin C | 0.000218838 | 0.000625663 | 0.655848 | 0.008066 |
| trimethoprim | 0.000512353 | 0.000459763 | -0.175321 | 0.443342 |
| fosfomycin | 8.61786E-05 | 0.000298137 | 0.662278 | 0.00475 |
| chloramphenicol | 0.000151184 | 0.000247679 | 0.450119 | 0.06475 |
| tiamulin | 0.000191527 | 0.000209281 | 0.364485 | 0.147917 |

MLS: macrolide-lincosamide-streptogramin

**Table S7** ARGs with significant changes in relative abundance in LMW and HMW soils.

| ARG type | ARG subtype | *β*  (linear mixed-effects model) | *P* value  (Wald type Ⅱ χ^2^ test) |
| --- | --- | --- | --- |
| aminoglycoside | *AAC(2')-Id* | 0.371258 | 0.029913 |
| aminoglycoside | *AAC(3)-IIIb* | 0.421678 | 0.016985 |
| aminoglycoside | *AAC(3)-IVb* | 0.573563 | 0.028747 |
| aminoglycoside | *AAC(3)-IXa* | 0.453294 | 0.013534 |
| aminoglycoside | *AAC(6')-Ib-SK* | 0.406278 | 0.037929 |
| aminoglycoside | *aadA12* | 0.451541 | 0.049357 |
| aminoglycoside | *aadA16* | 0.528202 | 0.001665 |
| aminoglycoside | *aadK* | 0.400362 | 0.027531 |
| aminoglycoside | *ANT(6)-Ia* | 0.396141 | 0.030239 |
| beta-lactam | *ALG6-1* | 0.47862 | 0.013411 |
| beta-lactam | *BcIII* | 0.474157 | 0.00506 |
| beta-lactam | *BKC-1* | 0.375986 | 0.03622 |
| beta-lactam | *GRD33-1* | 0.45439 | 0.042808 |
| beta-lactam | *IDC-1* | 0.378774 | 0.030578 |
| beta-lactam | *LRA-7* | 0.485202 | 0.03447 |
| beta-lactam | *OXA-29* | 0.344285 | 0.044565 |
| beta-lactam | *OXA-37* | 0.341507 | 0.046364 |
| beta-lactam | *OXA-444* | 0.365191 | 0.032794 |
| chloramphenicol | *fexA* | 0.496586 | 0.007055 |
| fosfomycin | *FosB* | 0.664141 | 0.007904 |
| fosfomycin | *FosM2* | 0.544673 | 0.01797 |
| MLS | *carA* | 0.527426 | 0.003626 |
| MLS | *erm(39)* | 0.449179 | 0.008695 |
| MLS | *erm(40)* | 0.500211 | 0.006324 |
| MLS | *erm(A)* | 0.383008 | 0.030711 |
| MLS | *erm(C)* | 0.512902 | 0.026591 |
| MLS | *erm(Y)* | 0.388322 | 0.035427 |
| MLS | *gimA* | 0.498027 | 0.02587 |
| MLS | *ole(C)* | 0.697397 | 0.001694 |
| MLS | *optrA* | 0.508004 | 0.040618 |
| MLS | *srm(B)* | 0.384863 | 0.032377 |
| MLS | *vga(A)* | 0.381999 | 0.025333 |
| MLS | *vmlR* | 0.415782 | 0.035432 |
| multidrug | *adeF* | 0.451803 | 0.034811 |
| multidrug | *blt* | 0.616812 | 0.013534 |
| multidrug | *mdtL* | 0.381614 | 0.034351 |
| multidrug | *MexC* | 0.432229 | 0.019833 |
| multidrug | *MexD* | 0.341361 | 0.046461 |
| novobiocin | *novA* | 0.673169 | 0.012301 |
| other peptide antibiotics | *tsnR* | 0.38544 | 0.040392 |
| ARG type | ARG subtype | *β*  (linear mixed-effects model) | *P* value  (Wald type Ⅱ χ^2^ test) |
| rifamycin | *arr-2* | 0.422936 | 0.01353 |
| rifamycin | *iri* | 0.703516 | 0.004405 |
| tetracenomycin C | *tcmA* | 0.655848 | 0.008066 |
| tetracycline | *otr(A)* | 0.531725 | 0.045951 |
| tetracycline | *tet(40)* | 0.423306 | 0.012834 |
| tetracycline | *tet(44)* | 0.440591 | 0.0417 |
| tetracycline | *tet(47)* | 0.499386 | 0.00925 |
| tetracycline | *tet(48)* | 0.369691 | 0.030636 |
| tetracycline | *tet(L)* | 0.549134 | 0.04125 |
| tetracycline | *tet(M)* | 0.547917 | 0.009167 |
| tetracycline | *tet(O)* | 0.458409 | 0.022335 |
| tetracycline | *tetA(48)* | 0.728537 | 0.002676 |
| tetracycline | *tetB(46)* | 0.383498 | 0.046573 |
| tetracycline | *tetB(48)* | 0.523438 | 0.021892 |
| tetracycline | *tetX2* | 0.344534 | 0.044407 |
| vancomycin | *vanH_in_vanP_cl* | 0.365079 | 0.03285 |
| vancomycin | *vanHB* | 0.398345 | 0.023146 |
| aminoglycoside | *aadA9** | -0.39367 | 0.038604 |
| multidrug | *acrF** | -0.55723 | 0.030657 |
| multidrug | *acrA** | -0.39068 | 0.036384 |
| multidrug | *smeE** | -0.57651 | 0.02615 |
| polymyxin | *arnA** | -0.50226 | 0.034408 |
| polymyxin | *mcr-5.2** | -0.44673 | 0.0109 |
| trimethoprim | *dfrB2** | -0.429 | 0.041104 |

Note: ARG subtypes marked with an asterisk (*) were enriched in the LMW, whereas the remaining ARG subtypes were enriched in the HMW.

**Table S8** The independent effects of DOM molecular weight on ARG abundance using partial RDA.

| Model | Df | Variance | *F* | Pr (>*F*) | Adjusted *R*^2^ |
| --- | --- | --- | --- | --- | --- |
| MW | 1 | 0.009677 | 3.2273 | 0.002 | 4.1% |
| Residual | 51 | 0.152922 | - | - | - |

**Table S9** BGCs counts at class level in MAGs of 141 paddy soil.

| BiG-SCAPE Class | Co-hosts | Non-hosts | Total |
| --- | --- | --- | --- |
| RiPPs | 103 | 42 | 145 |
| Terpene | 90 | 46 | 136 |
| NRPS | 64 | 23 | 87 |
| other PKS | 38 | 17 | 55 |
| PKS-NRP hybrids | 8 | 1 | 9 |
| type I PKS | 6 | 13 | 19 |
| others | 78 | 33 | 111 |

**Table S10** The shared and unique BGC product prediction in co-hosts and non-hosts.

| BGC Product Prediction | BiG-SCAPE Class | Group |
| --- | --- | --- |
| hglE-KS | other PKS | Unique in co-hosts |
| microviridin | RiPPs | Unique in co-hosts |
| lanthipeptide-class-v | RiPPs | Unique in co-hosts |
| indole | others | Unique in co-hosts |
| triceptide | others | Unique in co-hosts |
| NRPS.T1PKS | PKS-NRP hybrids | Unique in co-hosts |
| PpyS-KS | others | Unique in co-hosts |
| CDPS | others | Unique in co-hosts |
| NRPS.NRP-metallophore | others | Unique in co-hosts |
| T3PKS.hserlactone | others | Unique in co-hosts |
| NI-siderophore | others | Unique in co-hosts |
| acyl_amino_acids | others | Unique in co-hosts |
| proteusin | RiPPs | Unique in co-hosts |
| LAP | RiPPs | Unique in co-hosts |
| lanthipeptide-class-iv | RiPPs | Unique in co-hosts |
| thiopeptide | RiPPs | Unique in co-hosts |
| NRPS.blactam | others | Unique in co-hosts |
| PKS-like | other PKS | Unique in co-hosts |
| NRPS.lanthipeptide-class-ii | others | Unique in co-hosts |
| phosphonate.NRPS.T1PKS | others | Unique in co-hosts |
| oligosaccharide.hglE-KS.T1PKS | others | Unique in co-hosts |
| lanthipeptide-class-i | RiPPs | Unique in co-hosts |
| nucleoside | others | Unique in co-hosts |
| lassopeptide | RiPPs | Unique in co-hosts |
| NRPS | NRPS | Shared |
| lanthipeptide-class-ii | RiPPs | Shared |
| T1PKS | type I PKS | Shared |
| betalactone | others | Shared |
| terpene | Terpene | Shared |
| RRE-containing | RiPPs | Shared |
| ectoine | others | Shared |
| ranthipeptide | RiPPs | Shared |
| T3PKS | other PKS | Shared |
| thioamitides | RiPPs | Shared |
| RiPP-like | RiPPs | Shared |
| NRPS-like | NRPS | Shared |
| arylpolyene | others | Shared |
| hserlactone | others | Shared |
| redox-cofactor | RiPPs | Shared |
| NAGGN | others | Shared |
| hydrogen-cyanide | others | Shared |
| phosphonate | others | Shared |
| NAPAA | NRPS | Shared |
| other | others | Shared |
| butyrolactone | others | Shared |
| HR-T2PKS | others | Unique in non-hosts |
| T1PKS.NRPS | PKS-NRP hybrids | Unique in non-hosts |
| arylpolyene.resorcinol | others | Unique in non-hosts |
| resorcinol | others | Unique in non-hosts |
| terpene.arylpolyene | others | Unique in non-hosts |
| oligosaccharide | Saccharides | Unique in non-hosts |

**Table S11** The information of 158 non-redundant high-quality MAGs (completeness ≥ 90% and contamination ≤ 5%) in LMW soil in microcosm experiments.

| ID | ARG | Domain | Phylum | CMG | CAZyme | Lignin | Chitin | Cellulose | GH |
| --- | --- | --- | --- | --- | --- | --- | --- | --- | --- |
| 1 | Yes | *Bacteria* | *Acidobacteriota* | 93 | 25 | 0 | 4 | 1 | 9 |
| 2 | Yes | *Bacteria* | *Actinomycetota* | 85 | 19 | 0 | 3 | 0 | 6 |
| 3 | Yes | *Bacteria* | *Actinomycetota* | 143 | 34 | 0 | 4 | 3 | 23 |
| 4 | Yes | *Bacteria* | *Actinomycetota* | 165 | 46 | 3 | 5 | 3 | 35 |
| 5 | Yes | *Bacteria* | *Bacillota* | 26 | 14 | 0 | 2 | 0 | 5 |
| 6 | Yes | *Bacteria* | *Bacillota* | 25 | 14 | 0 | 2 | 0 | 4 |
| 7 | Yes | *Bacteria* | *Bacillota_A* | 86 | 22 | 0 | 2 | 0 | 9 |
| 8 | Yes | *Bacteria* | *Bacillota_A* | 75 | 19 | 0 | 3 | 0 | 7 |
| 9 | Yes | *Bacteria* | *Bacillota_B* | 109 | 17 | 1 | 4 | 0 | 5 |
| 10 | Yes | *Bacteria* | *Bacteroidota* | 160 | 106 | 1 | 4 | 1 | 199 |
| 11 | Yes | *Bacteria* | *Bacteroidota* | 110 | 50 | 2 | 4 | 0 | 40 |
| 12 | Yes | *Bacteria* | *Bacteroidota* | 153 | 73 | 2 | 5 | 2 | 69 |
| 13 | Yes | *Bacteria* | *Bacteroidota* | 96 | 28 | 1 | 4 | 0 | 10 |
| 14 | Yes | *Bacteria* | *Bacteroidota* | 91 | 29 | 1 | 3 | 1 | 10 |
| 15 | Yes | *Bacteria* | *Bacteroidota* | 180 | 135 | 2 | 5 | 2 | 209 |
| 16 | Yes | *Bacteria* | *Bacteroidota* | 173 | 87 | 1 | 5 | 2 | 94 |
| 17 | Yes | *Bacteria* | *Bacteroidota* | 108 | 53 | 0 | 3 | 1 | 51 |
| 18 | Yes | *Bacteria* | *Bacteroidota* | 91 | 27 | 2 | 3 | 1 | 9 |
| 19 | Yes | *Bacteria* | *Bacteroidota* | 198 | 127 | 1 | 5 | 2 | 192 |
| 20 | Yes | *Bacteria* | *Bacteroidota* | 181 | 113 | 1 | 5 | 2 | 143 |
| 21 | Yes | *Bacteria* | *Bacteroidota* | 184 | 92 | 1 | 5 | 1 | 122 |
| 22 | Yes | *Bacteria* | *Bacteroidota* | 100 | 34 | 1 | 4 | 0 | 15 |
| 23 | Yes | *Bacteria* | *Bacteroidota* | 190 | 124 | 1 | 5 | 2 | 161 |
| 24 | Yes | *Bacteria* | *Bacteroidota* | 112 | 53 | 2 | 5 | 2 | 53 |
| 25 | Yes | *Bacteria* | *Bacteroidota* | 182 | 125 | 2 | 5 | 2 | 147 |
| 26 | Yes | *Bacteria* | *Bacteroidota* | 103 | 32 | 1 | 5 | 0 | 18 |
| 27 | Yes | *Bacteria* | *Bacteroidota* | 190 | 103 | 2 | 5 | 2 | 158 |
| 28 | Yes | *Bacteria* | *Bacteroidota* | 159 | 71 | 1 | 5 | 1 | 92 |
| 29 | Yes | *Bacteria* | *Bacteroidota* | 211 | 161 | 2 | 5 | 2 | 304 |
| 30 | Yes | *Bacteria* | *Bacteroidota* | 106 | 42 | 2 | 4 | 1 | 29 |
| 31 | Yes | *Bacteria* | *Bacteroidota* | 159 | 57 | 1 | 5 | 1 | 56 |
| 32 | Yes | *Bacteria* | *Bacteroidota* | 152 | 92 | 1 | 4 | 2 | 139 |
| 33 | Yes | *Bacteria* | *Bacteroidota* | 153 | 98 | 1 | 4 | 1 | 153 |
| 34 | Yes | *Bacteria* | *Bacteroidota* | 167 | 144 | 1 | 5 | 3 | 260 |
| 35 | Yes | *Bacteria* | *Bacteroidota* | 100 | 30 | 1 | 5 | 0 | 17 |
| 36 | Yes | *Bacteria* | *Bacteroidota* | 98 | 32 | 1 | 4 | 1 | 14 |
| 37 | Yes | *Bacteria* | *Chloroflexota* | 213 | 100 | 1 | 5 | 1 | 123 |
| 38 | Yes | *Bacteria* | *Desulfobacterota* | 123 | 30 | 2 | 3 | 0 | 20 |
| 39 | Yes | *Bacteria* | *Desulfobacterota_F* | 120 | 38 | 1 | 4 | 1 | 26 |
| 40 | Yes | *Bacteria* | *Desulfobacterota_F* | 145 | 38 | 2 | 3 | 1 | 22 |
| 41 | Yes | *Bacteria* | *Desulfobacterota_F* | 134 | 35 | 2 | 3 | 0 | 19 |
| 42 | Yes | *Bacteria* | *Desulfobacterota_F* | 149 | 37 | 3 | 3 | 0 | 25 |
| 43 | Yes | *Bacteria* | *Desulfobacterota_F* | 143 | 39 | 2 | 4 | 0 | 28 |
| 44 | Yes | *Bacteria* | *Desulfobacterota_F* | 137 | 37 | 3 | 3 | 0 | 22 |
| 45 | Yes | *Bacteria* | *Myxococcota* | 163 | 67 | 1 | 3 | 3 | 47 |
| 46 | Yes | *Bacteria* | *Myxococcota* | 136 | 54 | 3 | 4 | 4 | 33 |
| 47 | Yes | *Bacteria* | *Pseudomonadota* | 237 | 47 | 2 | 2 | 2 | 35 |
| 48 | Yes | *Bacteria* | *Pseudomonadota* | 252 | 55 | 3 | 4 | 2 | 52 |
| 49 | Yes | *Bacteria* | *Pseudomonadota* | 136 | 36 | 2 | 2 | 0 | 22 |
| 50 | Yes | *Bacteria* | *Pseudomonadota* | 173 | 33 | 3 | 2 | 2 | 15 |
| 51 | Yes | *Bacteria* | *Pseudomonadota* | 183 | 42 | 2 | 3 | 2 | 20 |
| 52 | Yes | *Bacteria* | *Pseudomonadota* | 218 | 55 | 3 | 4 | 2 | 55 |
| 53 | Yes | *Bacteria* | *Pseudomonadota* | 213 | 58 | 2 | 5 | 2 | 58 |
| 54 | Yes | *Bacteria* | *Pseudomonadota* | 143 | 33 | 3 | 3 | 1 | 13 |
| 55 | Yes | *Bacteria* | *Pseudomonadota* | 178 | 42 | 2 | 4 | 2 | 21 |
| 56 | Yes | *Bacteria* | *Pseudomonadota* | 145 | 33 | 1 | 3 | 1 | 18 |
| 57 | Yes | *Bacteria* | *Pseudomonadota* | 269 | 57 | 3 | 4 | 2 | 44 |
| 58 | Yes | *Bacteria* | *Pseudomonadota* | 184 | 27 | 3 | 2 | 1 | 11 |
| 59 | Yes | *Bacteria* | *Pseudomonadota* | 152 | 27 | 2 | 3 | 1 | 13 |
| 60 | Yes | *Bacteria* | *Pseudomonadota* | 132 | 27 | 3 | 2 | 0 | 14 |
| 61 | Yes | *Bacteria* | *Pseudomonadota* | 129 | 33 | 2 | 2 | 2 | 16 |
| 62 | Yes | *Bacteria* | *Pseudomonadota* | 135 | 36 | 1 | 2 | 1 | 24 |
| 63 | Yes | *Bacteria* | *Pseudomonadota* | 186 | 46 | 2 | 4 | 2 | 26 |
| 64 | Yes | *Bacteria* | *Pseudomonadota* | 204 | 36 | 3 | 2 | 2 | 20 |
| 65 | Yes | *Bacteria* | *Pseudomonadota* | 141 | 36 | 2 | 2 | 0 | 27 |
| 66 | Yes | *Bacteria* | *Pseudomonadota* | 122 | 36 | 1 | 2 | 0 | 29 |
| 67 | Yes | *Bacteria* | *Pseudomonadota* | 162 | 43 | 3 | 4 | 1 | 27 |
| 68 | Yes | *Bacteria* | *Pseudomonadota* | 189 | 41 | 3 | 3 | 2 | 24 |
| 69 | Yes | *Bacteria* | *Pseudomonadota* | 190 | 43 | 2 | 3 | 2 | 29 |
| 70 | Yes | *Bacteria* | *Pseudomonadota* | 209 | 70 | 3 | 4 | 3 | 72 |
| 71 | Yes | *Bacteria* | *Pseudomonadota* | 153 | 44 | 3 | 4 | 0 | 33 |
| 72 | Yes | *Bacteria* | *Pseudomonadota* | 124 | 28 | 2 | 3 | 1 | 15 |
| 73 | Yes | *Bacteria* | *Pseudomonadota* | 160 | 52 | 3 | 3 | 2 | 41 |
| 74 | Yes | *Bacteria* | *Pseudomonadota* | 182 | 44 | 3 | 2 | 2 | 21 |
| 75 | Yes | *Bacteria* | *Pseudomonadota* | 175 | 36 | 3 | 2 | 0 | 19 |
| 76 | Yes | *Bacteria* | *Pseudomonadota* | 166 | 42 | 2 | 4 | 1 | 23 |
| 77 | Yes | *Bacteria* | *Pseudomonadota* | 127 | 29 | 1 | 2 | 1 | 13 |
| 78 | Yes | *Bacteria* | *Pseudomonadota* | 168 | 51 | 3 | 4 | 2 | 45 |
| 79 | Yes | *Bacteria* | *Pseudomonadota* | 141 | 41 | 2 | 3 | 2 | 19 |
| 80 | Yes | *Bacteria* | *Pseudomonadota* | 194 | 53 | 3 | 5 | 2 | 47 |
| 81 | Yes | *Bacteria* | *Pseudomonadota* | 167 | 43 | 2 | 3 | 2 | 24 |
| 82 | Yes | *Bacteria* | *Pseudomonadota* | 185 | 38 | 3 | 2 | 2 | 23 |
| 83 | Yes | *Bacteria* | *Pseudomonadota* | 189 | 42 | 2 | 4 | 2 | 22 |
| 84 | Yes | *Bacteria* | *Pseudomonadota* | 167 | 31 | 2 | 2 | 2 | 15 |
| 85 | Yes | *Bacteria* | *Pseudomonadota* | 180 | 42 | 2 | 3 | 2 | 22 |
| 86 | Yes | *Bacteria* | *Pseudomonadota* | 247 | 64 | 1 | 6 | 0 | 64 |
| 87 | Yes | *Bacteria* | *Pseudomonadota* | 157 | 56 | 3 | 4 | 2 | 43 |
| 88 | Yes | *Bacteria* | *Pseudomonadota* | 144 | 30 | 0 | 1 | 2 | 13 |
| 89 | Yes | *Bacteria* | *Pseudomonadota* | 157 | 29 | 3 | 2 | 0 | 14 |
| 90 | Yes | *Bacteria* | *Pseudomonadota* | 136 | 31 | 0 | 2 | 2 | 15 |
| 91 | Yes | *Bacteria* | *Pseudomonadota* | 182 | 29 | 3 | 3 | 1 | 12 |
| 92 | Yes | *Bacteria* | *Pseudomonadota* | 170 | 36 | 2 | 3 | 0 | 16 |
| 93 | Yes | *Bacteria* | *Pseudomonadota* | 164 | 35 | 3 | 4 | 2 | 18 |
| 94 | Yes | *Bacteria* | *Pseudomonadota* | 154 | 48 | 4 | 4 | 0 | 36 |
| 95 | Yes | *Bacteria* | *Pseudomonadota* | 148 | 31 | 0 | 2 | 2 | 14 |
| 96 | Yes | *Bacteria* | *Pseudomonadota* | 153 | 52 | 3 | 4 | 2 | 28 |
| 97 | Yes | *Bacteria* | *Pseudomonadota* | 114 | 36 | 0 | 3 | 1 | 18 |
| 98 | Yes | *Bacteria* | *Pseudomonadota* | 133 | 41 | 1 | 4 | 2 | 20 |
| 99 | Yes | *Bacteria* | *Pseudomonadota* | 178 | 50 | 2 | 4 | 2 | 32 |
| 100 | Yes | *Bacteria* | *Pseudomonadota* | 146 | 31 | 2 | 2 | 1 | 15 |
| 101 | Yes | *Bacteria* | *Pseudomonadota* | 181 | 61 | 4 | 4 | 2 | 55 |
| 102 | Yes | *Bacteria* | *Pseudomonadota* | 190 | 45 | 2 | 4 | 1 | 24 |
| 103 | Yes | *Bacteria* | *Pseudomonadota* | 146 | 35 | 2 | 1 | 1 | 21 |
| 104 | Yes | *Bacteria* | *Pseudomonadota* | 181 | 81 | 4 | 5 | 2 | 87 |
| 105 | Yes | *Bacteria* | *Pseudomonadota* | 260 | 60 | 3 | 3 | 2 | 61 |
| 106 | Yes | *Bacteria* | *Pseudomonadota* | 160 | 35 | 1 | 4 | 2 | 19 |
| 107 | Yes | *Bacteria* | *Verrucomicrobiota* | 160 | 99 | 3 | 4 | 2 | 86 |
| 108 | Yes | *Bacteria* | *Verrucomicrobiota* | 179 | 120 | 1 | 4 | 1 | 152 |
| 109 | Yes | *Bacteria* | *Verrucomicrobiota* | 166 | 124 | 2 | 4 | 1 | 155 |
| 110 | Yes | *Bacteria* | *-* | 109 | 52 | 1 | 4 | 0 | 68 |
| 111 | No | *Bacteria* | *Acidobacteriota* | 139 | 41 | 1 | 5 | 2 | 27 |
| 112 | No | *Bacteria* | *Acidobacteriota* | 174 | 70 | 3 | 4 | 3 | 69 |
| 113 | No | *Bacteria* | *Bacillota* | 23 | 13 | 0 | 2 | 1 | 5 |
| 114 | No | *Bacteria* | *Bacillota* | 20 | 11 | 0 | 2 | 0 | 2 |
| 115 | No | *Bacteria* | *Bacillota* | 27 | 10 | 0 | 1 | 0 | 2 |
| 116 | No | *Bacteria* | *Bacillota* | 72 | 27 | 1 | 3 | 0 | 19 |
| 117 | No | *Bacteria* | *Bacillota_A* | 93 | 56 | 2 | 4 | 1 | 52 |
| 118 | No | *Bacteria* | *Bacillota_A* | 95 | 30 | 0 | 5 | 0 | 25 |
| 119 | No | *Bacteria* | *Bacteroidota* | 175 | 125 | 3 | 5 | 2 | 166 |
| 120 | No | *Bacteria* | *Bacteroidota* | 170 | 79 | 1 | 5 | 1 | 105 |
| 121 | No | *Bacteria* | *Bacteroidota* | 115 | 49 | 0 | 3 | 2 | 45 |
| 122 | No | *Bacteria* | *Bacteroidota* | 165 | 73 | 1 | 5 | 1 | 93 |
| 123 | No | *Bacteria* | *Bacteroidota* | 154 | 76 | 1 | 5 | 2 | 88 |
| 124 | No | *Bacteria* | *Bacteroidota* | 169 | 92 | 1 | 5 | 2 | 105 |
| 125 | No | *Bacteria* | *Bacteroidota* | 107 | 39 | 1 | 5 | 0 | 22 |
| 126 | No | *Bacteria* | *Bacteroidota* | 117 | 46 | 1 | 2 | 1 | 42 |
| 127 | No | *Bacteria* | *Bacteroidota* | 118 | 37 | 0 | 4 | 1 | 25 |
| 128 | No | *Bacteria* | *Bacteroidota* | 106 | 34 | 2 | 3 | 0 | 17 |
| 129 | No | *Bacteria* | *Bacteroidota* | 164 | 124 | 1 | 5 | 2 | 188 |
| 130 | No | *Bacteria* | *Bacteroidota* | 100 | 32 | 1 | 3 | 0 | 17 |
| 131 | No | *Bacteria* | *Bacteroidota* | 176 | 98 | 1 | 5 | 1 | 126 |
| 132 | No | *Bacteria* | *Bacteroidota* | 183 | 140 | 1 | 5 | 3 | 236 |
| 133 | No | *Bacteria* | *Bacteroidota* | 178 | 129 | 2 | 5 | 3 | 177 |
| 134 | No | *Bacteria* | *Bacteroidota* | 189 | 110 | 3 | 5 | 2 | 141 |
| 135 | No | *Bacteria* | *Bacteroidota* | 137 | 56 | 1 | 5 | 1 | 53 |
| 136 | No | *Bacteria* | *Bacteroidota* | 117 | 46 | 0 | 2 | 0 | 47 |
| 137 | No | *Bacteria* | *Desulfobacterota_F* | 118 | 35 | 1 | 3 | 0 | 23 |
| 138 | No | *Bacteria* | *Myxococcota* | 172 | 119 | 4 | 3 | 3 | 160 |
| 139 | No | *Bacteria* | *Myxococcota* | 74 | 23 | 0 | 2 | 0 | 13 |
| 140 | No | *Bacteria* | *Planctomycetota* | 152 | 119 | 2 | 3 | 3 | 154 |
| 141 | No | *Bacteria* | *Pseudomonadota* | 145 | 29 | 1 | 3 | 1 | 30 |
| 142 | No | *Bacteria* | *Pseudomonadota* | 170 | 40 | 2 | 2 | 1 | 23 |
| 143 | No | *Bacteria* | *Pseudomonadota* | 143 | 34 | 1 | 2 | 2 | 16 |
| 144 | No | *Bacteria* | *Pseudomonadota* | 154 | 43 | 3 | 2 | 1 | 23 |
| 145 | No | *Bacteria* | *Pseudomonadota* | 138 | 36 | 2 | 2 | 2 | 14 |
| 146 | No | *Bacteria* | *Pseudomonadota* | 171 | 40 | 3 | 2 | 0 | 24 |
| 147 | No | *Bacteria* | *Pseudomonadota* | 148 | 38 | 1 | 3 | 2 | 23 |
| 148 | No | *Bacteria* | *Pseudomonadota* | 154 | 41 | 2 | 3 | 1 | 31 |
| 149 | No | *Bacteria* | *Pseudomonadota* | 135 | 30 | 3 | 4 | 2 | 19 |
| 150 | No | *Bacteria* | *Pseudomonadota* | 135 | 34 | 1 | 2 | 2 | 14 |
| 151 | No | *Bacteria* | *Pseudomonadota* | 149 | 32 | 4 | 2 | 2 | 24 |
| 152 | No | *Bacteria* | *Pseudomonadota* | 152 | 32 | 1 | 4 | 1 | 26 |
| 153 | No | *Bacteria* | *Pseudomonadota* | 170 | 51 | 2 | 4 | 1 | 34 |
| 154 | No | *Bacteria* | *Verrucomicrobiota* | 76 | 22 | 1 | 2 | 0 | 8 |
| 155 | No | *Bacteria* | *Verrucomicrobiota* | 130 | 50 | 2 | 4 | 2 | 48 |
| 156 | No | *Archaea* | *Thermoproteota* | 66 | 13 | 1 | 1 | 1 | 7 |
| 157 | No | *Archaea* | *Thermoproteota* | 73 | 14 | 2 | 1 | 0 | 7 |
| 158 | No | *Archaea* | *Halobacteriota* | 135 | 15 | 2 | 2 | 0 | 11 |

**Table S12** The information of 62 non-redundant high-quality MAGs (completeness ≥ 90% and contamination ≤ 5%) in HMW soil in microcosm experiments.

| ID | ARG | Domain | Phylum | CMG | CAZyme | Lignin | Chitin | Cellulose | GH |
| --- | --- | --- | --- | --- | --- | --- | --- | --- | --- |
| 1 | Yes | *Bacteria* | *Bacteroidota* | 154 | 80 | 2 | 5 | 2 | 77 |
| 2 | Yes | *Bacteria* | *Verrucomicrobiota* | 104 | 51 | 1 | 5 | 1 | 27 |
| 3 | Yes | *Bacteria* | *Nitrospirota* | 136 | 38 | 1 | 3 | 0 | 26 |
| 4 | Yes | *Bacteria* | *Chloroflexota* | 109 | 34 | 1 | 5 | 0 | 16 |
| 5 | Yes | *Bacteria* | *Acidobacteriota* | 150 | 51 | 2 | 4 | 3 | 31 |
| 6 | Yes | *Bacteria* | *Pseudomonadota* | 146 | 34 | 5 | 2 | 1 | 18 |
| 7 | Yes | *Bacteria* | *Pseudomonadota* | 194 | 35 | 2 | 3 | 2 | 23 |
| 8 | Yes | *Bacteria* | *Pseudomonadota* | 205 | 100 | 3 | 4 | 8 | 89 |
| 9 | Yes | *Bacteria* | *Pseudomonadota* | 201 | 37 | 3 | 3 | 1 | 20 |
| 10 | Yes | *Bacteria* | *Pseudomonadota* | 210 | 41 | 2 | 3 | 2 | 27 |
| 11 | Yes | *Bacteria* | *Pseudomonadota* | 189 | 47 | 2 | 4 | 1 | 36 |
| 12 | Yes | *Bacteria* | *Pseudomonadota* | 137 | 49 | 2 | 4 | 2 | 40 |
| 13 | Yes | *Bacteria* | *Pseudomonadota* | 149 | 22 | 3 | 1 | 1 | 11 |
| 14 | Yes | *Bacteria* | *Desulfobacterota* | 179 | 50 | 2 | 5 | 3 | 39 |
| 15 | Yes | *Bacteria* | *Bacteroidota* | 102 | 31 | 2 | 4 | 0 | 11 |
| 16 | Yes | *Bacteria* | *Acidobacteriota* | 177 | 84 | 3 | 4 | 3 | 85 |
| 17 | Yes | *Bacteria* | *Verrucomicrobiota* | 152 | 74 | 4 | 3 | 2 | 82 |
| 18 | Yes | *Bacteria* | *Pseudomonadota* | 162 | 35 | 1 | 1 | 0 | 23 |
| 19 | Yes | *Bacteria* | *Acidobacteriota* | 179 | 61 | 1 | 5 | 2 | 63 |
| 20 | Yes | *Bacteria* | *Pseudomonadota* | 166 | 96 | 3 | 4 | 4 | 84 |
| 21 | Yes | *Bacteria* | *Acidobacteriota* | 174 | 58 | 3 | 5 | 3 | 44 |
| 22 | Yes | *Bacteria* | *Pseudomonadota* | 166 | 50 | 3 | 3 | 2 | 30 |
| 23 | Yes | *Bacteria* | *Pseudomonadota* | 172 | 69 | 1 | 3 | 2 | 64 |
| 24 | Yes | *Bacteria* | *Bacteroidota* | 116 | 58 | 2 | 6 | 2 | 37 |
| 25 | Yes | *Bacteria* | *Pseudomonadota* | 180 | 33 | 3 | 3 | 2 | 18 |
| 26 | Yes | *Bacteria* | *Myxococcota* | 129 | 58 | 1 | 3 | 4 | 40 |
| 27 | Yes | *Bacteria* | *Pseudomonadota* | 239 | 54 | 3 | 4 | 2 | 46 |
| 28 | Yes | *Bacteria* | *Bacteroidota* | 111 | 50 | 2 | 4 | 0 | 41 |
| 29 | Yes | *Bacteria* | *Bacteroidota* | 162 | 136 | 1 | 5 | 4 | 204 |
| 30 | Yes | *Bacteria* | *Pseudomonadota* | 172 | 29 | 3 | 3 | 2 | 20 |
| 31 | Yes | *Bacteria* | *Pseudomonadota* | 171 | 27 | 3 | 3 | 1 | 13 |
| 32 | Yes | *Bacteria* | *Pseudomonadota* | 198 | 53 | 2 | 3 | 1 | 35 |
| 33 | Yes | *Bacteria* | *Pseudomonadota* | 180 | 38 | 3 | 2 | 0 | 20 |
| 34 | No | *Bacteria* | *Pseudomonadota* | 125 | 37 | 2 | 3 | 2 | 22 |
| 35 | No | *Bacteria* | *Pseudomonadota* | 159 | 30 | 1 | 4 | 1 | 23 |
| 36 | No | *Bacteria* | *Acidobacteriota* | 93 | 35 | 1 | 4 | 1 | 22 |
| 37 | No | *Bacteria* | *Verrucomicrobiota* | 113 | 37 | 1 | 5 | 1 | 21 |
| 38 | No | *Bacteria* | *Desulfobacterota* | 143 | 42 | 2 | 4 | 2 | 29 |
| 39 | No | *Bacteria* | *Eisenbacteria* | 104 | 44 | 3 | 4 | 2 | 20 |
| 40 | No | *Bacteria* | *Pseudomonadota* | 120 | 38 | 1 | 1 | 2 | 20 |
| 41 | No | *Bacteria* | *Actinomycetota* | 125 | 34 | 1 | 4 | 3 | 29 |
| 42 | No | *Bacteria* | *Bdellovibrionota* | 91 | 27 | 1 | 3 | 1 | 18 |
| 43 | No | *Bacteria* | *Actinomycetota* | 116 | 34 | 0 | 3 | 2 | 19 |
| 44 | No | *Bacteria* | *Gemmatimonadota* | 94 | 31 | 0 | 5 | 3 | 19 |
| 45 | No | *Bacteria* | *Planctomycetota* | 173 | 51 | 4 | 3 | 1 | 54 |
| 46 | No | *Bacteria* | *FCPU426* | 94 | 48 | 0 | 3 | 2 | 40 |
| 47 | No | *Bacteria* | *Gemmatimonadota* | 128 | 44 | 1 | 4 | 4 | 37 |
| 48 | No | *Bacteria* | *Actinomycetota* | 107 | 19 | 0 | 1 | 2 | 13 |
| 49 | No | *Bacteria* | *Bacillota* | 105 | 23 | 1 | 4 | 1 | 11 |
| 50 | No | *Bacteria* | *Bdellovibrionota* | 90 | 25 | 1 | 2 | 0 | 20 |
| 51 | No | *Bacteria* | *Gemmatimonadota* | 115 | 41 | 1 | 6 | 3 | 32 |
| 52 | No | *Bacteria* | *Pseudomonadota* | 178 | 57 | 4 | 4 | 3 | 42 |
| 53 | No | *Bacteria* | *Gemmatimonadota* | 99 | 31 | 1 | 4 | 2 | 19 |
| 54 | No | *Bacteria* | *Planctomycetota* | 92 | 38 | 1 | 2 | 0 | 24 |
| 55 | No | *Bacteria* | *Bacteroidota* | 103 | 41 | 1 | 4 | 1 | 33 |
| 56 | No | *Bacteria* | *Bacillota* | 116 | 16 | 3 | 3 | 0 | 6 |
| 57 | No | *Bacteria* | *Verrucomicrobiota* | 164 | 147 | 2 | 4 | 4 | 228 |
| 58 | No | *Bacteria* | *Pseudomonadota* | 95 | 24 | 2 | 2 | 0 | 11 |
| 59 | No | *Archaea* | *Thermoproteota* | 67 | 13 | 1 | 2 | 1 | 11 |
| 60 | No | *Archaea* | *Thermoproteota* | 70 | 16 | 1 | 1 | 0 | 9 |
| 61 | No | *Archaea* | *Thermoproteota* | 65 | 16 | 1 | 1 | 0 | 13 |
| 62 | No | *Archaea* | *Thermoproteota* | 69 | 16 | 2 | 1 | 0 | 15 |

**Table S13** Number of carbon-related genes in co-hosts and non-hosts in HMW soil (microcosm experiments).

| Type | Number (Median value) | | *P* value  (Wilcoxon rank sum test) |
| --- | --- | --- | --- |
|  | Co-hosts | Non-hosts |  |
| CMG | 166 | 105 | 4.90e-07 |
| CAZyme | 50 | 34 | 4.1e-04 |
| GH enzyme | 35 | 20 | 6.4e-03 |
| Chitin enzyme | 4 | 3 | 0.21 |
| Lignin enzyme | 2 | 1 | 5.7e-04 |
| Cellulose enzyme | 2 | 1 | 0.38 |

**Table S14** Host prediction of viruses at phylum level in LMW (HN) soil.

| Domain | Phylum | Number |
| --- | --- | --- |
| *Archaea* | *Halobacteriota* | 6 |
|  | *Asgardarchaeota* | 3 |
|  | *Methanobacteriota* | 3 |
|  | *Nanoarchaeota* | 3 |
|  | *Thermoplasmatota* | 1 |
|  | *Thermoproteota* | 2 |
| *Bacteria* | *Pseudomonadota* | 407 |
|  | *Bacillota* | 223 |
|  | *Bacteroidota* | 125 |
|  | *Planctomycetota* | 52 |
|  | *Actinomycetota* | 25 |
|  | *Acidobacteriota* | 16 |
|  | *Desulfobacterota* | 16 |
|  | *Myxococcota* | 8 |
|  | *Patescibacteria* | 6 |
|  | *Verrucomicrobiota* | 3 |
|  | *Chloroflexota* | 2 |
|  | *Bdellovibrionota* | 1 |
|  | *Hydrogenedentota* | 1 |

**Table S15** Host prediction of viruses at phylum level in HMW (FJ) soil.

| Domain | Phylum | Number |
| --- | --- | --- |
| *Archaea* | *Halobacteriota* | 1 |
|  | *Methanobacteriota* | 3 |
|  | *Thermoplasmatota* | 1 |
|  | *Thermoproteota* | 1 |
| *Bacteria* | *Acidobacteriota* | 6 |
|  | *Actinomycetota* | 30 |
|  | *Bacillota* | 54 |
|  | *Bacteroidota* | 11 |
|  | *Bdellovibrionota* | 1 |
|  | *Chloroflexota* | 3 |
|  | *Cyanobacteriota* | 1 |
|  | *Desulfobacterota_D* | 1 |
|  | *Gemmatimonadota* | 1 |
|  | *Nitrospirota* | 2 |
|  | *Planctomycetota* | 1 |
|  | *Pseudomonadota* | 226 |
|  | *Verrucomicrobiota* | 14 |

**Table S16** Random sample selection for microbial co-occurrence network analysis.

| Randomization1 | | Randomization2 | | Randomization3 | | Randomization4 | | Randomization5 | |
| --- | --- | --- | --- | --- | --- | --- | --- | --- | --- |
| GZ_KY_1 | LMW | GD_LM_1 | LMW | HN_LY_3 | LMW | HB_JZ_3 | LMW | ZJ_HZ_1 | LMW |
| AH_HN_3 | LMW | ZJ_QJ_1 | LMW | GX_GL_2 | LMW | ZJ_TZ_1 | LMW | GX_GL_1 | LMW |
| YN_DC_3 | LMW | GZ_XR_2 | LMW | GZ_KY_1 | LMW | GD_LM_1 | LMW | HN_HY_3 | LMW |
| HN_HH_3 | LMW | HB_JZ_2 | LMW | HLJ_YL_3 | LMW | JX_NC_3 | LMW | LN_LY_1 | LMW |
| HLJ_YL_1 | LMW | HLJ_YL_2 | LMW | HN_HY_1 | LMW | HB_JZ_2 | LMW | YN_DC_1 | LMW |
| ZJ_QJ_3 | LMW | ZJ_TZ_3 | LMW | ZJ_SY_2 | LMW | HN_HY_2 | LMW | HN_HH_2 | LMW |
| HN_YY_2 | LMW | HB_JZ_3 | LMW | GD_SG_1 | LMW | AH_HN_3 | LMW | SD_TC_1 | LMW |
| HN_CZ_1 | LMW | HN_HY_3 | LMW | YN_DC_3 | LMW | HLJ_YL_1 | LMW | ZJ_QJ_2 | LMW |
| AH_HN_1 | LMW | AH_HN_2 | LMW | GD_LM_3 | LMW | GZ_KY_2 | LMW | HN_CZ_3 | LMW |
| GX_GL_1 | LMW | YN_DC_1 | LMW | GD_LM_1 | LMW | SD_TC_1 | LMW | HN_CZ_2 | LMW |
| YN_CJ_3 | LMW | GZ_KY_1 | LMW | ZJ_HZ_3 | LMW | HN_HH_1 | LMW | YN_CJ_1 | LMW |
| HB_JZ_1 | LMW | GZ_XR_3 | LMW | JX_NC_1 | LMW | JL_SL_3 | LMW | HN_XX_3 | LMW |
| JL_JH_1 | LMW | ZJ_SY_2 | LMW | LN_LY_1 | LMW | GZ_TR_2 | LMW | SD_TC_3 | LMW |
| GZ_XR_1 | LMW | GZ_TR_3 | LMW | YN_MG_2 | LMW | HN_CZ_2 | LMW | GZ_XR_2 | LMW |
| HLJ_YL_2 | LMW | JL_SL_1 | LMW | GZ_XR_3 | LMW | HLJ_YL_3 | LMW | ZJ_HZ_2 | LMW |
| ZJ_HZ_1 | LMW | HN_CZ_3 | LMW | LN_LY_3 | LMW | SD_TC_3 | LMW | GD_LM_3 | LMW |
| HN_CZ_2 | LMW | HN_HH_3 | LMW | JL_JH_3 | LMW | AH_HN_1 | LMW | ZJ_SY_3 | LMW |
| HN_HY_1 | LMW | JL_JH_3 | LMW | GZ_XR_2 | LMW | AH_HN_2 | LMW | HN_HH_3 | LMW |
| HB_WH_3 | LMW | GD_LM_3 | LMW | HB_JZ_2 | LMW | HB_JZ_1 | LMW | HLJ_YL_1 | LMW |
| GD_SG_3 | LMW | HN_XX_3 | LMW | ZJ_SY_3 | LMW | GD_LM_2 | LMW | ZJ_QJ_1 | LMW |
| ZJ_SY_2 | LMW | JL_SL_2 | LMW | JL_SL_3 | LMW | JL_SL_1 | LMW | GZ_XR_1 | LMW |
| ZJ_HZ_2 | LMW | YN_DC_2 | LMW | HB_WH_2 | LMW | HN_LY_3 | LMW | HN_LY_2 | LMW |
| SD_TC_1 | LMW | AH_CZ_1 | LMW | ZJ_QJ_3 | LMW | GZ_KY_3 | LMW | HN_HY_2 | LMW |
| YN_DC_2 | LMW | HN_HY_1 | LMW | JL_JH_2 | LMW | HN_HY_3 | LMW | HB_WH_1 | LMW |
| HLJ_DQ_2 | HMW | JL_CC_3 | HMW | ZJ_QZ_3 | HMW | JS_SZ_1 | HMW | JL_CC_2 | HMW |
| JL_CC_2 | HMW | ZJ_QZ_3 | HMW | HLJ_DQ_1 | HMW | FJ_HT_3 | HMW | TJ_JN_1 | HMW |
| JS_SZ_2 | HMW | ZJ_QZ_1 | HMW | TJ_JN_1 | HMW | GD_XH_1 | HMW | JX_GZ_3 | HMW |
| GD_XH_1 | HMW | HN_ZZ_1 | HMW | GX_TX_2 | HMW | SH_MH_2 | HMW | TJ_JN_3 | HMW |
| AH_SZ_3 | HMW | HN_JY_1 | HMW | HN_ZZ_1 | HMW | YN_YL_3 | HMW | JX_GZ_1 | HMW |
| HN_SM_2 | HMW | HN_SM_3 | HMW | HN_ZZ_2 | HMW | HN_JY_2 | HMW | ZJ_QZ_2 | HMW |
| HLJ_DQ_3 | HMW | GX_TX_2 | HMW | GD_XH_1 | HMW | TJ_JN_3 | HMW | JS_SZ_3 | HMW |
| HN_LH_3 | HMW | HN_LH_2 | HMW | ZJ_JH_2 | HMW | JL_CC_1 | HMW | HN_LH_1 | HMW |
| JX_GZ_1 | HMW | SH_MH_1 | HMW | HN_SM_2 | HMW | SH_MH_1 | HMW | GX_TX_3 | HMW |
| GD_XH_2 | HMW | AH_SZ_3 | HMW | ZJ_JH_1 | HMW | ZJ_QZ_1 | HMW | JS_YX_1 | HMW |
| GX_TX_1 | HMW | JL_CC_2 | HMW | JS_SZ_2 | HMW | HN_LH_2 | HMW | HN_LH_3 | HMW |
| SH_MH_3 | HMW | JS_YX_3 | HMW | HN_LH_2 | HMW | HN_JY_3 | HMW | HN_JY_2 | HMW |
| JS_YX_1 | HMW | HN_SM_1 | HMW | HN_SM_3 | HMW | JL_CC_2 | HMW | YN_YL_1 | HMW |
| TJ_JN_2 | HMW | YN_YL_2 | HMW | GD_XH_3 | HMW | ZJ_JH_1 | HMW | JL_CC_3 | HMW |
| HN_SM_1 | HMW | YN_YL_3 | HMW | JX_GZ_1 | HMW | JS_SZ_3 | HMW | JS_YX_2 | HMW |
| GX_TX_3 | HMW | GD_XH_2 | HMW | YN_YL_1 | HMW | AH_SZ_2 | HMW | SH_MH_2 | HMW |
| HN_LH_2 | HMW | HN_LH_3 | HMW | HLJ_DQ_2 | HMW | JX_GZ_3 | HMW | HN_ZZ_1 | HMW |
| HN_JY_2 | HMW | AH_SZ_2 | HMW | FJ_HT_3 | HMW | HLJ_DQ_1 | HMW | AH_SZ_3 | HMW |
| JS_YX_2 | HMW | HLJ_DQ_1 | HMW | HN_ZZ_3 | HMW | HN_SM_3 | HMW | HN_JY_3 | HMW |
| JL_CC_3 | HMW | FJ_HT_3 | HMW | AH_SZ_1 | HMW | AH_SZ_3 | HMW | JS_SZ_2 | HMW |
| YN_YL_3 | HMW | JX_GZ_3 | HMW | TJ_JN_3 | HMW | TJ_JN_1 | HMW | ZJ_JH_1 | HMW |
| HN_SM_3 | HMW | JS_SZ_2 | HMW | HN_JY_2 | HMW | JX_GZ_2 | HMW | SH_MH_1 | HMW |
| YN_YL_1 | HMW | HN_ZZ_2 | HMW | JL_CC_2 | HMW | FJ_HT_2 | HMW | HN_ZZ_2 | HMW |
| ZJ_JH_1 | HMW | FJ_HT_1 | HMW | JS_YX_1 | HMW | ZJ_QZ_3 | HMW | HLJ_DQ_1 | HMW |

**Reference**

1.	Chen Q, Chen F, Gonsior M et al. Correspondence between DOM molecules and microbial community in a subtropical coastal estuary on a spatiotemporal scale. *Environ Int* 2021;**154**:106558. https://doi.org/10.1016/j.envint.2021.106558

2.	Wilske C, Herzsprung P, Lechtenfeld OJ et al. Photochemically induced changes of dissolved organic matter in a humic-rich and forested stream. *Water* 2020;**12**:331. https://doi.org/10.3390/w12020331

3.	Fu L, Wang J, Deng L et al. Bridging Dissolved organic matter reactivity to ozonation catalysts for Cu@Al_2_O_3_ from the molecular level by machine learning. *Environ Sci Technol* 2025;**59**:23568–23580. https://doi.org/10.1021/acs.est.5c07324

4.	Aldeguer-Riquelme B, Rodriguez-R LM, Konstantinidis KT. Differences in metagenome coverage may confound abundance-based and diversity conclusions and how to deal with them. *ISME Commun* 2025;**5**:ycaf140. https://doi.org/10.1093/ismeco/ycaf140

5.	Lindner BG, Gerhardt K, Feistel DJ et al. A user’s guide to the bioinformatic analysis of shotgun metagenomic sequence data for bacterial pathogen detection. *Int J Food Microbiol* 2024;**410**:110488. https://doi.org/10.1016/j.ijfoodmicro.2023.110488

6.	Aroney STN, Newell RJP, Nissen JN et al. CoverM: Read alignment statistics for metagenomics. *Bioinformatics* 2025;**41**:btaf147.

7.	Castro JC, Rodriguez-R LM, Harvey WT et al. imGLAD: accurate detection and quantification of target organisms in metagenomes. *PeerJ* 2018;**6**:e5882. https://doi.org/10.7717/peerj.5882

8.	Levins R. Evolution in changing environments: some theoretical explorations. (MPB-2). Princeton University Press, 1968.

9.	Yan H, Zhang D, Wang K. Partitioning of resource states in the marine environment and calculation of niche breadth of microbial community. *Bio-protocol* 2022;e2003391. https://doi.org/10.21769/BioProtoc.2003391

10.	Fujita H, Ushio M, Suzuki K et al. Metagenomic analysis of ecological niche overlap and community collapse in microbiome dynamics. *Front Microbiol* 2023;**14**:1261137. https://doi.org/10.3389/fmicb.2023.1261137

11.	Bolger AM, Lohse M, Usadel B. Trimmomatic: a flexible trimmer for Illumina sequence data. *Bioinformatics* 2014;**30**:2114–2120. https://doi.org/10.1093/bioinformatics/btu170

12.	Kopylova E, Noé L, Touzet H. SortMeRNA: fast and accurate filtering of ribosomal RNAs in metatranscriptomic data. *Bioinformatics* 2012;**28**:3211–3217. https://doi.org/10.1093/bioinformatics/bts611

13.	Guo J, Vik D, Pratama AA et al. Viral sequence identification SOP with VirSorter2. 2021. https://dx.doi.org/10.17504/protocols.io.bwm5pc86

14.	Shen R, Pan B, Peng Q et al. High-resolution distribution maps of single-season rice in China from 2017 to 2022. *Earth Syst Sci Data* 2023;**15**:3203–3222. https://doi.org/10.5194/essd-15-3203-2023

15.	Jain C, Rodriguez-R LM, Phillippy AM et al. High throughput ANI analysis of 90K prokaryotic genomes reveals clear species boundaries. *Nat Commun* 2018;**9**:5114. https://doi.org/10.1038/s41467-018-07641-9
